# *In silico* analyses of leptin and leptin receptor of spotted snakehead *Channa punctata*

**DOI:** 10.1371/journal.pone.0270881

**Published:** 2022-07-07

**Authors:** Amrita Bakshi, Umesh Rai

**Affiliations:** Department of Zoology, University of Delhi, Delhi, India; Universite de Rouen, FRANCE

## Abstract

The present study, in addition to molecular characterization of leptin (*lepa*) and its receptor (*lepr*) of spotted snakehead *Channa punctata*, is focussed on physicochemical, structural, evolutionary and selection pressure analyses which are poorly elucidated in teleosts in spite of that existence of these genes is well reported in several fish species. The putative full-length Lep and Lepr of *C*. *punctata* showed conserved structural and functional domains, especially the residues responsible for structural integrity and signal transduction. Conversely, residues predicted essential for Lep-Lepr interaction displayed divergence between teleosts and tetrapods. Impact of substitutions/deletions predicted using protein variation effect analyser tool highlighted species specificity in ligand-receptor interaction. Physicochemical properties of ligand and receptor predicted for the first time in vertebrates revealed high aliphatic and instability indices for both Lepa and Lepr, indicating thermostability of proteins but their instability under ex vivo conditions. Positive grand average of hydropathy score of Lepa suggests its hydrophobic nature conjecturing existence of leptin binding proteins in *C*. *punctata*. In addition to disulphide bonding, a novel posttranslational modification (S-126 phosphorylation) was predicted in Lepa of *C*. *punctata*. In Lepr, disulphide bond formation and N-linked glycosylation near WSXWS motif in ECD, and phosphorylation at tyrosine residues in ICD were predicted. Leptin and its receptor sequence of *C*. *punctata* cladded with its homolog from *C*. *striata* and *C*. *argus* of order Anabantiformes. Leptin system of Anabantiformes was phylogenetically closer to that of Pleuronectiformes, Scombriformes and Perciformes. Selection pressure analysis showed higher incidence of negative selection in teleostean leptin genes indicating limited adaptation in their structure and function. However, evidence of pervasive and episodic diversifying selection laid a foundation of co-evolution of Lepa and Lepr in teleosts.

## Introduction

The snakeheads also known as murrels are highly priced due to nutritional and medicinal importance. To encourage their cultivation, Indian Council of Agricultural Research has started producing their hatchlings [1]. Nonetheless, slow growth rate, poor fecundity and cannibalism discourage farmers from extensive culturing of murrels. Henceforth, the current study is focussed on leptin system which plays critical role in regulating feeding behaviour, reproduction and energy homeostasis in vertebrates [2]. In teleosts, existence of leptin (*lep*) has been first demonstrated in pufferfish *Takifugu rubripes* [3] and leptin receptor (*lepr*) in medaka *Oryzias melastigma* [4]. Thereafter, several studies have reported the presence of *lep* and *lepr* in a number of fishes belonging to family Salmonidae, Cyprinidae, Moronidae, Scophthalmidae, Syngnathidae and Scombridae [5, 6]. In case of Channidae, a single study is available on *lep* [7] while no report exists with regard to *lepr*. Thereby, molecular characterization and computational analysis of leptin and leptin receptor was carried in spotted snakehead *Channa punctata*. The *Channa punctata* (Bloch, 1793) is a member of family Channidae (Fowler, 1934) which is classified under suborder Channoidei (Berg, 1940). The suborders Anabantidae (Berg, 1940) and Channoidei are recognized under the order of freshwater ray-finned fishes, Anabantiformes. This order, consisting of at least 207 fish species, belongs to a monophyletic clade which is a sister clade of orders Synbranchiformes, Pleuronectiformes, Carangiformes and Istiophoriformes [8, 9].

The notion of evolution of leptin and leptin receptor emerges from the fact that leptin system is present across the vertebrates. Leptin genes are highly divergent in teleosts due to genome duplication, a key event responsible for increase in gene repertoire [10]. A number of fishes belonging to order Cichliformes, Perciformes and Beloniformes possess two paralogs of leptin (*lepa*, *lepb*) [11–13] while four paralogs of *lep* (*lepa1*, *lepa2*, *lepb1*, *lepb2*) exist in Cypriniformes and Salmoniformes fishes [14–16]. Among Anabantiformes, two leptin paralogs, *lepa* and *lepb*, have been reported in Northern snakehead *Channa argus* (7; nucleotide percentage identity between *lepa* and *lepb* = 44.76%, Clustal Omega) though only *lepa* has been demonstrated so far in the available transcriptome data of stripped snakehead *Channa striata* [17]. One of the paralogs is believed to be lost during the course of evolution as a single *lep* gene is demonstrated in the genome of Tetraodontiformes [3, 11]. Interestingly, low amino acid similarity (18% to 30%) is reported between *lepa* and *lepb* of a same species [2]. The paralogs of *lepr* are reported only in European eel *Anguilla anguilla* [18] and Asian arowana *Scleropages formosus* (Accession No. XP018609810 and KPP63040) and its isoforms have been demonstrated in Atlantic salmon *Salmo salar* [16], crucian carp *Carassius carassius* [19] and rainbow trout *Oncorhynchus mykiss* [20]. Regarding factors implicated in driving evolution of leptin, several reports are available in mammals [21–24] while least attention has been paid to non-mammalian vertebrates [6]. In teleosts, a single study exists in which negative selection pressure is implicated in evolution of leptin gene [25]. Surprisingly, study on factors directing molecular evolution of leptin receptor gene is meagre in mammals [23, 26] while completely missing in fishes. Henceforth, the current study was undertaken to examine the conservation of residues/domains in leptin and its receptor for developing better understanding of functional and evolutionary aspects of leptin system in fishes. Also, selection pressure analysis was carried out to denote the sites or branches undergoing diversifying/purifying selection that would have shaped the evolution of teleostean leptin system.

## Material and methods

### Sequence retrieval, transcript identification and sequence validation

Testicular transcriptome data of *C*. *punctata* (NCBI bioproject accession number PRJNA304088) [27] annotated using protein sequence database of rat *Rattus norvegicus* and fishes *Takifugu rubripes* and *Oreochromis niloticus* was used to retrieve the longest nucleotide sequences of *lep* and *lepr*. Also, the transcriptome data of *C*. *punctata* was annotated using nucleotide (NCBI accession No. MK559418.1; 7) and protein (NCBI accession No. ASW18438.1; 7) sequences of *lepb* of *C*. *argus*. The selected transcript of *lep* showed highest percentage identity with sequence of *lepa* from *T*. *rubripes* while *lepr* with that of *R*. *norvegicus*. Thereafter, open reading frame (ORF) finder [28] was applied to find coding sequence (cds) of *lepa* and *lepr*. Nucleotide BLAST (BLASTn) analysis revealed that the transcript of *lepr* encoded partial sequence at 5’ end while of *lepa* encoded a putative full-length sequence.

### Cloning of leptin receptor (full-length *lepr*)

For rapid amplification of cDNA to obtain full-length sequence of *lepr* of *C*. *punctata*, 5’ RACE primer [5’ CGCTCTCATCCTCTACCCACAAGTC 3’; annealing temperature = 68.3 ^ο^C; product length = 644 base pairs (bp)] was designed using Gene Runner, Hastings software Inc. [29]. To make cDNA, SMARTer^TM^ RACE cDNA amplification kit [# 684924, Clontech, Takara Biotechnology (Dalian) Co., Ltd.] was used following manufacturer’s instructions. Briefly, 2 μl first strand buffer (5X), 1 μl dithiothreitol (20 mM) and 1 μl dNTP mix (10 mM) were mixed in tube A. After incubation at room temperature, RNase inhibitor (0.25 μl) and SMART Scribe reverse transcriptase (1 μl) were added. In another tube (tube B), 2 μg RNA isolated from midbrain of male *C*. *punctata* using TRI reagent was mixed with 1 μl 5’-cds primer A and total volume was adjusted to 3.75 μl with sterile water. The RNA containing tube B was incubated initially at 72 ^ο^C for 3 min and then at 42 ^ο^C for 2 min in a hot-lid thermocycler. After centrifugation at 14,000 g for 10 s, one microliter of SMART II^TM^ A oligonucleotide was added. After spinning, content of tube B was mixed with tube A and kept at 42 ^ο^C for 90 min followed by incubation at 70 ^ο^C for 10 min in a thermocycler. Finally, tricine-EDTA buffer (50 μl) was added and cDNA thus prepared was stored at -20 ^ο^C until further use.

To amplify nucleotide sequence from 5’ terminus of *lepr*, PCR was carried out using Advantage® 2PCR kit [#639207, Clontech, Takara Biotechnology (Dalian) Co., Ltd.]. A total of 10 μl PCR reaction mix comprising of 5 μl buffer (10X advantage 2 PCR), 1 μl dNTP mix (10 mM), 1 μl advantage 2 polymerase (50X), 1 μl universal primer mix (10X), 0.2 μl *lepr*-specific 5’ RACE primer and remaining volume of PCR-grade water was prepared. Thermocycling conditions adopted for PCR are enlisted as: preliminary denaturation (94 ^ο^C for 1 min), and thereafter 30 cycles of each, denaturation (94 ^ο^C for 30 sec/ cycle), annealing (68.3 ^ο^C for 30 sec/ cycle) and extension (72 ^ο^C for 3 min/ cycle). For validation of cDNA, qPCR primers of *lepr* [30] were used as positive control. The amplified product was resolved by electrophoresis on 1% agarose gel containing 0.05% ethidium bromide and observed under ultraviolet illumination. To identify the length of DNA product, 100 base pair DNA ladder (Thermo Fisher Scientific, Massachusetts, USA) was run in a parallel well. The portion of agarose gel containing desirable length of product was excised and processed for elution of DNA using DNA elution kit, Wizard® SV Gel and PCR Clean-Up System (#A9281, Promega Corporation, Madison, USA). The eluted PCR product was ligated with pGEM-T vector using pGEM®-T easy vector system I kit (#A1360, Promega Corporation, Madison, USA) and transformed into competent *Escherichia coli DH5α* following heat shock method [31]. Thereafter, positive white colonies were picked and cultured in 10 ml Luria-Bertani medium (100 μg/ml ampicillin) for 6–8 h at 37 ^ο^C. Plasmid isolated from bacterial culture using commercially available kit, Wizard® Plus SV Minipreps DNA Purification System (#A1330, Promega Corporation, Madison, USA), was subjected to DNA-insert verification by conducting PCR using M13 universal primers (forward primer: 5’ GTT TTC CCA GTC ACG AC 3’; reverse primer: 5’ CAG GAA ACA GCT ATG AC 3’; T_a_ = 55 ^ο^C). Full-length *lepr* sequence achieved by compiling its sequence obtained from amplified product and partial transcript retrieved from transcriptome data of *C*. *punctata* was verified employing BLASTn bioinformatics tool.

### Primary, secondary and tertiary structural analyses of protein sequences

The predicted protein sequences of Lepa and Lepr was obtained using ExPASy translate tool [32]. Signal peptide in Lepa was predicted by SignalP 4.1 online software [33]. To predict the physicochemical properties including composition of amino acids, instability index [34], molecular weight, aliphatic index [35] and grand average of hydropathy (GRAVY) [34, 36], ExPASy ProtParam tool [32] was applied to primary protein sequence of Lepa and Lepr. The secondary structural features such as proportions of α-helices, β-sheets and turns were deduced using Phyre2 software [37] while hydropathicity was estimated by ExPASy ProtScale [36]. For comparative study of primary and secondary structural features, human LEP and LEPR (NCBI GenBank accession number AAH69452.1 and AAB09673.1, respectively) were also run in parallel. The tertiary structure of Lepa and extra- as well as intra-cellular domains of Lepr was generated using Phyre2 and quality of generated models was verified by PROCHECK analysis [38]. The ligand binding site in Lepr of *C*. *punctata* was predicted using 3DLigandSite [39].

### Evolutionary analyses

#### Sequence homology and domain analysis

The important structural and functional domains in Lepa and Lepr of *C*. *punctata* were predicted using simple modular architecture research tool (SMART) [40]. Also, functionally important residues and domains were predicted in Lepa and Lepr of *C*. *punctata* based on mutational studies in mammals [6, 41–50]. The conservation of predicted motifs and functionally important sites across vertebrates were analysed by generating multiple sequence alignment (MSA) using Clustal Omega [51]. The accession number of proteins used to generate MSA is provided as S2 Table. Using PROVEAN tool [52], effect of substitutions/deletions at functionally important sites were identified at a threshold score of -2.5 considering human LEP [53] and LEPR [54] as reference. Posttranslational modifications were found using motif scan [55].

#### Phylogenetic tree construction

To study the relatedness between different species with respect to leptin and its receptor, efforts were made to identify percentage identity between Lepa and Lepr of *C*. *punctata* and their respective orthologs in vertebrates using Clustal Omega [51]. In order to understand the evolutionary relationship, separate phylogenetic tree was constructed for leptin and leptin receptor. To achieve this, individual alignment files were prepared by aligning predicted full-length sequence of Lepa and Lepr of *C*. *punctata* with respective protein sequences from fishes belonging to different orders and representative of each class of vertebrates using Multiple Sequence Comparison by Log Expectation (MUSCLE) algorithm in Molecular Evolutionary Genetics Analysis (MEGA) 11 software [56]. In addition of Lepa, protein sequences of Lepb were also used. Thereafter, phylogenetic trees were constructed by employing maximum-likelihood method with Jones-Taylor-Thornton substitution model (MEGA 11 software). The reliability of the trees was assessed with 1000 bootstrap replicates. GenBank accession number of each protein sequence is provided in S2 Table.

#### Molecular evolutionary analysis

In order to study evolutionary pattern of leptin and leptin receptor in teleosts, selection pressure analysis was conducted. Concisely, separate databases were created for nucleotide sequence of open reading frame of leptin (*lepa*), and for extracellular domain and intracellular domain of leptin receptor of several fishes including *C*. *punctata*. The NCBI accession number of nucleotide sequences used to prepare these files is provided in S2 Table. Sequences of leptin and its receptor of other vertebrate classes were deliberately not included to avoid false positives. The stop codons were cleaned using CleanStopCodons.bf tool in HyPhy package [57] and ‘Universal genetic code’ was selected. This file was used for codon alignment employing MUSCLE algorithm (MEGA 11 software) for further analyses using web server Datamonkey [58, 59]. For detecting site-specific selection, fast, unconstrained Bayesian approximation (FUBAR) at posterior probability of 0.99 [60] was employed. Sudden change (episodic selection) in the codon sequence was recognized using mixed effects model of evolution (MEME; *p* = 0.05) [61]. Advanced branch-site Random Effects Likelihood (aBSREL; *p* ≤ 0.05) was used to identify individual branches undergoing diversifying selection [62].

## Results

### Nucleotide and predicted protein sequence of leptin and leptin receptor

The potential full-length transcript of *lepa* obtained from testicular transcriptome data contained 1884 bp including 480 bp long coding sequence. Surprisingly, existence of *lepb* could not be ascertained even after using respective nucleotide and protein sequence of *C*. *argus*. In case of *lepr*, the selected transcript was partial from 5’ end and comprised of 4002 bp. The remaining 838 bp was obtained following 5’ RACE. Further, a complete coding sequence of *lepr* consisting of 3447 bp was obtained using ORF finder. The ORF of leptin and leptin receptor encoded 159 and 1129 amino acids long proteins, respectively. Sequence analysis of Lepa following SignalP 4.1 predicted a signal peptide consisting of 20 amino acids. The putative full-length sequence of *lepa* and *lepr* of *C*. *punctata* were submitted to NCBI (GenBank accession number MK039679.1 and MK039680.1, respectively).

### Structural analyses

#### Physicochemical properties of Lepa and Lepr

The computed physicochemical properties of leptin and leptin receptor of spotted snakehead using human as reference are shown in Table 1. In both Lepa and Lepr of *C*. *punctata*, instability index was more than 40, aliphatic index was high and proportion of negatively charged residues was higher than the positively charged amino acids. A positive GRAVY score was evidenced for Lepa (score: 0.060) and transmembrane domain of Lepr (TMD score: 2.539) while negative score for extracellular (ECD) and intracellular (ICD) domains of the receptor (ECD: -0.381; ICD: -0.629). The sequence of leptin showed maximum percentage of leucine (15.7%) and minimum of cysteine and tryptophan (1.3% each) (S1A Fig). The leptin receptor of *C*. *punctata* was rich in serine (11.1%) while poor in histidine and methionine (2.2% each) (S1B Fig). The hydrophobicity test using ExPASyProtScale revealed maximum score for leucine at 8^th^ position in Lepa (L-8, score: 2.456; S2A Fig) and valine at 809^th^ position in Lepr (V-809, score: 3.444; S2B Fig) while minimum hydrophobicity score for glutamine in Lepa (Q-111, score: -2.222; S2A Fig) and glutamic acid (E-1080)/glutamine (Q-1081) in Lepr (score: -3.611; S2B Fig).

**Table 1:** Physicochemical properties of leptin and leptin receptor of *Channa punctata*.

Physicochemical property	*C*. *punctata* Lepa	*H*. *sapiens* LEP	*C*. *punctata* Lepr	*H*. *sapiens* LEPR
**Number of amino acids**	159	167	1148	1165
**Molecular weight (Da)**	17697.54	18640.57	128652.23	132449.78
**Total number of negatively charged residues (Asp + Glu)**	18 (11.32%)	14 (8.38%)	147 (12.8%)	118 (10.13%)
**Total number of positively charged residues (Arg + Lys)**	15 (9.43%)	11 (6.59%)	87 (7.58%)	104 (8.93%)
**Instability Index**	40.17	47.52	53.73	45.60
**Aliphatic index**	109.06	110.84	77.73	85.42
**Grand average of hydropathicity (GRAVY)**	0.060	0.131	-0.395 (overall)	-0.173 (overall)
			-0.381 (ECD)	-0.136 (ECD)
			2.539 (TMD)	2.3 (TMD)
			-0.629 (ICD)	-0.465 (ICD)

Lepa: leptin paralog a, Lepr: leptin receptor; ECD: extracellular domain, TMD: transmembrane domain, ICD: intracellular domain. Human orthologs were used as reference.

#### Secondary and tertiary structures of Lepa and Lepr

**Fig 1:**
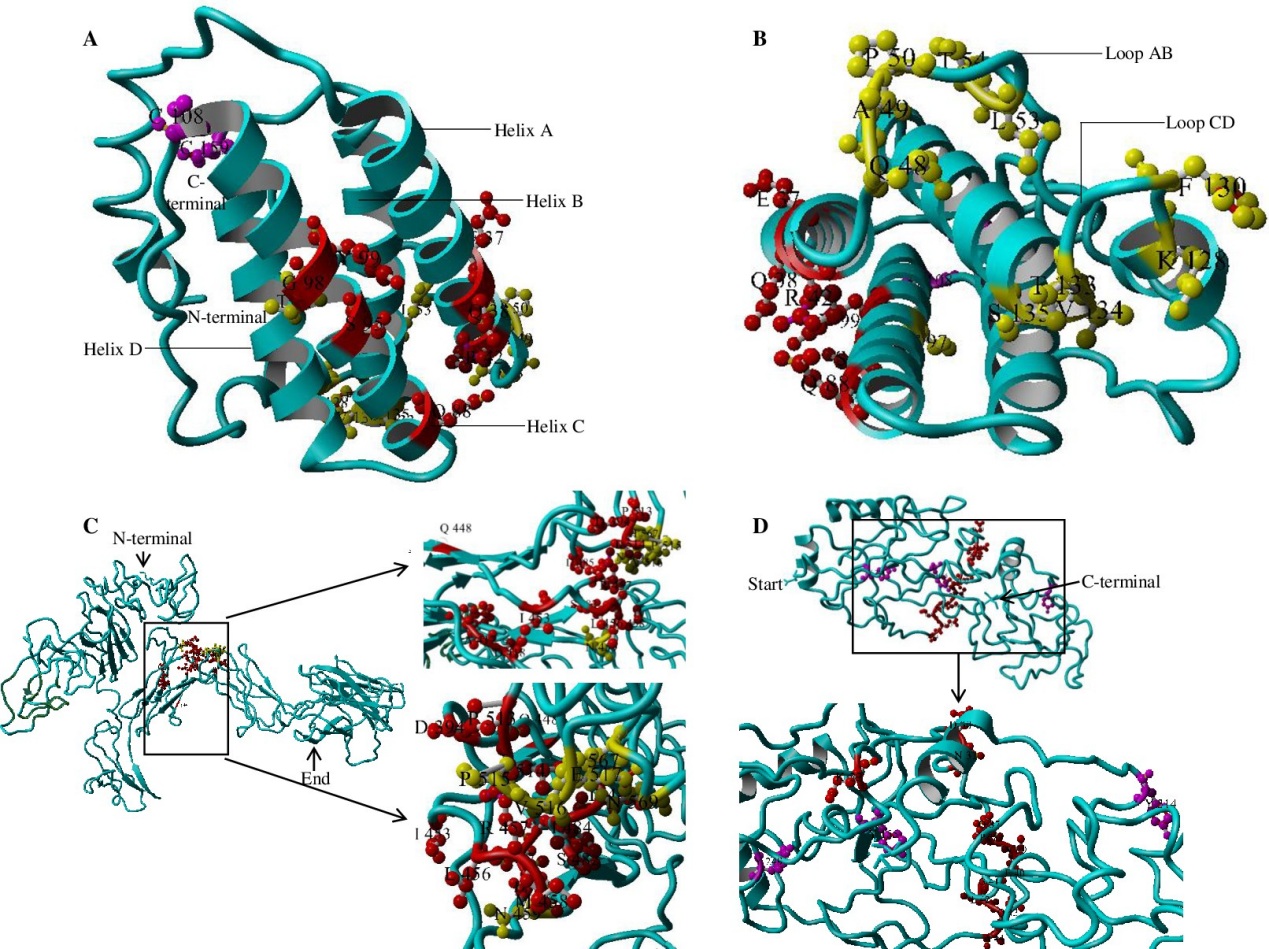
Tertiary structure of A,B leptin (Lepa) and C,D leptin receptor of *C*. *punctata*. **A, B** Using ball-and-stick model, residues of leptin involved in binding and signalling are highlighted in red and yellow colours, respectively. The cysteine residues are shown in purple. **C** In extracellular domain of leptin receptor, residues predicted essential for binding to the ligand and receptor activation/signal transduction are shown in red and yellow, respectively. **D** Represents intracellular domain of leptin receptor in which residues implicated in JAK2 activation are highlighted by red ball-and-sticks while tyrosine essential for receptor signalling by purple colour. The residues were labelled using YASARA software (version 17.12.24).

The secondary structure showed that a large proportion of leptin of *C*. *punctata* consisted of α-helices (75%) and not of β-sheets (Fig 1A and 1B). Unlike leptin, β-sheets constituted a major proportion (45%) of Lepr while a minor proportion was represented by α-helices (4%) and transmembrane region (1%) (Fig 1C and 1D). Tertiary structure of leptin of *C*. *punctata* consisted of four α-helices designated as helix A (26^th^-45^th^ amino acid), B (65^th^-78^th^), C (84^th^-104^th^) and D (134^th^-154^th^) along with long AB (46^th^-64^th^) and CD (105^th^-133^th^) loops and a short BC (79^th^-83^th^) loop (S3 Fig). The α-helices appeared to be arranged in an up-up-down-down configuration (Fig 1A and 1B). In case of leptin receptor, various domains including ECD, TMD and ICD were made up of 757, 22 and 334 amino acids, respectively (Fig 1C and 1D; S4 Fig). The Ramachandran plot for quality assessment showed good quality of leptin as most of its residues (85%) were modelled with 90% confidence (Phyre2) and placed in favourable region (S1 Table; S5A Fig). With regard to Lepr, ECD (88% residues modelled with >90% confidence) was of better quality than ICD (only 6% residues showed >90% confidence interval) of Lepr (S1 Table; S5B, S5C Fig).

### Evolutionary analyses of Lep and Lepr

#### Identification and conservation of functionally important domains/residues

A number of residues that might be involved in providing structural integrity to the hormone and responsible for binding as well as receptor activation were identified in leptin (*lepa*) of *C*. *punctata* (Table 2; Fig 1A, 1B). The residues belonging to the helical region of leptin exhibited a higher conservation as compared to loop region. Further, arginine (R-42) in helix A, glutamine (Q-88) in helix C and two cysteine residues, one (C-108) at the start of CD loop and the other (C-159) at C-terminal, were found to be conserved throughout vertebrates and marked to be essential for conferring 3-dimensional (3D) structure to the leptin. With regard to residues involved in binding to the receptor, Q-88 in helix C was conserved in leptin of all the vertebrates. However, most of the other residues including E-37, Q-38, S-95, T-97, G-98, and Y-99 showed moderate conservation among teleosts but divergence from tetrapods. Likewise, the residues (Q-48, A-49, P-50, I-55, K-128, F-130, S-135) identified crucial for receptor activation showed limited conservation among fishes and even tetrapods. A stretch of amino acids ‘LDFIP’ in AB loop of human LEP associated with downstream signalling was seen conserved in amphibians, reptiles and other mammals. In Pisces, gaps were present in homologous region of LDFIP in a few fishes including *C*. *punctata* while deviation in amino acids were found in *O*. *mykiss*, *S*. *salar*, *T*. *fulvidraco*, *D*. *rerio*, *C*. *idella* and *H*. *molitrix* (S3 Fig).

**Table 2:** Predicted effect of substitution/deletion in functionally significant sites of leptin of *Channa punctata*.

S. No.	Functionally important residue in human leptin	Residue substituted in *C*. *punctata* leptin	Conservation/ Divergence	PROVEAN Analysis (cut off = -2.5)
**Residues for binding to Lepr**				
1.	K-36	E-37	Conserved in a few teleosts	Neutral (-2.201)
2.	T-37	Q-38	Conserved in a few teleosts	Deleterious (-4.421)
3.	R-41	R-42	Conserved throughout vertebrates	NA
4.	Q-96	Q-88	Conserved throughout vertebrates	NA
5.	N-103	S-95	Conserved in a few teleosts	Deleterious (-4.146)
6.	R-105	T-97	Conserved in a few teleosts	Deleterious (-3.771)
7.	D-106	G-98	Conserved in a few teleosts	Neutral (-2.497)
8.	L-107	Y-99	Conserved in a few teleosts	Deleterious (-3.5)
**Residues for signalling of Lepr**				
9.	R-41	R-42	Conserved throughout vertebrates	NA
10.	S-50	Q-48	Conserved in a few teleosts	Neutral (-1.848)
11.	V-51	A-49	Not conserved	Neutral (-2.405)
12.	S-52	P-50	Conserved in a few teleosts	Neutral (-1.926)
13.	Q-55	L-53	Conserved in a few teleosts	Neutral (-2.141)
14.	K-56	T-54	Conserved in a few teleosts	Neutral (-2.309)
15.	L-60	-	Gap in most teleosts	Deleterious (-11.16)
16.	D-61	-	Gap in most teleosts	Deleterious (-13.64)
17.	F-62	-	Gap in most teleosts	Deleterious (-13.30)
18.	I-63	S-56	Conserved in a few teleosts	Deleterious (-5.03)
19.	E-136	K-128	Conserved in a few teleosts	Neutral (-1.742)
20.	S-138	F-130	Conserved in a few teleosts	Deleterious (-3.985)
21.	S-141	T-133	Conserved in most teleosts	Neutral (-0.805)
22.	T-142	V-134	Conserved in most teleosts	Neutral (-1.763)
23.	E-143	S-135	Conserved in a few teleosts	Deleterious (-2.697)

Gaps in leptin of *C*. *punctata* are shown by hyphen (-). Abbreviation NA represents that tool was ‘not applied’ for analysis of conserved residues.

In Lepr of *C*. *punctata*, an N-terminal domain (NTD) (27^th^-107^th^), immunoglobulin-like fold (IGD) (281^th^-368^th^), two cytokine receptor homology (CRH) domains (CRH I: 98^th^-296^th^, CRH II: 488^th^-686^th^) and three fibronectin type III domains (FNIII) (181^th^-261^th^, 470^th^-553^th^, 670^th^-760^th^) were identified in the ECD region using SMART and MSA tools. A pair of WSXWS motifs, one (282^th^-286^th^) towards N-terminal whilst another (574^th^-578^th^) at C-terminal of ligand binding domain (LBD; 304^th^-365^th^), and three pairs of cysteine residues (C-389—C-400; C-421—Y-480; C-441—C-451) known to be involved in disulphide bonding were found conserved throughout the vertebrates except the last cysteine residue which was substituted by tyrosine in Lepr of the fishes including *C*. *punctata*. However, the predicted LBD of *C*. *punctata* showed limited conservation with mammalian protein. In case of ICD, homology motifs box 1, 2 and 3 were predicted ranging from 819^th^-830^th^, 860^th^-875^th^ and 1128^th^-1131^th^, respectively, exhibited conservation among vertebrates. Some amino acids [FQPVEGLQA (850^th^-858^th^)] required for Janus kinase (JAK) activation showed lesser conservation while other residues [KCSWAKG (831^th^-837^th^) and DNFDHL (844^th^-849^th^)] implicated in JAK binding and activation were seen highly conserved. Also, residues including L-849, F-850, Y-969, Y-1063 and Y-1128 involved in Lepr signalling were conserved across vertebrates (S4 Fig). The residues in Lepr of *C*. *punctata* predicted to be involved in binding to Lep, receptor activation, signal transduction and JAK2 activation are enlisted in Table 3 and highlighted in Fig 1C and 1D.

**Table 3:** Possible effect of substitution/deletion at functionally significant sites of leptin receptor of *Channa punctata*.

S. No.	Functionally important residue in human leptin	Residue substituted in *C*. *punctata* leptin	Conservation/ Divergence	PROVEAN Analysis (cut off = -2.5)
**Residues proposed in binding to leptin**				
1.	Y-441	D-394	Conserved in a few teleosts	Neutral (-1.046)
2.	R-468	W-416	Conserved in a few teleosts	Neutral (-1.352)
3.	S-469	A-417	Conserved in a few teleosts	Neutral (-1.138)
4.	S-470	D-418	Conserved in a few teleosts	Neutral (-0.261)
5.	F-500	I-453	Conserved in a few teleosts	Neutral (-1.218)
6.	I-503	L-456	Conserved in most teleosts	Neutral (-0.550)
7.	F-504	R-457	Conserved in most teleosts	Neutral (-1.644)
8.	L-505	M-458	Conserved in a few teleosts	Neutral (-0.665)
9.	L-506	-	Gap in a few teleosts	Deleterious (-5.029)
10.	L-530	S-482	Conserved in a few teleosts	Neutral (0.183)
11.	D-532	I-484	Conserved in a few teleosts	Neutral (-0.026)
12.	S-533	D-485	Conserved in most teleosts	Neutral (1.948)
13.	V-562	P-513	Conserved in a few teleosts	Neutral (-1.524)
14.	F-563	L-514	Conserved in teleosts	Neutral (-0.034)
**Residues for receptor activation**				
15.	S-507	N-459	Conserved in a few teleosts	Neutral (-0.498)
16.	D-617	N-569	Conserved in most teleosts	Neutral (-1.646)
**Residues for signal transduction**				
17.	E-565	V-516	Conserved in a few teleosts	Neutral (0.123)
18.	N-566	E-517	Conserved in a few teleosts	Neutral (-1.494)
19.	N-567	G-518	Conserved in most teleosts	Neutral (-0.872)
20.	K-594	-	Gap in all teleosts	Deleterious (-5.097)
21.	R-615	H-567	Conserved in most teleosts	Neutral (-0.936)
**Residues required for JAK2 activation**				
22.	Q-885	K-836	Conserved in most teleosts	Neutral (-0.943)
23.	E-893	D-844	Conserved in most teleosts	Neutral (0.132)
24.	T-894	N-845	Limited conservation	Deleterious (-2.673)
25.	E-896	D-847	Conserved in a few teleosts	Neutral (-0.799)
26.	I-900	Q-851	Conserved in a few teleosts	Neutral (-0.780)
27.	K-901	P-852	Conserved in a few teleosts	Neutral (-0.073)
28.	H-902	V-853	Limited conservation	Deleterious (-3.679)
29.	T-903	E-854	Conserved in most teleosts	Neutral (0.307)
30.	A-904	G-855	Conserved in most teleosts	Neutral (0.140)
31.	S-905	L-856	Conserved in most teleosts	Neutral (-1.786)
32.	V-906	Q-857	Limited conservation	Neutral (-1.518)
33.	T-907	-	Gap in all teleosts	Deleterious (-3.755)
34.	C-908	A-858	Conserved in most teleosts	Neutral (0.367)
**Residues in homology box 1**				
35.	K-868	R-819	Conserved in a few teleosts	Neutral (-1.758)
36.	K-869	S-820	Unique	Neutral (-2.448)
37.	F-871	V-822	Conserved in a few teleosts	Neutral (-1.356)
38.	E-873	K-824	Conserved in a few teleosts	Neutral (-0.123)
39.	K-879	N-830	Conserved in a few teleosts	Neutral (-0.604)
**Residues in homology box 2**				
40.	E-914	P-864	Conserved in a few teleosts	Neutral (-1.157)
41.	P-915	S-865	Conserved in most teleosts	Neutral (-1.793)
42.	T-917	N-867	Conserved in a few teleosts	Neutral (-0.457)
43.	E-920	K-870	Conserved in a few teleosts	Neutral (-1.150)
44.	D-921	V-871	Conserved in most teleosts	Neutral (-1.297)
45.	S-923	V-873	Unique	Neutral (0.454)
46.	D-925	E-875	Conserved in a few teleosts	Neutral (-0.301)
**Residues in homology box 3**				
47.	M-1142	L-1129	Conserved in most teleosts	Neutral (-0.535)

Gaps in leptin receptor of *C*. *punctata* are shown by hyphen (-).

#### Substitutions/Deletions of residues predicting functional modifications

**Fig 2:**
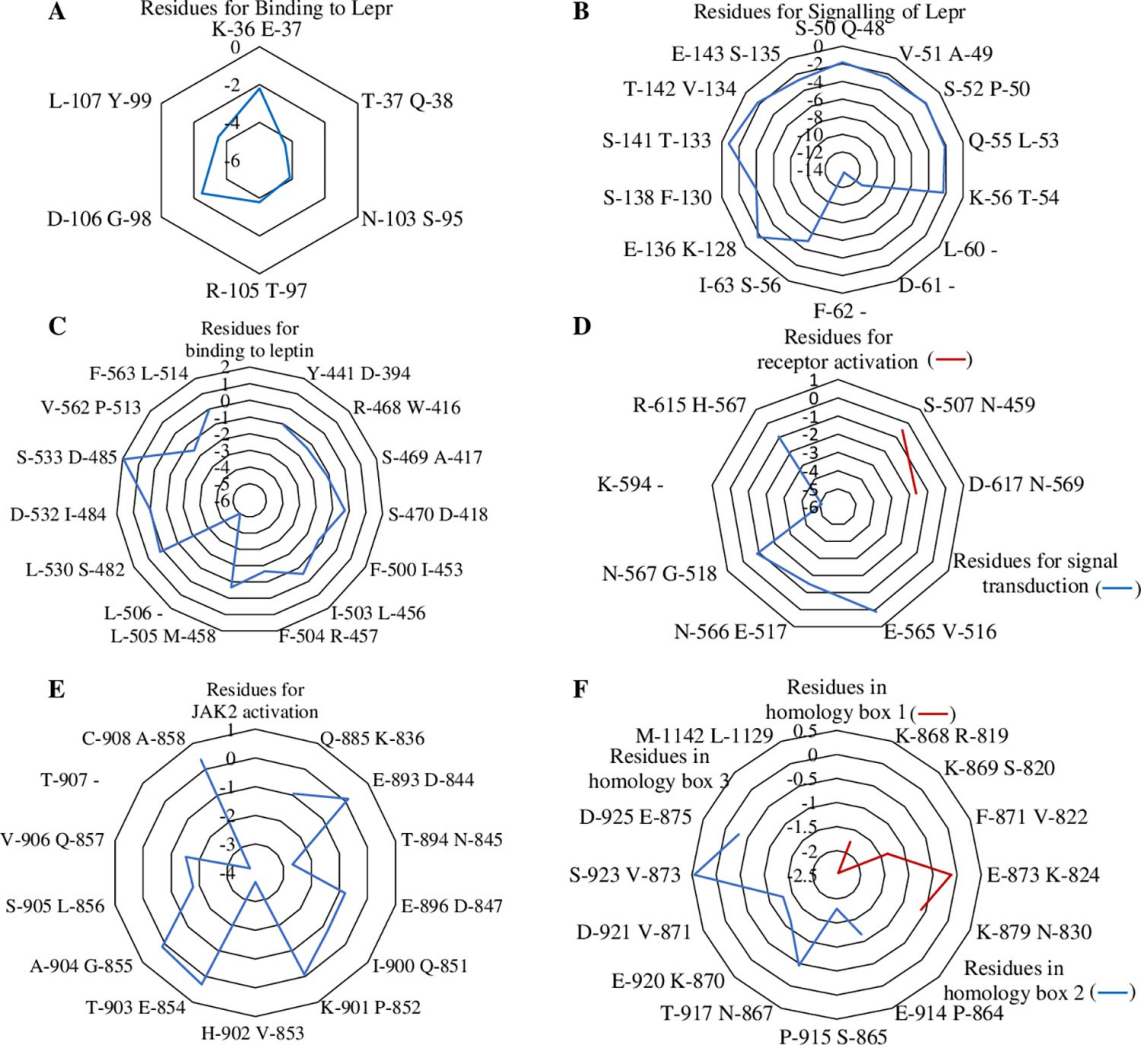
Polar curves showing PROVEAN score of the amino acids exhibiting substitution/deletion at the significant sites of (A,B) leptin and (C-F) leptin receptor. The position of amino acid increases clockwise and effect of substitutions/deletions at functionally important sites is identified at a threshold score of -2.5 considering human LEP and LEPR as reference. The PROVEAN score ≤ -2.5 implies that the protein variant would have “deleterious” effect while a score > -2.5 predicts that the variant would have a “neutral” effect.

As compared to residues of human LEP essential for receptor binding and activation, substitutions/deletions of most of the residues in Lepa of *C*. *punctata* observed following MSA when analysed employing Protein Variation Effect Analyzer (PROVEAN) tool (Table 2; Fig 2A and 2B) highlighted major variations in binding specificity between piscine and mammalian leptin. Unlike leptin, majority of the variations in functionally relevant residues and domains of Lepr in *C*. *punctata* were found to be neutral (Table 3; Fig 2C–2F).

#### Posttranslational modifications

In leptin of *C*. *punctata*, S-126 showed the possible site of phosphorylation while an intramolecular disulphide bond formation was predicted between C-108 and C-159. In leptin receptor, a number of posttranslational modifications were predicted and majority of them were localized in the ECD region (Table 4).

**Table 4:** Predicted post-translational modifications in leptin and leptin receptor of *Channa punctata*.

S. No.	Post-translational modification	Residue/position	
**Leptin**			
1.	Phosphorylation	126	
2.	Disulphide bond	108, 159	
**Leptin receptor**		**Extracellular Domain**	**Intracellular Domain**
1.	N-linked glycosylation	106, 205, 240, 266, 284, 323, 569, 608, 680, 701, 714	867, 1001, 1005
2.	Casein kinase-2 phosphorylation	180, 224, 251, 274, 391, 433, 482, 551, 710, 729, 748	904, 908, 995, 1039, 1047, 1075, 1086
3.	PKC phosphorylation	46, 75, 78, 123, 230, 251, 353, 401, 530, 694, 750	1022

#### Analogy of primary sequence of Lepa and Lepr

The primary sequence of leptin of *C*. *punctata* showed maximum similarity with its homolog of *C*. *striata* (91.19%) of the same order Anabantiformes. Further, high percentage (>75%) similarity was evidenced with the fishes belonging to order Perciformes (*E*. *coioides*, *D*. *labrax)*, Cichliformes (*O*. *niloticus*, *O*. *mossambicus*), Scombriformes (*S*. *japonicus*), Pleuronectiformes (*P*. *olivaceus*, *S*. *maximus*), moderate (40–75%) with Beloniformes (*O*. *latipes*), Tetraodontiformes (*T*. *rubripes*), Syngnathiformes (*H*. *erectus*), *C*. *semilaevis*, a Pleuronectiform fish and low (<30%) with fishes of order Cypriniformes (*C*. *idella*, *D*. *rerio*, *H*. *molitrix*), Siluriformes (*T*. *fulvidraco*) and Salmoniformes (*O*. *mykiss*, *S*. *salar*). When compared with leptin of tetrapods, percentage similarity was seen very poor ranging between 18–26% (S6A Fig). With regard to receptor, maximum similarity of primary sequence of Lepr of *C*. *punctata* was evidenced with that of *C*. *striata* (81.28%), followed by Scombriformes (*S*. *japonicas*), Pleuronectiformes (*P*. *olivaceus*, *S*. *maximus*) and Perciformes (*D*. *labrax*) (75–80%), and low with rest of the teleosts (40–75%). Similar to leptin, a poor percentage similarity was observed between Lepr of *C*. *punctata* and that of tetrapods (<30%) (S6B Fig).

#### Phylogenetic analysis

**Fig 3:**
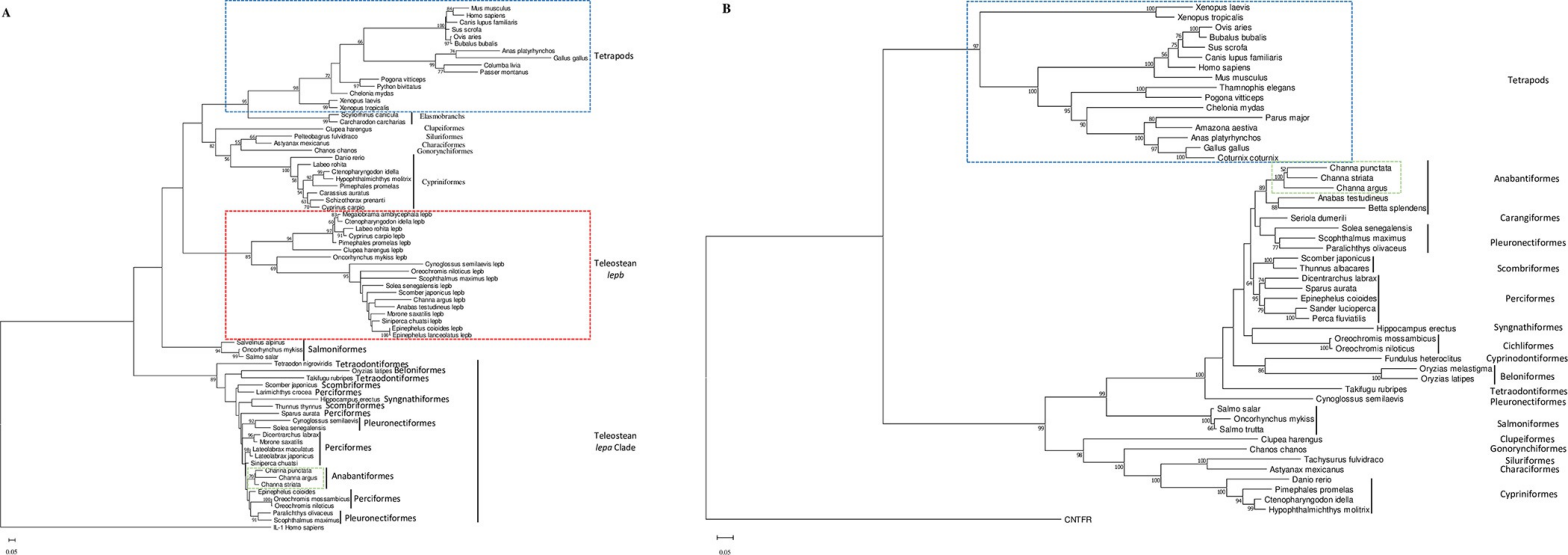
Molecular phylogenetic tree of A leptin and B leptin receptor. The tree was constructed using Maximum Likelihood method based on Jones-Taylor-Thornton substitution model. Numbers over nodes represent confidence interval obtained by bootstrapping (1000 replicates). The scale along the branch length represents number of substitutions per site (MEGA 11). The order of fishes is mentioned besides the name of organism. The cluster of tetrapods is boxed with blue color while that of *C*. *punctata* with green color. Red box is used to mark Lepb sequence. There were 74 and 52 protein sequences in Lep and Lepr phylogenetic trees, respectively.

The phylogenetic tree of leptin showed separate clusters for teleosts and tetrapods (Fig 3A). Within teleostean clade, Lepa of *C*. *punctata* cladded with that of *C*. *striata* and *C*. *argus* belonging to the same order, Anabantiformes. Interestingly, *lepa* of elasmobranchs (*Scyliorhinus canicula*, *Carcharodon carcharias*), as well as teleosts including Cypriniformes (*C*. *idella*, *D*. *rerio*, *H*. *molitrix*), Characiformes (*Astyanax mexicanus*), Gonorhynchus (*Chanos chanos*), Clupeiformes (*Clupea harengus*) and Siluriformes (*P*. *fulvidraco*) clustered with that of tetrapods. A similar pattern of cladding was noticed with *lepb*. In case of leptin receptor, two separate clades, one of higher vertebrates and other of fishes, were observed in the phylogenetic tree wherein Lepr of *C*. *punctata* clustered with that of *C*. *striata* and *C*. *argus* (Fig 3B).

#### Selection pressure analysis on teleostean leptin and leptin receptor

**Fig 4:**
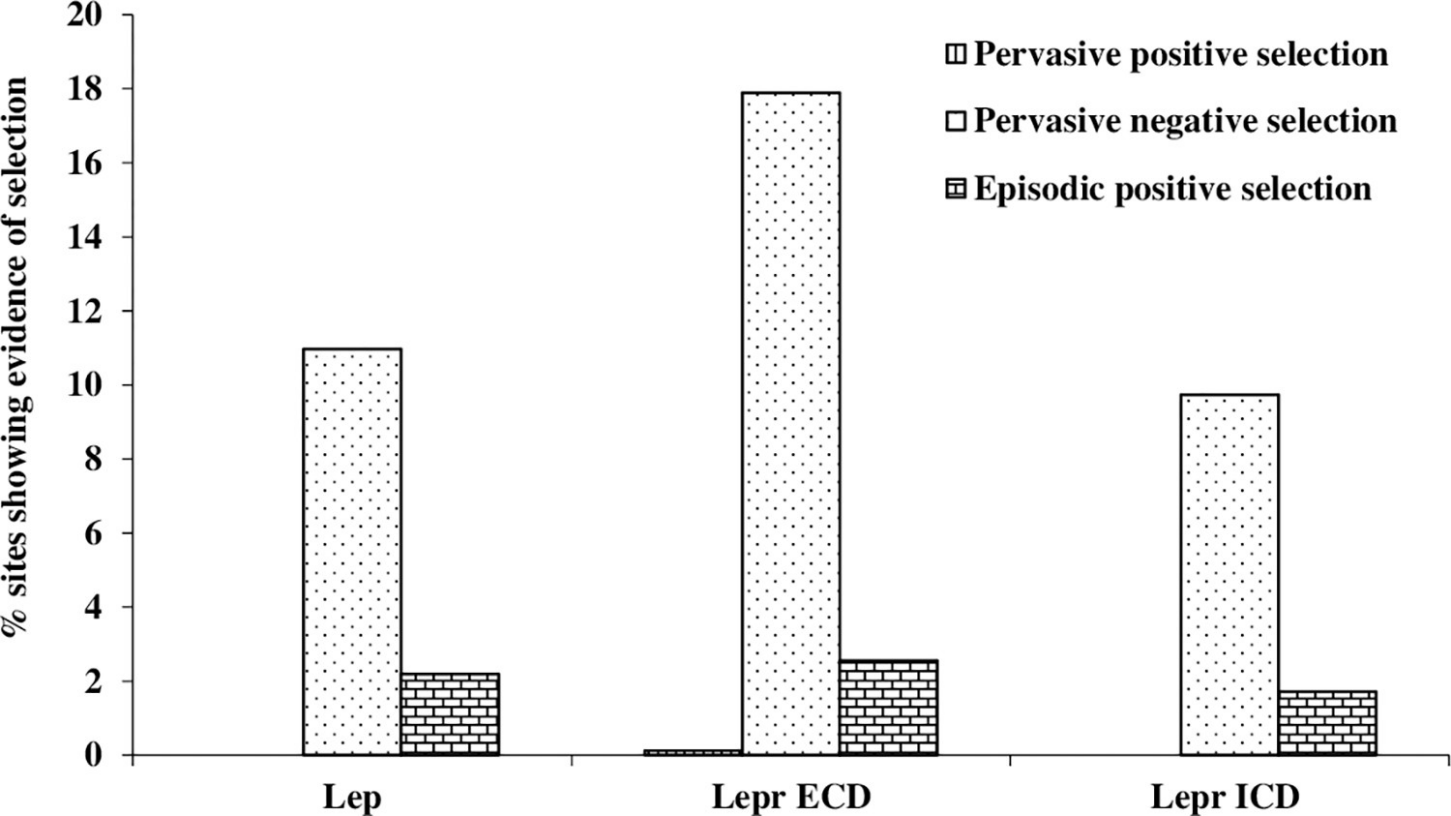
Bar diagram showing percent sites in leptin (*lepa*) and leptin receptor evidenced diversifying or purifying selection. Lep: leptin; Extracellular domain of leptin receptor (Lepr): ECD; Intracellular domain of leptin receptor: ICD.

**Fig 5:**
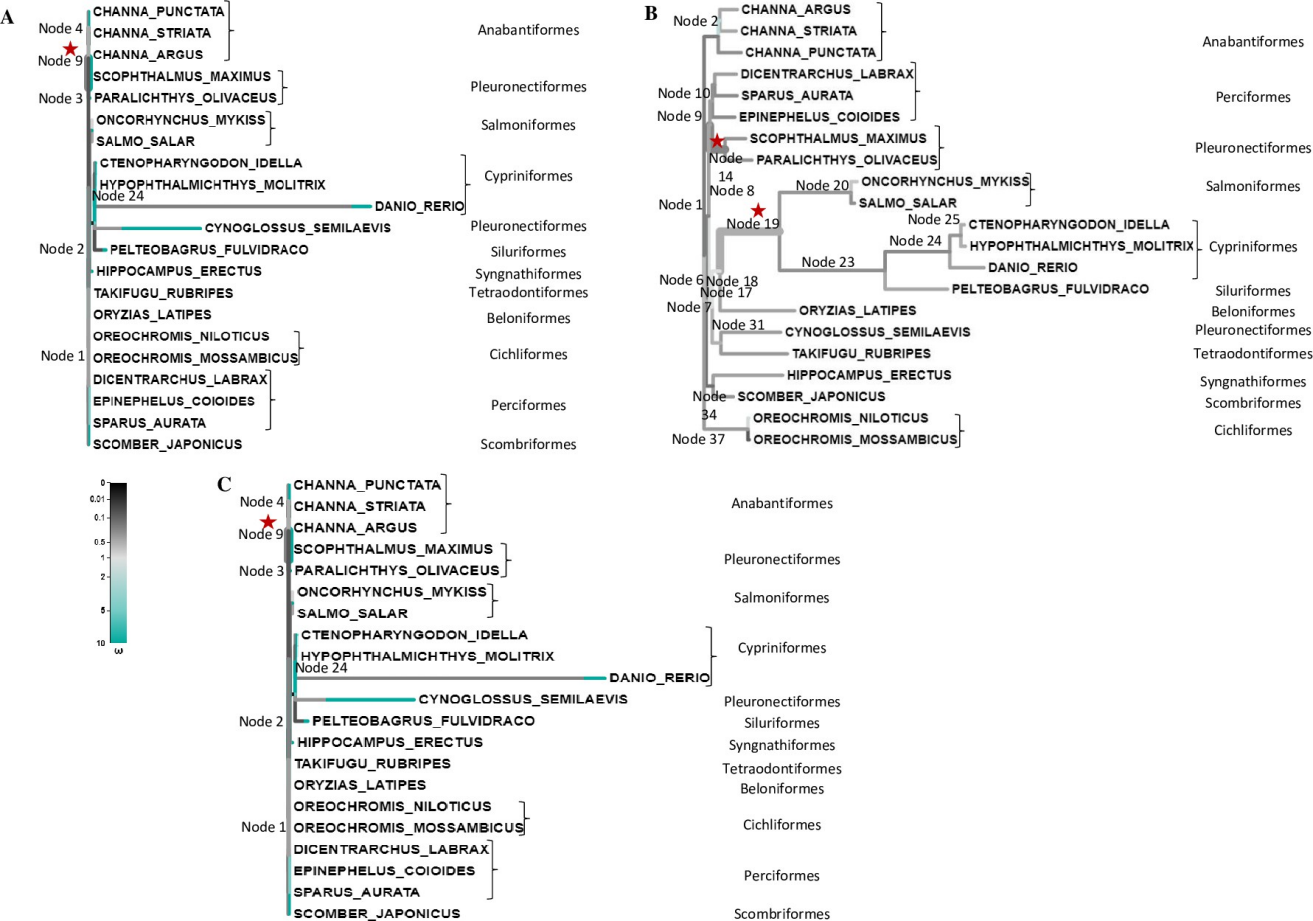
Adaptive branch-site random effects likelihood analysis of leptin (*lepa*) and leptin receptor. Branches undergoing diversifying selection (*p ≤* 0.05) in **A** leptin and **B,C** different domains of leptin receptor (**B**: extracellular domain; **C**: intracellular domain) using 21 teleosts including *C*. *punctata* are highlighted with star. Strength of selection pressure (negative/neutral/positive) is indicated in different colours (Black: negative selection, i.e. ω = 0; grey: neutral selection, i.e. ω = 1; blue: positive selection, i.e. ω > 1). The thickness of the branches is an indicator of the proportion of sites undergoing episodic selection.

A number of residues were detected in *lepa* of teleosts that were found to experience selection constraint (Fig 4). Site-specific selection analysis detected 25 sites that had experienced purifying selection while no site was detected for diversifying selection (FUBAR, posterior probability of 0.99, S7A Fig). Further, 5 sites were spotted (55^th^, 103^th^, 105^th^, 134^th^, 159^th^) that withstand episodic diversifying selection pressure (MEME at *p* = 0.05, S7A Fig). Regarding branch-specific selection pressure analysis, a total of 38 branches were formally tested with a single branch comprising of *S*. *auratus* under the evidence of episodic diversifying selection in leptin phylogeny (aBSREL, *p* ≤ 0.05; Fig 5A).

In Lepr of teleosts, selection pressure analysed separately for ECD and ICD depicted a number of amino acids under site-specific selection constraint (Fig 4). Analysing ECD region, only one site (114^th^) was found exhibiting diversifying selection pressure while 161 sites in its different domains showed purifying selection pressure (FUBAR, S7B Fig). The evidence of episodic positive selection was detected at 23 positions and majority of these were localized in NTD, FNIII and CRHI domains (MEME, S7B Fig). The aBSREL analysis (*p* ≤ 0.05) of ECD of Lepr predicted nodes 14 and 19 under episodic diversifying selection (Fig 5B). In the ICD, 51 sites including residues crucial for signal transduction, JAK binding and activation, and homology motifs box 1 and 2 were detected to undergo site-specific purifying selection while no site showed diversifying selection (FUBAR, S7B Fig). Nonetheless, 9 sites represented the evidence of episodic positive selection (MEME, S7B Fig). Only one (node 9) out of 38 branches was identified to undergo diversifying selection by aBSREL analysis (*p* = 0.0442; Fig 5C).

## Discussion

In the current study, unearthing of leptin transcript of *C*. *punctata* affirm the existence of *lepa* across the species of Anabantiformes. In addition, we isolated cDNA encoding *lepr* to develop an insight on physicochemical properties, structural/functional and evolutionary aspects of ligand and its receptor in *C*. *punctata*. To our surprise, physicochemical properties of Lepa and Lepr have not been investigated so far despite characterization of leptin system in all classes of vertebrates. In the present study, leptin system of *C*. *punctata* studied in parallel with human LEP and LEPR exhibited similarity in physicochemical properties of their respective proteins. The instability index, a measure to estimate the stability of a protein under ex vivo condition [63], greater than 40 for leptin as well as leptin receptor of both *C*. *punctata* and human indicates their lesser stability in test tube. Further, we hypothesize hydrophobic nature of leptin in vertebrates based on its GRAVY score in *C*. *punctata* and human as this score has been considered an indicator of solubility of proteins [34, 36, 64]. This assumption gets support from a recent study in *O*. *mykiss* in which presence of leptin binding proteins (LBPs) has been demonstrated along with a proposition of their role in aiding transport of leptin across blood-brain barrier [20]. Moreover, studies in mammals report bound form of leptin either with LBPs [65] or soluble leptin receptor [66]. In case of Lepr, a negative GRAVY score for extra- and intra-cellular domains in *C*. *punctata* and human suggests hydrophilic nature of these domains while transmembrane domain seems to be hydrophobic due to its positive score. Another physicochemical property, aliphatic index was recorded high for Lepa and Lepr of *C*. *punctata* and human indicating that these proteins are thermostable. This proposition is based on evidence that aliphatic index gauge the relative volume of a protein occupied by aliphatic side chains (alanine, valine, isoleucine and leucine) and indicates thermostability [35].

Despite having low primary sequence similarity, the higher order structure of leptin of *C*. *punctata* is comparable to that of several fishes [3, 67, 68] and tetrapods [6, 45, 47, 50]. This could be attributed to highly conserved residues that have been reported essential for conferring three-dimensional (3D) structure to the leptin. Further, conserved adjacent arginine in helix A (R-42) and glutamine in helix C (Q-88) interact with each other and form a binding site. Besides, two cysteine residues conserved in vertebrates are reported to form a disulphide bond [50] and a unique ‘cysteine knotted’ motif [69] which enables efficient folding and thereby provides structural integrity and stability to the leptin. However, divergence in ligand residues involved in binding and receptor activation between teleosts including *C*. *punctata* and tetrapods point towards variation in binding affinity/specificity of leptin from aquatic to terrestrial vertebrates. A recent *in vitro* bioassay strengthens this assumption wherein low affinity of piscine leptin is demonstrated to human LEPR [70]. Our results on structural analysis of Lepr of *C*. *punctata* are consistent with that reported in other fishes [5]. In extracellular region, CRH/IGD/FNIII domains involved in signal transduction exhibited low conservation. Further, LBD in Lepr of *C*. *punctata* exhibited less conservation with homologous residues of human LEPR re-strengthening our proposition of variation in Lep-Lepr interaction specificity in fishes as compared to mammals. Such a variation in binding specificity of Lep with Lepr has been attributed to reduced evolutionary pressure on piscine leptin [47]. This suggests a possibility of binding of leptin with, yet unknown, multiple receptors with multiple binding confirmations. On the contrary, homology box 1, 2 and 3 and the residues in intracellular region implicated in recruitment, binding and activation of Janus kinase (JAK) and signal transducer and activator of transcription (STAT3) [5, 71, 72] were seen conserved suggesting possible likeness in downstream signalling mechanism of leptin receptor from teleosts to mammals. Further, two WSXWS motifs, characteristic of class I cytokine receptors [73], were found to be conserved. These motifs have a π-cation stack configuration that in turn assists the receptor in binding with ligand [74]. Also, WSXWS motifs are reported to aid in receptor dimerization and activation [73]. This implies that Lepr across vertebrates might be undergoing dimerization to translate its physiological actions.

Apart from molecular conservation, the present study predicted the effect of substitutions/deletions at functionally relevant residues in leptin and its receptor using *in silico* approach. The human leptin after substitution of serine at 141^th^ and threonine at 142^th^ positions by alanine (S141A/T142A) has been reported to be capable to bind LEPR but incapable to activate the receptor [46]. The substitution at homologous positions (serine by threonine at 133^th^, and threonine by valine at 134^th^ position) was also observed in leptin of *C*. *punctata* though these were predicted to be neutral. Using mutagenesis approach, however, additional residues in Lep and Lepr have been recognized that play pivotal role in interaction of ligand with receptor and its downstream signalling [43, 75]. Among these, in the current study, substitution of T-37, I-63, N-103, R-105, L-107, S-138, E-143 in LEP of human by Q-38, S-56, S-95, T-97, Y-99, F-130, S-135, respectively, of Lepa of *C*. *punctata* and T-894, H-902 in human LEPR by N-845, V-853 of snakehead Lepr or deletion of L-60, D-61, F-62 in LEP and L-505, K-594, T-907 in LEPR predicted major variations in ligand-receptor interaction. Nonetheless, majority of the substitutions/deletions in residues of homology box 1, box 2 and box 3 as well as those identified for receptor activation were found to be neutral in case of Lepr of *C*. *punctata*, thereby, re-strengthening our proposition of similar mode of receptor activation in fishes as in mammals.

With regard to posttranslational modifications, formation of disulphide bond between two cysteine residues had been first reported in human leptin [76] and thereafter predicted conserved in several vertebrates [6]. In the current study, in addition to disulphide bond formation, phosphorylation of serine at 126^th^ position was predicted in Lepa of *C*. *punctata* though no such observation has been made so far in teleosts or other vertebrates. In case of Lepr of *C*. *punctata*, extracellular domain was predicted to be heavily glycosylated and phosphorylated. Studies in mammals suggest that there is an apparent increase (approximately 60 kDa) in the molecular weight of leptin receptor due to N-glycosylation [77, 78]. Although functional relevance of these modifications is least investigated, N-linked glycosylation in Ig-like and FNIII domains has been proposed to provide thermodynamic stability to maintain tertiary structure of Lepr [79]. Also, second WSXWS motif (X = asparagine in human LEPR) towards C-terminal of CRH domain has been predicted to remain exposed to the solvent due to glycosylation of its asparagine residue at 624^th^ position [42]. A similar proposition may be made in case of Lepr of *C*. *punctata* where N-569 close to the WSXWS motif was predicted to undergo N-linked glycosylation. In addition to glycosylation, posttranslational modification leading to disulphide bonding between cysteine residues has been reported in ECD of murine LEPR. Further, this modification has been demonstrated to cause oligomerization of the receptor as deletion of domains encompassing these residues led to the formation of monomers that otherwise formed ligand-independent oligomers [49]. Among cysteine residues, mutation in C-751 is reported to have no effect on binding of leptin or downstream signalling. However, in STAT-dependent signalling, C-672 has been proven to be important. Moreover, double mutation at both of these cysteine residues has resulted in an inactivation of JAK leading to impairment of signal transduction [49]. In a separate study, an additional disulphide bond formation is reported between C-606 and C-674 [74]. Although functional studies on posttranslational modifications have not been carried out in teleosts cysteine residues identified in Lepr of *C*. *punctata* at homologous positions in the present study point towards structural and functional significance of disulphide bonding in fishes also. As compared to ECD, fewer posttranslational modifications were observed in intracellular domain of Lepr of *C*. *punctata* wherein asparagine (N-867) undergoing glycosylation were localized in homology box 2. Apart from glycosylation, highly conserved tyrosine kinase phosphorylation in Lepr of *C*. *punctata* was predicted at Y-969, Y-1063 and Y-1128 which may be involved in downstream signalling through STAT molecules.

The phylogenetic trees constructed for leptin and leptin receptor in the present study exhibited different cluster for teleosts and tetrapods as shown in other fishes [3, 16, 20, 67, 80–82]. Further, both leptin and leptin receptor of *C*. *punctata* clustered close to *C*. *striata* and *C*. *argus* in the teleostean clade. Regarding assessment of selection pressure on teleostean leptin gene, a single report is available where purifying selection and not the diversifying selection have been observed [25]. They have suggested that poikilothermy might be keeping teleostean leptin gene under constant negative selection pressure as fishes do not adapt to alteration in surrounding temperature. However, no effort has been made to explore the effect of selection pressure on teleostean leptin receptor gene. The analysis of selection pressure on *lepa* and *lepr* of teleosts in the current study showed evidence of purifying as well as diversifying selection though negative selection pressure was more pronounced, implying that these genes adhere to maintain structure and function in fishes. Unlike teleosts, a considerable number of studies report diversifying selection pressure on leptin gene in mammals including seals [21, 23], whales [23], pikas [22] and bats [83]. In these species, positive selection pressure has been suggested to help in adapting to diverse environmental conditions [23]. In case of leptin receptor, however, no evidence of positive selection is reported in mammals [23, 26]. It is worthwhile to mention that pervasive as well as episodic diversifying selection pressure was observed in current study on both ECD and ICD of teleostean leptin receptor. Furthermore, marginally higher evidence of diversifying selection on ECD than ICD points towards co-evolution of leptin and its receptor in fishes.

## Conclusion

The sequence-to-structure prediction and homology of higher order structures of leptin and its receptor of *C*. *punctata* with that of human provided an explanation to resemblance in secondary and tertiary structures of respective proteins despite poor similarity in their primary sequences among vertebrates. Most of the key residues of Lepa and Lepr involved in conferring structural integrity and transducing action were seen to be conserved. This assumption is reinforced by mutational analysis wherein majority of substitutions were predicted to be neutral. Nevertheless, residues of Lepa predicted in receptor binding varied from fish to mammals indicating that Lep and Lepr binding affinity is vertebrate class-specific. The posttranslational modifications predicted in leptin and its receptor in the present study point towards their contribution in maintaining integrity of tertiary structure of both ligand and its receptor. Also, heavily glycosylated extracellular domain of the receptor in *C*. *punctata* forecasts its thermodynamic stability. The current study speculates existence of leptin binding proteins in *C*. *punctata* based on hydrophobic nature of leptin due to its positive grand average of hydropathy score. In addition to structural and functional predictions, evolutionary analysis depicted a closeness of leptin system of fishes belonging to order Anabantiformes with that of Pleuronectiformes, Scombriformes and Perciformes. Further, present study paves the way to understand how selection pressure on genes encoding leptin and its receptor had shaped their evolution in Pisces, the most diverse class of vertebrates. Considerably higher evidence of purifying selection on *lepa* and *lepr* points towards a stringency acting on these genes to adhere to their structure and concomitantly, function in fishes. Nevertheless, incidence of both pervasive and episodic diversifying selection in leptin and extra- as well as intra-cellular domains of leptin receptor provides ground to co-evolution of ligand and receptor in teleosts.

## Supporting information

S1 Fig Bar diagram showing percentage of amino acids present in A Lepa and B Lepr of *Channa punctata*. Lepa: leptin paralog a; Lepr: leptin receptor. Human LEP and LEPR were considered as reference.
(PDF)

S2 Fig Hydropathy plots of **A** leptin and **B** leptin receptor of *Channa punctata*. In parallel, human **C** LEP and **D** LEPR were also run on ExPASy ProtScale tool.
(PDF)

S3 Fig Multiple sequence alignment of leptin (Lepa) of *Channa punctata* and its orthologs in other vertebrates. Twenty-five organisms including 19 fishes and representatives from each vertebrate class were used. Based on human LEP, helices (A, B, C, D) and loops (AB, BC, CD) are marked in leptin sequence. The functionally important residues are denoted by different colours [Signal peptide in grey color; conserved R-42 in helix A, Q-88 in helix C, C-108 in CD loop and C-159 at C-terminal conferring 3D structural integrity to leptin in yellow; E-37, Q-38, R-42, Q-88, S-95, T-97, G-98, Y-99 implicated in binding to the receptor and showing limited conservation, except R-42 and Q-88 in red color which are conserved throughout vertebrates; Q-48, A-49, P-50, L-53, T-54, I-55, L-60, D-61, F-62, I-63, P-64, K-128, F-130, T-133, V-134, S-135 suggested in Lepr signalling but showing less conservation among tetrapods and teleosts in purple color]. In multi-sequences alignment, asterisk ‘*’, colon ‘:’ and period ‘.’ indicate a site with perfect alignment, a site belonging to a group with strong similarity and a site belonging to a group with weak similarity, respectively (https://www.ddbj.nig.ac.jp/faq/en/explain-three-symbols-e.html). Solid triangle represents predicted site of phosphorylation.
(PDF)

S4 Fig Multiple sequence alignment of leptin receptor. Twenty-five organisms, 19 fishes including *C*. *punctata* and representatives from each vertebrate class, were used. The extracellular domain (ECD), transmembrane domain (TMD) and intracellular domain (ICD) are underlined in orange, blue and red, respectively. The structural/functional domains are highlighted with different colors [N-terminal domain (NTD) in yellow; residues of immunoglobulin-like domain (IGD) in blue bold; ligand binding domain (LBD) in cyan; residues implicated in binding to the ligand, receptor activation, signal transduction and JAK2 activation are highlighted in red, yellow, green and purple, respectively. The conserved tyrosine Y-969, Y-1063, Y-1128 essential for signalling via SH2 containing tyrosine phosphatase 2, STAT5 and STAT3, respectively are also shown in purple]. Three fibronectin III domains (FNIII) are enclosed within black square brackets and two cytokine receptor homology (CRH) domains in curly brackets. The two conserved WSXWS motifs are enclosed in blue solid box while three conserved homology motifs box 1, 2 and 3 in black solid boxes. The residues involved in JAK2 binding are enclosed in green solid box. Three disulphide bonds formed between conserved cysteine residues (C-389- -—C-400; C-441- -—C-451; C-421- -—C-481) are highlighted in maroon solid boxes and joined by S-S bond. In multi-sequences alignment, asterisk ‘*’, colon ‘:’ and period ‘.’ indicate a site with perfect alignment, a site belonging to a group with strong similarity and a site belonging to a group with weak similarity, respectively (https://www.ddbj.nig.ac.jp/faq/en/explain-three-symbols-e.html). Solid triangles and squares represent predicted sites undergoing phosphorylation and glycosylation, respectively.
(PDF)

S5 Fig Quality assessment of tertiary structures of leptin and leptin receptor of *Channa punctata*. Ramachandran plot for **A** leptin a, **B** extracellular domain of leptin receptor and **C** intracellular domain of leptin receptor. Plot areas A, B and L represent the residues in the most favoured region; a, b, l, p represent the residues in additional allowed region; ~a, ~b, ~l, ~p represent the residues in generously allowed region. Glycine is represented by triangles.
(PDF)

S6 Fig Analogy of leptin and leptin receptor sequence of *Channa punctata* with respective sequences in other fishes. Heat map showing percentage similarity of primary sequence of **A** leptin (Lepa) and **B** leptin receptor (Lepr) of *C*. *punctata* with that of fishes belonging to different orders.
(TIF)

S7 Fig Sites in A leptin and B extracellular as well as C intracellular domains of leptin receptor of *Channa punctata* under selection pressure constraint. Multiple sequence alignment of leptin (*lepa*) and leptin receptor of *C*. *punctata* with respective homologs in other teleosts highlighting the sites on which selection pressure acted. Color key: yellow box indicates positively selected sites by FUBAR; blue box shows negatively selected sites by FUBAR and green box shows evidence of episodic diversifying selection detected by MEME.
(PDF)

S1 Table Quality assessment of tertiary structure of leptin and leptin receptor of *C*. *punctata*. (DOCX)

S2 Table NCBI accession number of protein and nucleotide sequences of leptin and its receptor of vertebrates. (DOCX)

## Abbreviations

*lep*: leptin
*lepa*: leptin paralog a
*lepb*: leptin paralog b
*lepr*: leptin receptor
ECD: extracellular domain
ICD: intracellular domain
TMD: transmembrane domain
RACE: rapid amplification of cDNA ends
ORF: Open Reading Frame
cds: coding sequence
bp: base pair
ExPASy: Expert Protein Analysis System
NTD: N-terminal domain
IGD: immunoglobulin-like fold
JAK: Janus kinase
STAT: signal transducer and activator of transcription
CRH: cytokine receptor homology domain
FNIII: fibronectin type III domain
LBD: ligand binding domain
BLASTn: Basic Local Alignment Search Tool
NCBI: National Centre for Biotechnology Information
SMART: simple modular architecture research tool
Phyre2: Protein Homology/AnalogY Recognition Engine
JAK: Janus kinase
PROVEAN: Protein Variation Effect Analyzer
FUBAR: fast, unconstrained Bayesian approximation
MEME: mixed effects model of evolution
aBSREL: adaptive Branch-Site Random Effects Likelihood
GRAVY: Grand Average Of Hydropathicity
