# Supplementary figures and images for: In silico analyses of leptin and leptin receptor of spotted snakehead Channa punctata
Source: PLoS One. 2022 Jul 7;17(7):e0270881. doi: 10.1371/journal.pone.0270881 (PMC9262212; doi:10.1371/journal.pone.0270881)

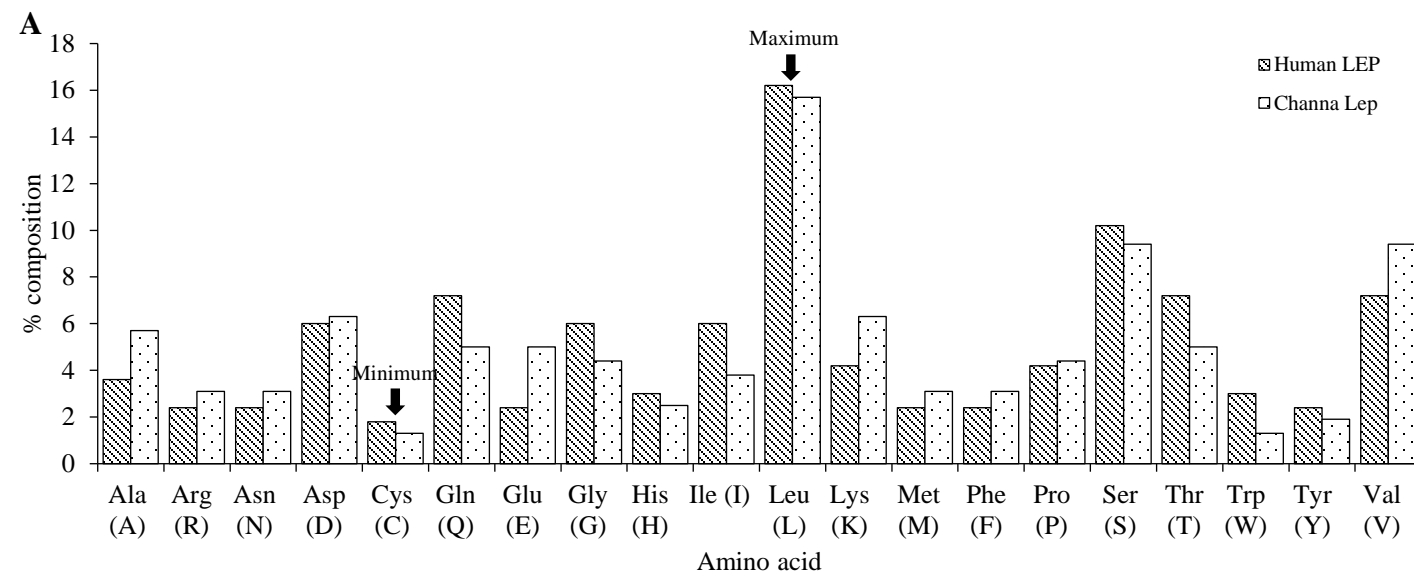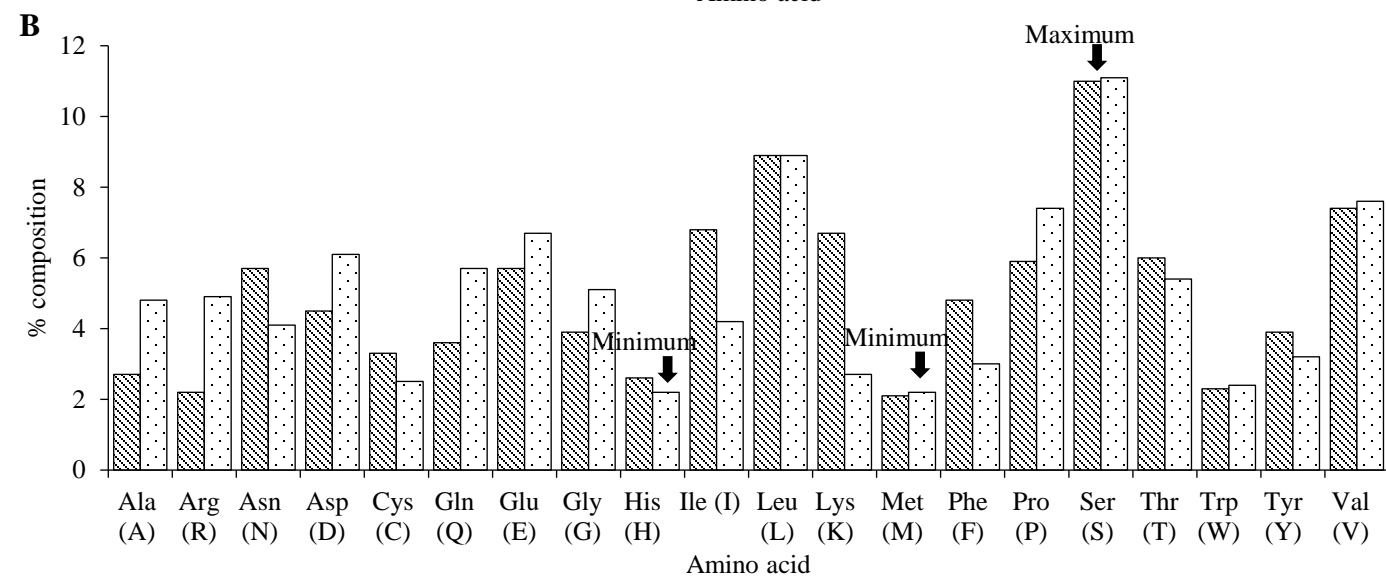

**S1 Fig**

Supplement: S1 Fig — Lepa: leptin paralog a; Lepr: leptin receptor. Human LEP and LEPR were considered as reference. (PDF) [file pone.0270881.s001.pdf]

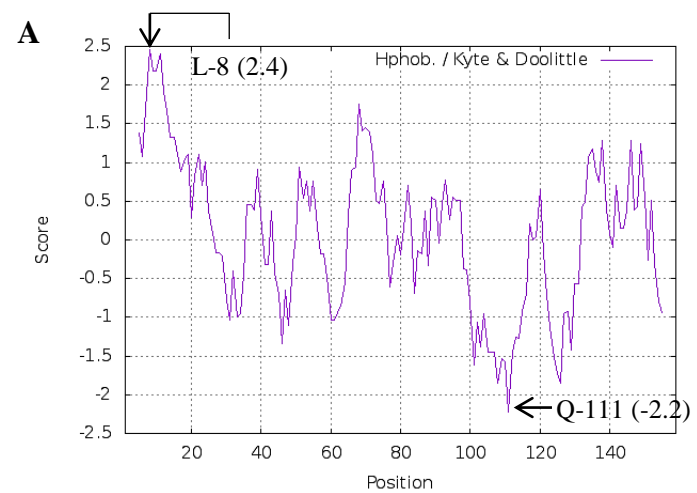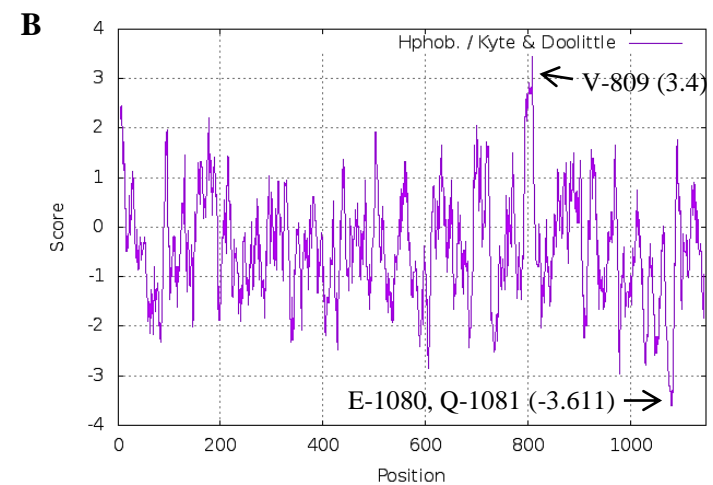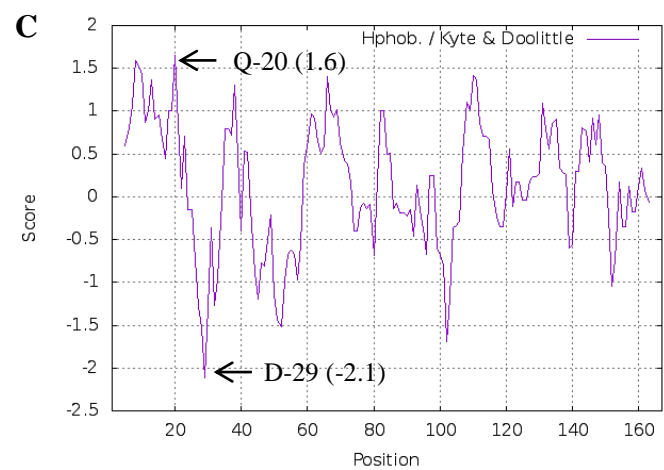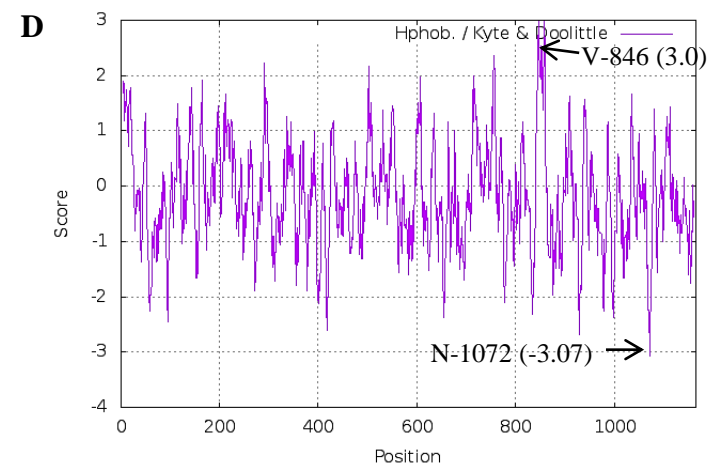

**S2 Fig**

Supplement: S2 Fig — Hydropathy plots of A leptin and B leptin receptor of Channa punctata. In parallel, human C LEP and D LEPR were also run on ExPASy ProtScale tool. (PDF) [file pone.0270881.s002.pdf]

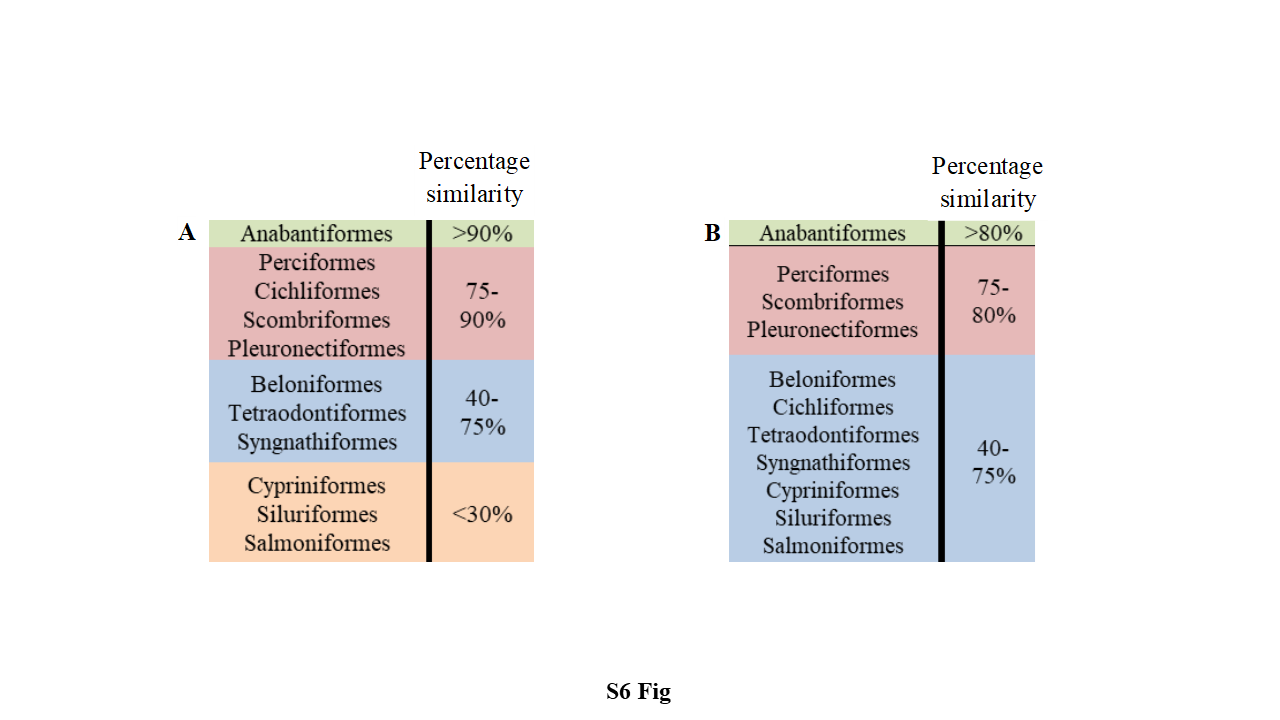

Supplement: S6 Fig — Heat map showing percentage similarity of primary sequence of A leptin (Lepa) and B leptin receptor (Lepr) of C. punctata with that of fishes belonging to different orders. (TIF) [file pone.0270881.s006.tif]
