# Supplementary material for: In silico analyses of leptin and leptin receptor of spotted snakehead Channa punctata
Source: PLoS One. 2022 Jul 7;17(7):e0270881. doi: 10.1371/journal.pone.0270881 (PMC9262212; doi:10.1371/journal.pone.0270881)
Supplement: S3 Fig — Twenty-five organisms including 19 fishes and representatives from each vertebrate class were used. Based on human LEP, helices (A, B, C, D) and loops (AB, BC, CD) are marked in leptin sequence. The functionally important residues are denoted by different colours [Signal peptide in grey color; conserved R-42 in helix A, Q-88 in helix C, C-108 in CD loop and C-159 at C-terminal conferring 3D structural integrity to leptin in yellow; E-37, Q-38, R-42, Q-88, S-95, T-97, G-98, Y-99 implicated in binding to the receptor and showing limited conservation, except R-42 and Q-88 in red color which are conserved throughout vertebrates; Q-48, A-49, P-50, L-53, T-54, I-55, L-60, D-61, F-62, I-63, P-64, K-128, F-130, T-133, V-134, S-135 suggested in Lepr signalling but showing less conservation among tetrapods and teleosts in purple color]. In multi-sequences alignment, asterisk ‘*’, colon ‘:’ and period ‘.’ indicate a site with perfect alignment, a site belonging to a group with strong similarity and a site belonging to a group with weak similarity, respectively (https://www.ddbj.nig.ac.jp/faq/en/explain-three-symbols-e.html). Solid triangle represents predicted site of phosphorylation. (PDF) [file pone.0270881.s003.pdf]

```
                                                                                     Helix A
                                                                              (26-45 amino acids)
    Oryzias_latipes               MDS-ALVLFAFLFHCLNVATAA---PVNPELQEMKSNVIDIAKELSLRLESII---QTSI   53
    Takifugu_rubripes             MDH-ILALVLALL-PLSLCVALPGALDAMDVEKMKSKVTWKAQGLVARIDKHF---PD--   53
    Hippocampus_erectus           MDCITLAILVSVSQVWGAVTAA---PMSVEVIRMKATVEGKSKQLVARLN-KI---QVPP   53
    Cynoglossus_semilaevis        -MYCSLAFLLPLLHLLSICTAA---SLPMEVVKVKSNVKQLSEQLLVRLK-NF---QFPA   52
    Scomber_japonicus             MDYSTLTLLISLSQLLSVGTAA---PLSVEVGKMKIKVKWMAEQLVAKLN-NF---QVPA   53
    Paralichthys_olivaceus        MDY-TLALLFSLLQVLSVCTAA---PLPAEVVMMKSKVKWMAEQLVVRLDKDF---NFSV   53
    Scophthalmus_maximus          MDY-TLVLLFSLLQVLSVCTAA---PLPVEVVKMKSKVKWMAEQLIIRME-DF---QFPS   52
    Dicentrarchus_labrax          MDS-TLAILFSMLQLLIVSTAA---PLPVEVVKMKSKVKWMAEQLVVRLNRDF---QVPI   53
    Oreochromis_mossambicus       MDY-GLVLLFSLFQALSMGTAA---PLPVEVVTMKSKVKWMAEQLVVRLDKDV---QVPV   53
    Oreochromis_niloticus         MDY-GLVLLFSLFQALSMGTAA---PLPVEVVTMKSKVKWMAEQLVVRLDKDV---QVPV   53
    Epinephelus_coioides          MDY-TLALLFSLLHVFSVGTAA---PLPVEVVKMKSKVKWMAEQLVVRLNKDF---QVPP   53
  ➡ Channa_punctata               MDY-TLALLFSLLHLLTAGSAA---P--VDVVKMKSKVKWMAEQLVVRLNKDF---QAPA   51
    Channa_striata                MDY-TLALLFSLLQLLSVGTAA---P--VEVVKMKSKVKWMAEQLVVRLNKDF---QVPA   51
   ┌Mus_musculus                  --MCWRPLCRFLWLWSYLSYVQ---AVPI--QKVQDDTKTLIKTIVTRINDISHTQSVSA   53
   │Homo_sapiens                  --MHWGTLCGFLWLWPYLFYVQ---AVPI--QKVQDDTKTLIKTIVTRINDISHTQSVSS   53
Tetrapods┤Anas_platyrhynchos      MWYHSAWLWGLLWLCPPPAGGR---PVRP--EKIWGDTRSLARTLSARIQLLQ---LFPL   52
   │Chelonia_mydas                MRCPSLPLCGLLWFWLPLFYSR---PVKI--DKVKADTKNLTRTIIARIQEHQ---LFPL   52
   │Xenopus_tropicalis            MQYIHLSVCGILWMLLPVCQGR---AIKA--DRVKNDAKMLARTLITRIQEHPIQFLFPS   55
   └Xenopus_laevis                MQYIHLSFWGIFWMLLPVSQGR---AIKA--DRVKNDAKLLASTLITRIQEHPIQFLFPS   55
    Oncorhynchus_mykiss           MDCSMALLLSSLLALFSVGAGA---SLSLH--VVRTKVKDLAQTMVIRI----KKLDISP   51
    Salmo_salar                   MDCSMALLLSSLLALFSVGAGA---SLSLH--VVRTKVKDLAQTMVIRI----NKLDISP   51
    Tachysurus_fulvidraco         MAVYPALFCSCVVTVLTLTNGR---ALPT--DSLKNSVKLQVENIISRIQKHKDEFPILH   55
    Danio_rerio                   -MRFPALRSTCILSMLSLIHC-----IPVHQHDRK-NVKLQAKTIIVRIREHIDGQNLLP   53
    Ctenopharyngodon_idella       -MYSPVLLYTCFLSILGMIDGR---SIPIHQDNLKNLVKLQADTIIHRIKEHNEKLKLSP   56
    Hypophthalmichthys_molitrix   -MYFPVLLYTCFLSILGLIDGR---SIPFHPESLKSL-KQQADTIIHRIKEHNEKLKLSP   55
                                              .                                 :  ::

                                      AB loop               Helix B        BC loop      Helix C
                                 (46-64 amino acids)   (65-78 amino acids)  (79-83)  (84-104 amino acids)
    Oryzias_latipes               GPKF-----S---PPSDELNGLSSIMAVLDECTNQISDN-FDEA--KKIKVDISSLMDSM   102
    Takifugu_rubripes             -RGL-----R---FDTDKVEGSTSVVASLESYNNLISDR-FGGV--SQIKTEISSLAGYL   101
    Hippocampus_erectus           GMTL-----T---PPADRLDGLSSVVTLLDGYDKLISDS-LN-V--SQVKAEISWLKSYL   101
    Cynoglossus_semilaevis        GRTL-----S---PAAGDLDRLSATVMILDGYNSLISDT-LNGV--SQIKADISCLTRHL   101
    Scomber_japonicus             GWTL-----S---QPADDVDGLSSIVVILDGYNSLISDS-LNGV--SQVKSEISSLGGYL   102
    Paralichthys_olivaceus        SLT-----------PTDDLAGASSIVTVLEGYNSLISDT-FHRV--SQVKSEISSLTGYV   99
    Scophthalmus_maximus          SLT-----------PTDDLDGASSIVTVLEGYNSLISDT-FDGV--SQVKSEISSLTGYV   98
    Dicentrarchus_labrax          GLTL-----S---PPADDLDGLSSIVTILEGYNSLISNS-LDGV--SQVKVDISSLTGFL   102
    Oreochromis_mossambicus       NWTL-----N---PPADDLDGTSSIVTVLNGYNSLIPDT-FKGV--SQIKYDISSLTGYI   102
    Oreochromis_niloticus         NWTL-----N---PPADDLDGTSSIETVLNGYNSLIPDT-FKGV--SQIKYDISSLTGYI   102
    Epinephelus_coioides          GLTL-----S---PPADILDGPSSIVTVLDGYNSLISDT-FNGV--SQVKFDISSLTGYI   102
  ➡ Channa_punctata               GLTI-----S---PPADDLDGSSSIVMVLEGYNSLISDT-FNGV--SQVKVDISSLTGYL   100
    Channa_striata                GLTL-----S---PPADNLDGSSSIVMVLEGYNSLISDT-LNGV--SQVKFDISSLTGYL   100
   ┌Mus_musculus                  KQRVTGLDFI---PGLHPILSLSKMDQTLAVYQQVLTSLPSQNV--LQIANDLENLRDLL   108
   │Homo_sapiens                  KQKVTGLDFI---PGLHPILTLSKMDQTLAVYQQILTSMPSRNV--IQISNDLENLRDLL   108
Tetrapods┤Anas_platyrhynchos      GPRVLGLEAM---PGARPPEGLGAMEQRLQLFQRVLRALPGAAAPPQILSDLENLRSLL   109
   │Chelonia_mydas                NLKINGLDFI---PGERPLESLDSMDETLQIFQRILPSLPMENVPVAQIFNDIENLRSLI   109
   │Xenopus_tropicalis            NLKINGLDFI---PDEQILESLESMDETLEVFQKIFSSLPLENV--DQMLSDMENLRSLL   110
   └Xenopus_laevis                NLKISGLDFI---PDEQLLESLEHMDETLEVFQKILSSLPMENV--DQMLSDMENLRSLL   110
    Oncorhynchus_mykiss           NLIEGMDPFLPAAAVDQHIESLPSIVETMGFYQDLLLVLDWADL--KQLVEDTSTMRGLL   109
    Salmo_salar                   NLIEGMDPFLPAAAVDQHIESLPSIMETMGFYQDLMLFLDWADL--KQLVEDTSTMRGLL   109
    Tachysurus_fulvidraco         KMILDSPELLPELQSDKPIEGLSSMVEMLNNFQRVLHSLPKGHM--SQLHSDVSTLQHYL   113
    Danio_rerio                   TLIIGDPGHYPEIPADKPIQGLGSIMETINTFHKVLQKLPNKHV--DQIRRDLSTLLGYL   111
    Ctenopharyngodon_idella       KILIGDSELYPEVPADKPIQGLGSIVDTLTTFQKILQTLPKGHV--SQLHNDMSTLLEYF   114
    Hypophthalmichthys_molitrix   KILIGDSELYPEVPADKPIQGLGSIIDTLTTFQKVLQTLPKGHV--SQLHSDVSTLLDYF   113
                                                              :      :          ::  : . :   .
```

```
                                                         CD loop                    Helix D
                                                   (105-133 amino acids)     (134-154 amino acids)

            Oryzias_latipes                 SEWSDKH-CGEQPSTQ--------AEN-QTSRRFSITESMQAVTRLKHFLLLLQNNSDQL  152
            Takifugu_rubripes               NHWREGN-CQEQQPKVW-----------PRRNIFNHTVSLEALMRVREFLKLLQKNVDLL  149
            Hippocampus_erectus             GQWKKGR-CGEAKANRTSATGGALQRL-QSQRSFVLTVGIEALVRVKDILTRMLQNMEHL  159
            Cynoglossus_semilaevis          DQWRQGH-CNKQRPK--PSVPEPLQKL-QSHKDLIDTVSFEALLRVKEILVVLLKNLDNL  157
            Scomber_japonicus               QQWKKGH-CNEQRPK--PLVSGPLQEL-QSRKEYIHTVGIEAVMRVKEFLTLLLKNLDQL  158
            Paralichthys_olivaceus          DQWRKGH-CSEQRPK--PSMPGPLQKL-QSLKDFIHIVSMEALMRVKEFLNLLLKNLDHL  155
            Scophthalmus_maximus            DQWRRGH-CSEQRPK--PSVPGPLQNL-LSRKEFVHTVTIEALMRVKELLNLLLKNLDHL  154
            Dicentrarchus_labrax            SQWRQEH-CSEQRPK--LSVPGVLQEL-QRRKTFIHTVSIEALMRVKEFLNLLLKNLNHL  158
            Oreochromis_mossambicus         HLWRQGH-CSEQRPK--PEVPGPLQEL-QSHKEFIQTVGIEALMRVKEFLNLLLKNLDQL  158
            Oreochromis_niloticus           HLWRQGH-CSEQRPK--PEVPGPLQEL-QSHKEFIQTVGIEALMRVKEFLNLLLKNLDQL  158
            Epinephelus_coioides            GQWRQGH-CTEQRPK--PSVPGPLQEL-QSRKEFIHTVSIEALMRVKEFLNLLLKNLDHL  158
          ➡ Channa_punctata                 DQWRQVH-CTEQRPK--PLVSGPLQEL-QSRKEFIHTVSIEALMRVKEFLHLLLKNLDNL  156
            Channa_striata                  NQWRQVH-CTEHRPK--PLVSGPLQEL-QSRKEFIHTVSIEALMRVKEFLYLLLKNLDHL  156
            Mus_musculus                    HLLAFSKSCSL---P-QTSGLQKPESLDGVLEASLYSTEVEALSRLQGSLQDILQQLDVS  164
            Homo_sapiens                    HVLAFSKSCHL---P-WASGLETLDSLGGVLEASGYSTEVVALSRLQGSLQDMLWQLDLS  164
Tetrapods   Anas_platyrhynchos              TALAAQLGCGP---P-PRQTEAPPPGLAELLAQAPHTVAGLAMGRLRACLDGIAARIDAA  165
            Chelonia_mydas                  QTLGSHLGCTF---H-KSSTLDALGNLTELLTTSPYTAAVVALDRLQKCLHSIIKHLDHI  165
            Xenopus_tropicalis              QLLGNIMGCTT---R-KPTQCDTQVNLTEEYAKAPYTTEKVALDRLQKSLHSIVKHLDHI  166
            Xenopus_laevis                  QSLSTIMGCTA---R-KHSQCDTQVNLTEEYAKAPYTTEKVALDRLQKSLHSIVKHLDHI  166
            Oncorhynchus_mykiss             ENWMTSR-CPARQQK-QTGEGGLSEALKDTTRKYGLSVGPVALNRLKGYLGRLLLNLDQL  167
            Salmo_salar                     ENWMISR-CPGRQQK-QTGEGRLSEALKDTTRKYGLSVGPVALNRLKGYLGRLLLNLDQL  167
            Tachysurus_fulvidraco           EDRMSSLQCTH---R-ITGTEKNLEDFPKNHSMYYITVRHVALDRLQKYIQRLNHNLEQL  169
            Danio_rerio                     E----GMDCTL---K-ESTNGKALDAFLEDSASYPFTLEYMTLNRLKQFMQKLIDNLDQL  163
            Ctenopharyngodon_idella         KDRMTFMRCTL---K-EPANGKSLDTFIEKNATHHITFGYMALDRLKQFMQKLIANLDQL  170
            Hypophthalmichthys_molitrix     KVWMTFMRCTP---K-EPANGKSLDTFIQKNATHHVTFGYMALDRLKQFMQKLIANLDQV  169
                                                    *                                :: *::  :  :  . :

            Oryzias_latipes                 EIC- 155
            Takifugu_rubripes               ERC- 152
            Hippocampus_erectus             DKC- 162
            Cynoglossus_semilaevis          EIC- 160
            Scomber_japonicus               KTC- 161
            Paralichthys_olivaceus          ETC- 158
            Scophthalmus_maximus            ESC- 157
            Dicentrarchus_labrax            KTC- 161
            Oreochromis_mossambicus         ETC- 161
            Oreochromis_niloticus           ETC- 161
            Epinephelus_coioides            ETC- 161
          ➡ Channa_punctata                 ETC- 159
            Channa_striata                  ETC- 159
            Mus_musculus                    PEC- 167
            Homo_sapiens                    PGC- 167
Tetrapods   Anas_platyrhynchos              PPC- 168
            Chelonia_mydas                  QRC- 168
            Xenopus_tropicalis              TDC- 169
            Xenopus_laevis                  TDC- 169
            Oncorhynchus_mykiss             NYCY 171
            Salmo_salar                     NYCY 171
            Tachysurus_fulvidraco           RTC- 172
            Danio_rerio                     KIC- 166
            Ctenopharyngodon_idella         KSC- 173
            Hypophthalmichthys_molitrix     KSC- 172
```

S3 Fig
