# Supplementary material for: In silico analyses of leptin and leptin receptor of spotted snakehead Channa punctata
Source: PLoS One. 2022 Jul 7;17(7):e0270881. doi: 10.1371/journal.pone.0270881 (PMC9262212; doi:10.1371/journal.pone.0270881)
Supplement: S4 Fig — Twenty-five organisms, 19 fishes including C. punctata and representatives from each vertebrate class, were used. The extracellular domain (ECD), transmembrane domain (TMD) and intracellular domain (ICD) are underlined in orange, blue and red, respectively. The structural/functional domains are highlighted with different colors [N-terminal domain (NTD) in yellow; residues of immunoglobulin-like domain (IGD) in blue bold; ligand binding domain (LBD) in cyan; residues implicated in binding to the ligand, receptor activation, signal transduction and JAK2 activation are highlighted in red, yellow, green and purple, respectively. The conserved tyrosine Y-969, Y-1063, Y-1128 essential for signalling via SH2 containing tyrosine phosphatase 2, STAT5 and STAT3, respectively are also shown in purple]. Three fibronectin III domains (FNIII) are enclosed within black square brackets and two cytokine receptor homology (CRH) domains in curly brackets. The two conserved WSXWS motifs are enclosed in blue solid box while three conserved homology motifs box 1, 2 and 3 in black solid boxes. The residues involved in JAK2 binding are enclosed in green solid box. Three disulphide bonds formed between conserved cysteine residues (C-389- -—C-400; C-441- -—C-451; C-421- -—C-481) are highlighted in maroon solid boxes and joined by S-S bond. In multi-sequences alignment, asterisk ‘*’, colon ‘:’ and period ‘.’ indicate a site with perfect alignment, a site belonging to a group with strong similarity and a site belonging to a group with weak similarity, respectively (https://www.ddbj.nig.ac.jp/faq/en/explain-three-symbols-e.html). Solid triangles and squares represent predicted sites undergoing phosphorylation and glycosylation, respectively. (PDF) [file pone.0270881.s004.pdf]

```
Tetrapods
  Xenopus_laevis                 ----MFYHGILTFFLLIGYMQITAA----------YSEMYWTPPSDFFLTCILANKSVSY   46
  Xenopus_tropicalis             ----MFWHWILPFFLLLRYMQITAA----------YSEMYWTPPSDFFLTCILTNKSVNY   46
  Homo_sapiens                   ----MICQKFCVVLLHWEFIYVITA----------FNLSYPITPWRFKLSCMPPNSTYDY   46
  Mus_musculus                   ----MMCQKFYVVLLHWEFLYVIAA----------LNLAYPISPWKFKLFCGPPNTTDDS   46
  Chelonia_mydas                 ----MYSHKFLTVFLHLDFIRMAVA----------HCMVHQIPASSFMLPCPSPNETSVI   46
  Anas_platyrhynchos             ----MYWQIILTTAVLLGLIHAAVA----------HCMVHQILPRNFTLPCMLLNETVLS   46
Tachysurus_fulvidraco            ----MRLFV----IVNVFTAS-RAAVDVRPLNG-WP-GVYHDLQWRVQLCCALTSNGKA-   48
Danio_rerio                      ---MMSVFIMLALLVIFIAVS-QGLADLNPSDGVSD-GVYEDLKWKALLCCDHPV-QTL-   53
Ctenopharyngodon_idella          ---MMYLFIMLTLLVNFIAVS-QGLAALSPSDG-RD-GVYEDLKWKSLLCCELPFAQTV-   53
Hypophthalmichthys_molitrix      ---MMYLFIMLKLLVNFIAVS-QGLAALSPSDG-RD-GVYEDLKWKSLLCCELPFAQTV-   53
Oncorhynchus_mykiss              -MKTMILSAMLTFLVHILIIS-HSAVLVEPV-EVSPHGDLLDLPWQTELCCSSTPAQLH-   56
Salmo_salar                      -MKTMIMSAMLTFLVHILIVS-HGAVSVEPM-GVSPHGDLLDLPWQTELCCSSRPAQLH-   56
Oryzias_latipes                  ----MVRAAMLVVLIQILLIP-HGAQYLKPADGASNHLGPLGLPWQDELCCDSPSAYLG-   54
Takifugu_rubripes                MSSTMFGRVTLSVMVLGFLLS-RGVLSLENSDAGGRHSGVLDLPWKDELCCESRPAS-T-   57
Cynoglossus_semilaevis           ----MVSFPMLMILGHIFLAT-NGVWCLELRDGVSLQAGVVNLPWKDEVCCDSLT-----   50
Hippocampus_erectus              ----MAQSAMLSVLILVLLIL-TGTLCLKPEERAGLLSGVLDLPWQDQVCYDSTSAQE--   53
Oreochromis_mossambicus          MTATMVQSVMLAGLVYVFLVSY-GAQSLKPEDGASLRSGAVDLPWQDELCCESPSAHLG-   58
Oreochromis_niloticus            MTATMVQSVMLAGLVYVFLVSY-GAQSLKPEDGASLRSGAVELPWQDELCCESPSAHLG-   58
Epinephelus_coioides             MTTTMVRSVMLTVLMHF-FLVPHGAQCLEPENGASDHSGALDLPWQDELCCDSPSAHLT-   58
Scophthalmus_maximus             MTTTMSRSVMLTVLMHNILLVSHGVWCSEPEDGASLQAGALVLPWQDELCCDSRTAPFN-   59
Paralichthys_olivaceus           MTSTMFQSVMLTFLMHNILVSPAGVRCLKPEDGASLQA--LDLPWHDELCCDSHTTHFN-   57
➡ Channa_punctata                ---------MLTVLMQILLVTHAGAQGLESGGGAALHYGVLDLPWQDELCCDSPSGRLT-   50
Channa_striata                   MTTTMVPSTMLTVLMQIFLVSH-GARAFEPEGGASLQAGVLDLPWQDELCCDSPSGHLA-   58
Dicentrarchus_labrax             MTTTMVRSVMLTVLMHIFLLPH-GALGLEPDEGAFLHSGPLELPWQD----DSPSTHLT-   54
Scomber_japonicus                MNTTMVWSVMLSVLMHIFLVSH-GALCLEPEEVASLQAGALNLPWQDELCCDSPSAHLT-   58

Xenopus_laevis                   PLF-SGILQNKSDISGKHDISEDRSELRCTYLPDLKSQEHVLCCLWDNSNTNISSQNTVA   105
Xenopus_tropicalis               PLL-AGILQNKSDISGKYDTLEGRSDFRCTHLADLKSQGRVSCCLWDHLNTNFSSQNGVA   105
Homo_sapiens                     FLLPAGLSKNTSNSNGHYETAVEPKFNSSGTHFSNLSKTTFHCCFRSEQDRNCSLCADNI   106
Mus_musculus                     FLSPAGAPNNASALKGASEAIVEAKFNSSGIYVPELSKTVFHCCFGNEQGQNCSALTDNT   106
Chelonia_mydas                   PLT-TGVVASSPGLHGEHGTAE-------TNSLVLVAEESFLCCLWSETHANCSSYTAGM   98
Anas_platyrhynchos               PFS-AGVVESWSGLMREHGAAE-------TNPHLLMNEESFLCCLWSDNNIDCSLYRASM   98
Tachysurus_fulvidraco            ---NQSL-------------------------SHINSPHQYQCHIQNSTNT---------   71
Danio_rerio                      ---NSGL-------------------------SEH--PPEQHCQLLNATKQ---------   74
Ctenopharyngodon_idella          ---DSGL-------------------------SEH--QPVEQCQLLNATKL---------   74
Hypophthalmichthys_molitrix      ---DSGL-------------------------SEH--QPAEQCQLLNATKL---------   74
Oncorhynchus_mykiss              ---HGGEEED--------TGS---------APSDTGHPHTLQCLFRNFTST---------   87
Salmo_salar                      ---NRGKEED--------RGSVGSR----ATPSDPGHPHTLQCLFRNFTSI---------   92
Oryzias_latipes                  ---EDRGVTN------------RSK----TNGTISSLRHLPRCKYRRLTPE---------   86
Takifugu_rubripes                ---VEGASAP------------AER----PNGSNRSLPHDSQCSFKNLTSK---------   89
Cynoglossus_semilaevis           -----GGGKE------------DLK----TNSTESN---LLHCSIRSFTNT---------   77
Hippocampus_erectus              ---GEGSSTP------------TPE----RIRSGSMLSQHPYCYLRSISTG---------   85
Oreochromis_mossambicus          ---VEGGSAN------------SPE----ANLSQSNLPHSPGCTFKSSRID---------   90
Oreochromis_niloticus            ---VEGGSAN------------SPE----ANLSQSNLPHSPGCTFKSSRID---------   90
Epinephelus_coioides             ---AEGDDMH------------APE----TNHSESNLPQHPLCSFRGSTSE---------   90
Scophthalmus_maximus             ---EAGGDTD------------APE----TNRSESKLPHSPRCRFRSLTTG---------   91
Paralichthys_olivaceus           ----------------------IPE----TNRSESKLPHDPHCSFSSLTTE---------   82
➡ Channa_punctata    NTD         ---VEGGSTH------------APE----KSRFQSNLPHRPRCSFRSSKTK---------   82
                     (27-107)
Channa_striata                   ---VEGGSVH------------APE----TNGSESNLPHHLRCNFRSSTTQ---------   90
Dicentrarchus_labrax             ---VDGGDMH------------AQE----TNRPDSKLPHYSHCNFRSSTNN---------   86
Scomber_japonicus                ---VKVDGVH------------ALE----TNHSESSLPHYAHCNFRSLTAT---------   90
                                                                        *

Xenopus_laevis                   ELREMLFSSFA-PDIQLEDFTWNVQCSSGEKTNTLICDLQLLQETQHI--INDYQISLHY   162
Xenopus_tropicalis               ELKEMLFASFA-SDIQLEDFSWKVQCSFEEKANTLICDLQLLPETKHI--VTDYRISLHY   162
Homo_sapiens                     EGKTFV-STVNSLVFQQIDANWNIQCWLKGDLKLFICYVESLFKNLFR--NYNYKVHLLY   163
Mus_musculus                     EGKTLA-SVVKASVFRQLGVNWDIECWMKGDLTLFICHMEPLPKNPFK--NYDSKVHLLY   163
Chelonia_mydas                   ETKKFTFSEISSLTPQQTDLSWNILCWTKGDLELFVCILKLSNKKSYL--NGEVKINLSY   156
Anas_platyrhynchos               QPRMFIPSEISISASQERDSNWNTECWIKGKLDLLVCSLRFLK--LHL--RPDLKVNLLY   154
Tachysurus_fulvidraco            -TD---DSSVS--QVVSENIRLDIFCRLEDEQANVICLLKHQRTS--ATDASHLIVSLWR   123
Danio_rerio                      -Q-----S---------FGSCLDILCWLEGERENLICNAKTRRAA--AAST-LVSVSPHQ   116
Ctenopharyngodon_idella          -E-----S---------SGNCLDILCWLEGERTNLICNAKSHRAA--ATAS-LFTVSPQQ   116
Hypophthalmichthys_molitrix      -E-----SSSK--SLALSGNCLDILCWLEGERTNMICNAKSHRAA--ATAS-LFTVSPQQ   123
Oncorhynchus_mykiss              -LGPHPES------ELSRATCWDILCRVDETWDNVICDLKHPATSSDTSIPGSVALSLQH   140
Salmo_salar                      -LGPHPQT------EPSRATCWDILCRVDETWDNVICDLKHPATSSDTSTPGSVALSLQH   145
Oryzias_latipes                  -SLPQKPS---------EGNCLEILCQINEKWENLTCYLQ----PSRKLDTGGMTFSFQQ   132
Takifugu_rubripes                -LHPLEFS----------GTCLDILCGIDEKWENVTCHLEPHALPLSLPDAGHMAVSLQR   138
Cynoglossus_semilaevis           -SLPVKPSGHG--SSFNTDICLDILCRLDESWEKLTCELTSNGPTSTSREAGRMAISLQR   134
Hippocampus_erectus              -SSLNKAS---------GGTCLNILCRIDENWETLTCDLGVPRLPSARLNSAVVAVSLQR   135
Oreochromis_mossambicus          -SHHPRSS---------GGTCLDILCRIDGNWQNLICDLRSRGQPSD----SLMAVSLQR   136
Oreochromis_niloticus            -SHHPRSS---------GGTCLDILCRIDGNWRNLICDLRSRGQPSD----SLMAVSLRR   136
Epinephelus_coioides             -SHPHEPS---------GGTCLDILC[illegible]LTCDLQSHSQPSTTLDAGLMAVSLQR   140
Scophthalmus_maximus             -TRPRELSGHK--SSFNNGTCLDILC[illegible]LTCDLTSGGRTSTALDAGLMAVSLRR   148
Paralichthys_olivaceus           -SRPREASDLK--SSFNTGTCLDILC[illegible]LTCDLKSQ--TSTAEDAGLVGISFQR   137
                                                            CRH I (98-296)
➡ Channa_punctata                -SHPPQPS---------GGICLDIL{CRVDENWGNITCDLQSHSQPFGNLETGRIAVSLQR   132
Channa_striata                   -SHPLQPS---------GGTCVDILCRIDENWESVTCDLQSHDQPSGKVDTGLMAVSLQR   140
```

```
Dicentrarchus_labrax            -SRPHELS---------SGTCLNILCRIDENWENLTCDLQSHGPPSTTLDAGLMKVSLQR   136
Scomber_japonicus               -SHPHKPS---------GGTCLDMLCRIDEKWESLTCDLQSHDPPSDTMNTALMAISLQH   140
                                       :               .  *         . *                .

Xenopus_laevis                  ALVAK-------SELR---GTADCRCFGYEKCECVVPSV--KFNDSC-ILWIEILNITAH   209
Xenopus_tropicalis              SLVAK-------SELK---GTAECRCFGYEKCECIVPSV--KFNDTY-ILWIEILNITAL   209
Homo_sapiens                    VLPEV-LEDSPLVPQKGSFQMVHCNCSVHECCECLVPVPTAKLNDTL-LMCLKITSGGVI   221
Mus_musculus                    DLPEV-IDDSPLPPLKDSFQTVQCNCSLRG-CECHVPVPRAKLNYAL-LMYLEITSAGVS   220
Chelonia_mydas                  VLSELSLEDMSTDSLKGNFMVTPCDCNGSDKYECHISSL--KLNYTY-ITWLNIINGVTL   213
Anas_platyrhynchos              AVSDLPLGDVSTSSLKGTAVAAQCGCSEYGKCECCVPSP--RLNYTY-IMWLKIVTGVTP   211
Tachysurus_fulvidraco           VTLES---DP---LTNETNITTQVQCPGEDEITCFFVLQ---PNDVSVSLSVSGFLGGRP   174
Danio_rerio                     LVVQM---DV---HSDEA--NSTAQCVGEETAICSVSLH---GGDATVSLVIIISENGTT   165
Ctenopharyngodon_idella         IVLQM---DI---LSDK---NHTAHCAGEDTAMCSISLH---GNDATVLLTISISANGTT   164
Hypophthalmichthys_molitrix     IVLQM---DI---LSDK---NHTAQCAGEDTATCSISLH---GNDATVLLTISISANGTT   171
Oncorhynchus_mykiss             LTTLP---D------IEVNSTHGTDCVGEDSITCSVALH---VVSSI-VVVTANVSNTTA   187
Salmo_salar                     LATLP---D------SEVNTTHGTDCVGEDSITCSIALH---VVSSI-VVVTANVSNTTA   192
Oryzias_latipes                 LPDK----------DGTEVNSNPVVCEAEESFTCSLPLH---PAASFVTTVTVNLSSVV-   178
Takifugu_rubripes               RF-QK---SQSRVDSEEAASDPPVFCEAEDSFTCSVALD---AESSFHAVVTVTIADAR-   190
Cynoglossus_semilaevis          VSQNG---GS----QAESVSHNPVVCEDYDSIICSVVLD---STPTFISVEVVSISDNIS   184
Hippocampus_erectus             LMFHK---NYPSVDDTSMESYESVVCETKDSSRCSISIG----TTSFVSIVSVNISGCE-   187
Oreochromis_mossambicus         QLFQE---DG-----DYPASENPVVCEAQDSFMCSLTLD---PTTSFVAMVTVSISDAV-   184
Oreochromis_niloticus           QLFQE---DG-----DYPASENPVVCEAQDSFMCSLTLD---PTTSFVAMVTVSISDAV-   184
Epinephelus_coioides            LLSQK---D-AEVDDGNAATDNPVVCEAKDSFMCSVALD---TKTSFVTRVTV[illegible]   192
Scophthalmus_maximus            VLSQK---EDSLVNSGNTSPDKPVACEAKDSFVCSIALD---TTTSFIAVVTV[illegible]   201
Paralichthys_olivaceus          LLSQK---DDSRVNDGKTMSDNPVVCEAEESFSCSVALD---TTTSFISVVTV[illegible]   190
Channa_punctata                 LLSHE---GDLWVEDESASSDNPVICEALDSFMCSVAFD---STTSFVTMVTISISDAV-   185
Channa_striata                  LQSQK---DDSQVDVERSASDNPVICEALDSFMCSVSLD---ATTSCVTVVVTVSISDAV-   193
Dicentrarchus_labrax            LMSQK---EDTKVNDGNTASDNPVVCEVKDSFMCSVALN---ATTSFVIVVTVSISDAE-   189
Scomber_japonicus               LLSKK---KGSEVNYGNDASYNPVVCEAEDSFMCSLPLD---TTTSFVTMVTVSIAEAV-   193
                                                         *         * .

Xenopus_laevis                  LQSPLMSVVP---YSIVKPDPPDDLQAKIMKQGTLKVFWLKPIS--AAYELKYQVRYSV-   263
Xenopus_tropicalis              LHSPPMSVVP---YHIVKPDPPDDLRAEIMEQGTLKVFWLKPIS--AAYELQYQVRYTV-   263
Homo_sapiens                    FQSPLMSVQP---INMVKPDPPLGLHMEITDDGNLKISWSSPPL--VPFPLQYQVKYSE-   275
Mus_musculus                    FQSPLMSLQP---MLVVKPDPPLGLHMEVTDDGNLKISWDSQTM--APFPLQYQVKYLE-   274
Chelonia_mydas                  LQSPLMSVRP---INIVKPEPPLNLRLEMTDEGQLKICWFNPVI--TPYPLQYEVKFSG-   267
Anas_platyrhynchos              LWSPLMSVKP---IDIIKPEPPLNLHLEMTEKGEVRICWSDPVL--MPYPLQYEVNISG-   265
Tachysurus_fulvidraco           LQTPEMRIS---TDLLRKPEAPFYLRYNVTTEGEVMIAWNDSQN--NKLPLYYELRYSP-   228
Danio_rerio                     AQSQKMQVS---TYELQRPGAPVNLHYNVTTEGEVIFRWTGTQP--DNNTINYEIQYSS-   219
Ctenopharyngodon_idella         ALLPKMQIS---SYHLRRPDPPVNLHYNVTTEGEVIFRWSSTQP--DSNAINYEIRYSS-   218
Hypophthalmichthys_molitrix     TLSSKMQIS---SYHLRRPDPPVNLHYNVTTEGEVIFRWSSTQP--DSNAMNYEIRYSS-   225
Oncorhynchus_mykiss             GPLVMLSVPPRTSQRLWKPSPPLNLTHTQTTEGELILSWSDPQPHASPVQLSYEVRYNTS   247
Salmo_salar                     GPLVMLSVPP----RLWKPSPPLNLTHTQTTEGELILSWSDPQPHASPVQLSYEVRYNTS   248
Oryzias_latipes                 APPVLLIIPA----RPVKPSPPVNVTHYQTIEAELFVQWESPP-HFDAAQLRYEVRYNT-   232
Takifugu_rubripes               APSVLLRVPA----RPVKPAPPVNLSHVQTIEAELILHWGDPK-DIKTDLLQYEVRYSP-   244
Cynoglossus_semilaevis          SQPVYLRIPA----QPGKPSPPVNLSHVQTIQPELILMWNDAEYDPATDLMRYEVRYSF-   239
Hippocampus_erectus             ARPILLSVPA----RPVKPRTPLNLSHFQTIKPELILQWDEPK-DYGSGPLRYEVRYSN-   241
Oreochromis_mossambicus         APPVLLRVPA----RPEKPSPPGNLSHIQTIEAELIVLWDDPA-DFDAGPLRYEVRYSS-   238
Oreochromis_niloticus           APPVLLRVPA----RPEKPSPPGNLSHIQTIEAELIVLWDDPA-DFDASPLRYEVRYSS-   238
Epinephelus_coioides            APPVLLRIPA----RPVKPSPPVNLLHNQTIEADLILQWDDPS-DSDTGPLRYEVRYSS-   246
Scophthalmus_maximus            ALPVQLRIPA----RPVKPSPPVNLSHIQTTEAELILLWDGPT-DFDTGPLRYEVRYSF-   255
Paralichthys_olivaceus          APLVQLRIPA----RPVKPSPPVNLSHIQTTEAELILLWDDPS-DSSTGPLRYEVRYSF-   244
Channa_punctata                 APQVLLRIPA----RPEKPGPPVNLSHVQTIEADLIVMWDDPS-DFDAGTLRYQIRYSS-   239
Channa_striata                  APPVLLRIPA----RPEKPGPPLNLSHVQTIEAELILQWDDPS-GFDAGSLRYQVRYSS-   247
Dicentrarchus_labrax            APPVLLRVPA----RPVKPNPPVNLSHIQTIEAELILHWDDPS-NVDTGPLRYEVRYCS-   243
Scomber_japonicus               APPILLRVPA----RPVKPSPPVNLSHMQTIEAELILHWDDPE-DIDSGLLRYEVRYSS-   247
                                     : :         :*  *   :       .   :  .  *         :  *::.

Xenopus_laevis                  -KAPETNSQVYLLVNETSVIISDIQPCTEMSIEVRCINSH---KTGLWSNWSKTWVLNS-   318
Xenopus_tropicalis              -KAAETNSQVYLLVNETSVIISDIQPCTEMVIEVRCINLH---KSGLWSDWSKTWVLNS-   318
Homo_sapiens                    -NSTTVIREADKIVSATSLLVDSILPGSSYEVQVRGKRLD---GPGIWSDWSTPRVFTT-   330
Mus_musculus                    -NST-IVREAAEIVSATSLLVDSVLPGSSYEVQVRSKRLD---GSGVWSDWSSPQVFTT-   328
Chelonia_mydas                  -NATQNAWQVVEIVTETSLIIGNVLVGSSYLVQVRCKSLH---GPGFWSDWSTPYNLNA-   322
Anas_platyrhynchos              -NAGPNGWQVVRVALNTSLAIDNLLLDSSNFAQVRCKNHY---GPGFWSDWSTPYNLKLG   321
Tachysurus_fulvidraco           -NTSLTHWEVLNVQ-HPWVSLSELTSGVRYTVQVRCKSLHHLHNWSEWSNWSQPFFLTL-   285
Danio_rerio                     -NSLLQQWKVLKVKGRSWVALNELSSDIRYTVQVRCQN-----HLGYWSEWSQPFYFKL-   272
Ctenopharyngodon_idella         -NSSLQQWEVVKVRGRSWVPLNELSSGIRYTVQVRCQN-----NFNYWSEWSQPFYFTL-   271
Hypophthalmichthys_molitrix     -NSSLQQWEVVKVRGRSWVPLNELSSGIRNTVQVRCQN-----NFNYWSEWSQPFYFTL-   278
Oncorhynchus_mykiss             QSTSHPNWLHVNVSGAQWVSLTGLGPGLQYTLQIRSHHPA---LPHLWSDWSQQHRIRL-   303
Salmo_salar                     QSTSHLNWLHVEVSGCQWVSLTGLRPGLHYTVQIRSHHPA---RPHLWSDWSQQHRIRL-   304
Oryzias_latipes                 --KSDLAWQVVSVTGEPRLSL-DLQPEQEYTFQVRCSRLD---EPPLWSEWSAPYKFYQ-   285
Takifugu_rubripes               -DTIHPAWQVMSVSGDTKTSL-DLKACVNYTVQVRRSSRS---DPPLWSGWSESHHIFL-   298
Cynoglossus_semilaevis          -NRTHPDWQVVSVRGQRRVSL-DLQHSINYTTQVRRSRLG---ESSLWSDWSESYYIYI-   293
Hippocampus_erectus             --DTHSSWQVVSTGLERRLSL-DLEPEVNYTMQVHCSDLR---DPPLWSNWSEPYHIYL-   294
Oreochromis_mossambicus         -GTTHPAWQVVSAPGEPKVSL-DLKPELKYSVQVRCSGPE---EPPLWSEWSEPHHIRL-   292
Oreochromis_niloticus           -GTTHPAWQVVSAPGEPRVSL-DLKPELKYSVQVRCSGPE---EPPLWSEWSEPHHIRL-   292
Epinephelus_coioides            -NTTHPQWQVVSAPEEPRLPL-ELKPRLNYTIQVRCSGLE---NPPVWSDWSEPYHIYL-   300
```

FN III
(181-261)

```
Scophthalmus_maximus            -NTTRPAWQVMSAPAGPRLPL-DLKPRLNYTIQVRCSGPG---EPPIWSDWSESHHIYL-   309
Paralichthys_olivaceus          -TTTRPVWQVVSS-AEPRLSL-DLRPTLNYTIQVRCSGLD---EPPIWSDWSESHHIYL-   297
➡Channa_punctata               -NSTHPAWHMVSTPRDPRLSL-DLKPRLNYTIQVRCSGLD---DPPIWSNWSEPYHIYL-   293
Channa_striata                  -NITHPAWQMASALTDPRLSL-DLKPRLNYTIQVRGSRLD---NPPIWSDWSEPHHIYL-   301
Dicentrarchus_labrax            -NITHPAWQVVSALAEPRLSL-DLKPRLNYTIQVRCSSLD---KPPIWSDWSEPHHINL-   297
Scomber_japonicus               -NTNHPAWQVVSVPAEPRMLL-DLKPRLNYTIQVRCSGLD---EPPIWSDWSEPYHIYL-   301
                                                      :  :       :::         ** **     :

Xenopus_laevis                  QDVFYYPKKVLASSGSSMSVSCLFCDNGKKVPSGNITWWLNFGEKIPEHQYRAISDYFSE   378
Xenopus_tropicalis              QDVFYFPQKVLVSSGSSTSVSCLFCDNGKKVPSSNITWWLNFGEKIPKHQYRTTSDYFSK   378
Homo_sapiens                    QDVIYFPPKILTSVGSNVSFHCIYKKENKIVPSKEIVWWMNLAEKIPQSQYDVVSDHVSK   390
Mus_musculus                    QDVVYFPPKILTSVGSNASFHCIYKNENQIISSKQIVWWRNLAEKIPEIQYSIVSDRVSK   388
Chelonia_mydas                  EDVMYFPPKILTSVGSNVSFHCLYNDKNKMILSKKIVWWLNLAEEIPVRQYTLVNDRVSR   382
Anas_platyrhynchos              AEVLYFPSKILTSVGSNVSFHCIYKNQTKNVLSRKIVWWLNLAEEIPESQYTLVNDRVSK   381
Tachysurus_fulvidraco           D-VSYIPAEVFTRPGAEVTVYGVVHNHS--RTASKAVWMLNGL-VLPESQYQTINERVSA   341
Danio_rerio                     D-VSYIPAEVFTTQESEVTVYSVFHNRS--WSASKAVWFLNGKMKIPESQYRVINEQVST   329
Ctenopharyngodon_idella         D-VSYIPAEVFTTPGSEVTVYAVFHNRS--WSASKAVWMLNGQVKIPESQYSVINDQVSA   328
Hypophthalmichthys_molitrix     D-VSYIPAEVFTTPGSEVTVYAVFHNRS--WSASKAVWFLNGQVNIPESQYSVINDQVSA   335
Oncorhynchus_mykiss             ENVTYLPERVVASFGDSVTVYCVCNDLR--VNASTMVWILNSRDRLPKSQYAAVNDRVSK   361
Salmo_salar                     ENVTYLPERVVASFGDSVTVYCVFNDLR--VNASTTVWILNSRDRLPKSQYTAVNDRVSK   362
Oryzias_latipes                 YIVTYIPEKMVARAGESVTVYCLFNNRS--MNASEAVWKLNFHQLLHSSQ--SVSGRVSK   341
Takifugu_rubripes               DEVSYIPEKVVVKAGENVTVYCVFNDHN--FNASTALWTLNFDQELDYSLYHPINQWVSQ   356
Cynoglossus_semilaevis          KTVSYIPEIGLTRPGENITVYCVFNDPS--IDASMAVWTLNMNQQLPQSQYHAVNRWVSQ   351
Hippocampus_erectus             GRVSYIPEKVLAHPGENISLYCVFNDHS--INANSAVWMRNLEQPLSSSQYHSVNQRVSH   352
Oreochromis_mossambicus         DTVSYIPKIVVARPGENVTVYCVFNDHR--MNASMAVWKLNFKPPLQPTLYHPVNQWVSK   350
Oreochromis_niloticus           DTVSYIPKIVVAKPGENVTVYCVFNDHR--MNASMAVWKLNFKPPLQPTLYHPVNQWVSK   350
Epinephelus_coioides            DTVSYIPEKVVARPGDNVTVYCVFNDHS--INASTAMWMRNFQQRLHSS--QH[illegible]Q   356
Scophthalmus_maximus            DTVSYIPEKVVARPGENVTVYCVFNDRG--INASAAVWVLNFQQPLPRSQYH[illegible]Q   367
Paralichthys_olivaceus          HTVSYIPEKVVTHAGEEVTVYCVFNDHS--VNASAAIWVLNFQQTLHRSQYH[illegible]Q   355
➡Channa_punctata               DTVSYIPEKVIARPGEYVDVYCVFNDHS--INASSAVWMLNFQEPLHRNQYHPVNQWVSQ   351
Channa_striata                  DTVSYIPGKVVARPGENVTVYCVFNDHS--VNASTAVWMRNLQEPLHRSQYHPVNQWVSQ   359
Dicentrarchus_labrax            DMVSYIPEKVVVRPGENVTVYCVFNDHN--INASTAMWMLNFQEQLDHSQYHPVSQWVSQ   355
Scomber_japonicus               DTVSYIPEKVVARPGENVTVYCVFNDHS--INASTAMWMLNFQQPLHRSQYHPVNQWVSQ   359
                                  * * *    ..         .  :  .       :     *   *     :        .  .*

Xenopus_laevis                  VTLTDLNTTKPKGKFRYDALHCC---INHNECHHRYAEIYVLDV-NISISCET--DGNQK   432
Xenopus_tropicalis              VFLTHLNTTKPKGKFRYDALHCC---INHNECHHRYAEIYVLDV-NISISCET--DGNQK   432
Homo_sapiens                    VTFFNLNETKPRGKFTYDAVYCC---NE-HECHHRYAELYVIDV-NINISCET--DGYLT   443
Mus_musculus                    VTFSNLKATRPRGKFTYDAVYCC---NE-QACHHRYAELYVIDV-NINISCET--DGYLT   441
Chelonia_mydas                  VTLFNLNATKPRGKFFYNALYCC---NQNRECHHRYAELYVVDV-NINITCET--DGYLT   436
Anas_platyrhynchos              VTLFNLKATKPRGNFFYNALYCC---HQNRECHHRYAELYVVDV-NINIKCET--DGYLT   435
Tachysurus_fulvidraco           VTI------RSKEPG-FDTLLCCYPFEQSYKCSIAYTKVYVEGFFDANITCTTEQHSSVD   394
Danio_rerio                     VTL------KMDKAG-FDTLMCCLTLGEKSMCSIAYAKIYTEGRFNANITCESE-YSYVD   381
Ctenopharyngodon_idella         VTM------KVDEPG-FDTLMCCHQWGERFKCIIAYAKIYTEGMFNADITCQSK-NSEVD   380
Hypophthalmichthys_molitrix     VTV------KLDEPG-FDTLMCCLG-GEKFKCNIAYTKIYTEGMFNADITCQSK-NSEVD   386
Oncorhynchus_mykiss             ITV------RPSEQRLSGTLHCCQPFGEMYSCNYHYSTIYIKDP-VIDISCVT--SGDLD   412
Salmo_salar                     ITV------RPSEQRLSDTLHCCQPLGETYSCNYRYSTIYIKDP-VIDISCVT--NGDLD   413
Oryzias_latipes                 ITM------RASESRMYDLLTCT--Q----KAALPYSQISIEGA-SLDIRCET--NGNMD   386
Takifugu_rubripes               VTM------RPSETGMYDLLQCT--K----KRMIAYSQVYVEGA-SISISCET--NGEID   401
Cynoglossus_semilaevis          ITL------QPSQSRMYDLLQCT--Q----EWSLPCSKIYIQGS-SIDINCVT--SGGID   396
Hippocampus_erectus             ITI------QPSDSGMYELLQCRATK----EWPIPYSIIYVEGA-SIDIKCET--SGDID   399
Oreochromis_mossambicus         ITV------RPSENQMYDLLSCT--E----GWSIPYSQIYVEGA-DIDIKCVT--NGDID   395
Oreochromis_niloticus           ITV------RPSENQMYDLLSCT--E----GWSIPYSQIYVEGA-DIDIKCVT--NGDID   395
Epinephelus_coioides            ITV------RPSESQMYDLLQCT--Q----EWTIPYSQIYVQGA-SIDITCET--NGDID   401
Scophthalmus_maximus            ITV------RPSETRMYDLLQCI--Q----EWTLPYSQIYVEGA-SIDINCEA--NGDID   412
Paralichthys_olivaceus          ITV------RPSETRMYDLLQCT--K----EWTNPYSQIYVEGA-SIDINCVT--NGDID   400
➡Channa_punctata               VTV------RPSESRMYDLLQCT--Q----EWTIPYSQIYVEGA-SIDIKCET--NGDID   396
Channa_striata                  ITV------RPSETRMYDILQCT--Q----QWTIPYSQIYVEGA-SIEIKCET--NGDID   404
Dicentrarchus_labrax            ITV------RPSETRLYDLLQCT-------KWTISYSQIYVEGA-SININCET--NGDID   399
Scomber_japonicus               ITV------RPSETRMYDLLQCT--Q----EWTIPYSQIYVEGA-SIDIKCET--NGDID   404
                                :  .          :          :  *              :  :    .      .*  *  :    .
                                ----S                                                       S---------

Xenopus_laevis                  MMTCRWSSENITLPEGSSLQFKYYRNKLYCSDKDLKGN---------VPISKDCE--LQM   481
Xenopus_tropicalis              MMTCRWSSQNMTLPEGSVLQFKYYRNKLYCLDKDLKGN---------VPISKDCQ--LQM   481
Homo_sapiens                    KMTCRWSTSTIQSLAESTLQLRYHRSSLYCSDIPSIHP---------ISEPKDCY--LQS   492
Mus_musculus                    KMTCRWSPSTIQSLVGSTVQLRYHRRSLYCPDSPSIHP---------TSEPKNCV--LQR   490
Chelonia_mydas                  KMTCRWSANTNTLLVGSSLQLKYYRSSIYCSDFPSIPP---------KSEAKECH--LQR   485
Anas_platyrhynchos              KMTCRWSTNPNTLLVGSSLQLRYYRSKIYCPNFPSIPP---------ESEVKECH--LQR   484
Tachysurus_fulvidraco           TMTCKWNKSAWAV-----IRFLYRRYRRTCDEIQYEEGTLTQAQGDPMVDVEECT--AGA   447
Danio_rerio                     TMICKWNQSNWAQ-----ARLLYRQYRSKCT--------EEA--EEDTSLVKECP--SKA   424
Ctenopharyngodon_idella         TMSCEWNKSAWAQ-----VRLLYRQYTSMCETISEMEGTEEA--EENMSLVKECP--SGA   431
Hypophthalmichthys_molitrix     TMNCVWNKSAWAQ-----VRLLYRQYTSMCETISKMEGTEEA--EENMSLVKECP--SGA   437
Oncorhynchus_mykiss             SMTCRWNNLPIGG-----INFMSRVADLSCDVMEEAERVGVPV----GVVRQAKCESSGY   463
Salmo_salar                     SMTCRWNNLPIGG-----INFMSRVADLSCDVMEEAERVGVPV----GVVRQAKCESSGY   464
Oryzias_latipes                 TMECSWNSTQWLS-----FNLQHKWTHMSCERMKEKEEAGDNV----GKIVDACY-SI-K   435
Takifugu_rubripes               AMDCRWNSTQWLN-----PNFRTRWADLSCDVMEERERAGDNV----GHEGPSCL-QV-D   450
Cynoglossus_semilaevis          AMECRWNCKQWRK-----TEFQYRWAYLSCKEMEEMDRAGENV----GETGPECL-QV-T   445
```

```
Hippocampus_erectus            VMDCSWNNTWWIK-----PKFQYAWTDLPCDVMEEREKKGEEV----GELGPDCL-PV-R   448
Oreochromis_mossambicus        AMDCSWTHKQLTK-----LRFRSKWADLSCDVMEESERAGENL----GEMGPACM-EG-G   444
Oreochromis_niloticus          AMDCSWTHKQLTK-----LRFRSKWADLSCDVMEERERAGENL----GEMGPACM-EG-G   444
Epinephelus_coioides           AMTCSWKSTQWTR-----LKFRSRWADLQCDVMEERERAGEKV----GEMGPSCL-QV-R   450
Scophthalmus_maximus           AMDCSWKNTQWTK-----PTFRSRWADLPCDVMEERDRAGESV----GQMGPVCL-QV-R   461
Paralichthys_olivaceus         SMDCSWKNTQWTE-----PELRSRWADLPCDVMEERDRAGEEV----GEMGPACL-QV-R   449
Channa_punctata                AMDCSWKNTQWTK-----PTFQSRWADLPCDVMEERERAGETV----GEMAPACL-PV-G   445
Channa_striata                 AMQCSWETKQWTK-----LEFQSRWADLPCDVMEERERAGEEV----GEMGPACL-AI-G   453
Dicentrarchus_labrax           AMDCRWKNSVWTQ-----LKLKSWWANLPCDVMEERERAGENV----GEMGPDCL-QV-R   448
Scomber_japonicus              AMDCSWKNTQWTK-----PKFLSRWADLPCDVMEERERAGESV----GEMGPVCL-S---   451
                                * * *                :         *
                                                             S----S                 S-------

Xenopus_laevis                 DGFYECTFEPVHLVSGYIMWIEIHHQLGALHSPPVCVLPINTVKPLAPSRVRAEMTKGSG   541
Xenopus_tropicalis             DGFYECTFEPVHLVSGYIMWIEIQHHLGALNSPPVCILPINTVKPLAPSRVRAEMTKGSG   541
Homo_sapiens                   DGFYECIFQPIFLLSGYTMWIRINHSLGSLDSPPTCVLPDSVVKPLPPSSVKAEITINIG   552
Mus_musculus                   DGFYECVFQPIFLLSGYTMWIRINHSLGSLDSPPTCVLPDSVVKPLPPSNVKAEITVNTG   550
Chelonia_mydas                 NHYYECTFQPIFLLSGYTMWIEIKHQLGTLESPPTCVIPADVVKPFPPSNVKAEITKNVG   545
Anas_platyrhynchos             NHSYECIFQPISLLSGYTLWIEFKHSLGTLESSPTCVVPADVVKPLPPSDIKAEITRNDG   544
Tachysurus_fulvidraco          GDYYQCTLQDLSLISCYKLWLVVEDGYNKVRSLPVFVSPIDCVKPSPPSELKA-VTLPNK   506
Danio_rerio                    GDHRQCTLSQISMIFCYKFWLEVEGGRGQ--SFPVYVTPIDYVKPSPPFDLEA-ITLPSK   481
Ctenopharyngodon_idella        GDHRECTLSNLSLFSCYKIWLEVEGGRGKVRSFPVYVAPIDYVKPSPPSVLEA-ITLPNK   490
Hypophthalmichthys_molitrix    GDHRECTLSNLSLFSCYKIWLEVEGGHGKVRSFPVYVAPIDYVKPSPPSVLEA-ITLPNK   496
Oncorhynchus_mykiss            RGVKSCNLQPIRVTSCYKLWMEAKTD-NSTRSHPVYITPMDHVKPHPPSGLEA-VSMPSG   521
Salmo_salar                    RGVKSCNLQPIRVTSCYKLWMEAKTD-NSMRSHPVYITPMDHVKPHPPSGLEA-VSMPSG   522
Oryzias_latipes                --PRTCTFKPLRF-GCYKLWLELRTDSGSVRSKPIYLSSKGQVKPYTPTNVKA-VTLRSG   491
Takifugu_rubripes              SRKRLCTIQPLRT-NCYKLWLEVSSHLGLIRSKPVYLTPNDHVKPHTPTDVKA-VSRSGG   508
Cynoglossus_semilaevis         SQENMCTIQTLRM-SCYKMWLEMPSESGPISSKPIYLSPLDHVKPQPPTNVKA-VSLARG   503
Hippocampus_erectus            SKQQACTIQDIRR-NCYKLWLEMLSHQGPFRSKHIYISPLDHVKPHKPSNVKA-VNLQSG   506
Oreochromis_mossambicus        --QETCTIHPLRM-NCYKLWLELPSQLGPIRSKPVYLSPVDHVKPHAPANVKA-VSHSSG   500
Oreochromis_niloticus          --QETCTIHPLRM-NCYKLWLELPSQLGPIRSKPVYLSPVDHVKPHAPANVKA-VSHSSG   500
Epinephelus_coioides           SKQKTCTIQPLRM-NCYKLWLEVPS[illegible]KPIYLSPIDHVKP[illegible]A-VSRSSG   508
Scophthalmus_maximus           SRQKSCTIQPLRM-NCYKLWLEVPS[illegible]KPIYLSPIDHVKP[illegible]A-VSRSSG   519
Paralichthys_olivaceus         SKQKSCIIQPLRM-NCYKLWLEVAS[illegible]KPVYLSPIDHVKP[illegible]A-VSRSSG   507
Channa_punctata                SRQKSCTIQPLRM-NCYKLWLEVPSRLGPIRSRPIYLSPIDY[illegible]KPHTPTNVKA-ESLSSG   503
Channa_striata                 SRHESCIIHPLRM-NCYKLWLELPSRLGPVRSRPIYLSPIDNVKPHMPTDLKA-ESLSTG   511
Dicentrarchus_labrax           SQQKTCHIQPLRM-NCYKLWLEMPSRLGPIRSKPIYLSPIDHVKPHTPTNVKA-GSQSSG   506
Scomber_japonicus              --QKSCTIQPLRM-NCYKLWLEVPSRLGPIWSKPVYLSPIDHVKPHTPTNVRA-VSRSSG   507
                                     * :  :      * :*:       .    *     :    . ***   *   :.*   .
                               -----S

Xenopus_laevis                 YLYVSWKRPALPSTDLQFQVCYRLQ---NKGVIWKVLDIFKEE--FASVQVPDICASYTV   596
Xenopus_tropicalis             HLYVSWKRPALPSTDLQFQVRYCLQ---GQGIIWKVLDIFEEE--FVSIQVPDVCASYTV   596
Homo_sapiens                   LLKISWEKPVFPENNLQFQIRYGLS---GKEVQWKMYEVYDAKSKSVSLPVPDLCAVYAV   609
Mus_musculus                   LLKVSWEKPVFPENNLQFQIRYGLS---GKEIQWKTHEVFDAKSKSASLLVSDLCAVYVV   607
Chelonia_mydas                 LLNVSWTNPAFSNSDLKFQIRYSVN---REEILWEIFEVSNAPTRSAMIKVLDLCVVYIV   602
Anas_platyrhynchos             LLNVSWTNPVFTNDDLKFQIQYAEK---REELTWELYEVSNLPTRSAMIKVQKLCVEYVV   601
Tachysurus_fulvidraco          TLSATWKRPYLPAYDLQYELRYVSMH-GMVDLKWKVFGSLLES--RATFTVLDPCIQYQV   563
Danio_rerio                    TLSVRWKRPSLPVYGMQYELQFKALA-GMANTQWKVIGPLLEP--QAEIQLEESCVHFKV   538
Ctenopharyngodon_idella        TLSVKWGRPHLPVYDMQYELRFVTLR-GMANTQWKVIGSLLEP--QAEVPLEDSCVQFKV   547
Hypophthalmichthys_molitrix    TLSVKWRRPHLPVYDMQYELRFVALR-GMANTQWKVIGSLLEP--QAEIPLEDSCVQFKV   553
Oncorhynchus_mykiss            VLKLAWVPPELPIYDMQYQVRYA-LSTGKAHPFWQVLALQTES--WAEVLEPDVCGVYNV   578
Salmo_salar                    VLKLAWVPPELPIYDMQYQVRYA-LSTGRAHPFWQVLALQTES--WAEVLEPDVCGVYNV   579
Oryzias_latipes                VLSVTWEPPSLPIDGLQYELQYHPLSTVK--EEWKVQRSKQPP--PMTVQVPEMCRSYVV   547
Takifugu_rubripes              VLNVTWKRPYLPVE-VQCQFRYHSPSADHPKPDWKVQAIVREP--WAEVNVSDVCRVFVV   565
Cynoglossus_semilaevis         VLSVTWECPLLPVEGLQCQFQYHSPSAVRAQPEWKVQSPVRDP--WSEVVVPHMCRVYVV   561
Hippocampus_erectus            ILRITWEPLPLHVQGLQCQFRYHSPSAIKAQPEWKLSNPVWVP--SAEIPVPDMCQVYAV   564
Oreochromis_mossambicus        VLEVTWQAPPLPADGLQCQFQYHSPSTVSPRPKWKLQDPVRVP--WAEVAVPDMCRVYVV   558
Oreochromis_niloticus          VLEVTWQAPPLPADGLQCQFQYHSPSTVSPRPRWKLQDPVRVP--WAEVAVPDMCRVYVV   558
Epinephelus_coioides           VLLISWEPPSLPVEGLQCQFRYHSPSAVRAQPEWKIQSPVRVP--WAEVLVPDMCRVYVV   566
Scophthalmus_maximus           VLAVTWEPPSLPVDGLQCQFQYHSPSMVRAQPEWKVQSPVRVP--RAEVVVPDMCRVYVV   577
Paralichthys_olivaceus         ALLVTWKPPFLPVEGLQCQLRYHSLSMVRAQPQWKVQSPVRVA--LSEVAVPDMCQVYVV   565
Channa_punctata                VLVVSWEPPPLPVEGLQCQFRYHSPSTARAQQEWKVQSPVRAP--QAEISVPDMCRVYVV   561
Channa_striata                 VLLVSWEPPSLPVEGVQCQFQYHSPSTARAQPEWKVQSPVRVS--WAEVVVPDMCRVYVV   569
Dicentrarchus_labrax           VLTVTWEPPSLPVDGLQCQFRYHSPSAVRAQPEWKVQSPVRVP--WAEVAVADMCREYAV   564
Scomber_japonicus              VLTITWEPPSLPVEGLQCQFRLHSLSTVRAQPEWKVHNPVRVQ--WAEVAVPDMCRVYVV   565
                                *   *    :    :: :.              *:              .   . *  : *

Xenopus_laevis                 QISSKRIDGAGYWSDWSQPVHTVVRDIRVPLQGPAFWRTTPMNNPMKKGENITLFWQPLP   656
Xenopus_tropicalis             QVRSRRTDGVGYWSDWSQPVHTVVRDIRVPLQGPTFWRTT-HNNPMQKGDNISIIWQPLP   655
Homo_sapiens                   QVRCKRLDGLGYWSNWSNPAYTVVMDIKVPMRGPEFWRII-NGDTMKKEKNVTLLWKPLM   668
Mus_musculus                   QVRCRRLDGLGYWSNWSSPAYTLVMDVKVPMRGPEFWRKM-DGDVTKKERNVTLLWKPLT   666
Chelonia_mydas                 QVRCSGLDGLGYWSDWSKPAYTIVQDIQAPLRGPEFWRVI-NEDPIRKQKNVTLVWKPLM   661
Anas_platyrhynchos             QVRCRALDGLGYWSNWSKSAYAVVRDIKAPLQGPEFWRVI-VEDPARRQKNVTLLWKPLM   660
Tachysurus_fulvidraco          QVRCRRLNGPGYWSDWSYTHASSVYNVKAPEMGPDFWRII-QETPEP-YTNVTLLFK-PL   620
Danio_rerio                    EVRCKDVNDTGYWSDWSNSHISTVFNLKAPEMGPDFWRIL-QEDPTRNVTNVTLIFKQPI   597
Ctenopharyngodon_idella        EVRCRRLNGSGYWSDWSRSHTSIVYNRKAPEMGPDFWRII-QEDPVRSVTNVTLIFKQPV   606
Hypophthalmichthys_molitrix    EVCCRRLNGPGYWSDWSRSHTSIVYNRKAPEMGPDFWRII-QEDPVRSVTNVTLIFKQPV   612
Oncorhynchus_mykiss            QVRCMHINGSGTWSDWSHLLYTTTHNSRAPDQGPDFWRVF-QEDPASMQTNVTLLFEHSP   637
```

FN III (470-553)

CRH II (488-686)

```
Salmo_salar                  QVRCRHINGSGTWSDWSHLLYTTTHNSRAPERGPDFWRVF-QEDPASTQTNVTLLFEHSP  638
Oryzias_latipes              QVRCMHIAGKGYWSEWSDLIYSTPNNSKAPERGPDFWRIR-QDNQHINKSNITLLFEHFP  606
Takifugu_rubripes            QVRCMHISGAGYWSEWSPSVYSSPQNSRAPERGPNFWRFL-QDDPHRKQTNVTLLFKDLQ  624
Cynoglossus_semilaevis       QVRCMPANGSGYWSEWSETVYSTPQNSRAPERGPDFWRVL-QDDPKTNQTNVTLLFEDLP  620
Hippocampus_erectus          QVSCKPANRTGYWSDWSDSVYSVPQNSQAPEHGPDFWRLL-ENDQLTNSTNVTLLITPLP  623
Oreochromis_mossambicus      QVRCKHTNGTGYWSDWSESVYSTPQNSRAPERGPDFWRIR-QDDPHGNQSNITLLFENFP  617
Oreochromis_niloticus        QVRCKHTNGTGYWSDWSESVYSTPQNSRAPERGPDFWRIR-QDDPHGNQSNITLLFENFP  617
Epinephelus_coioides         QVRCMHTSGTGHWSEWSDSVYSTPQNSRAPERGPDFWRVL-QDDPYRNQTNVTLLFEQHH  625
Scophthalmus_maximus         RVRCMHTNGTGYWSEWSDSVYSAPQNSRAPERGPDFWRVL-QGDPDRNQTNVTLLFEDLP  636
Paralichthys_olivaceus       QVRCMHTNGTGYWSEWSDSVYSAPQNSRAPERGPNFWRIL-QDDPYRNQTNVTLLFKDLP  624
➡Channa_punctata             QVRCMHVNGTGYWSDWTDSVYSTPQNSRAPERGPDFWRIL-QDEPYRNQTNVTLLFEQMP  620
Channa_striata               QARCMHINGTGYWSDWSKSVYATPQNSRAPERGPDFWRIL-QDDPYRNQSNVTLLFEQIP  628
Dicentrarchus_labrax         QVRCMPTNGTGYWSDWSNSVYSTPQNSRAPERGPDFWRIL-QDDPYRSQTNVTLLFKNLQ  623
Scomber_japonicus            QVRCMHTNGTGYWSDWSDSIYSTPQNSRAPDRGPDFWRVL-QDDPYRNQTNVTLLFQPLL  624
                             .  .      *  **:*:    :   : :.*   ** ***           *:::.

Xenopus_laevis               TK--QSLCSIQGYEVVLQNSKNFTWSKYVG-NATKHTFTLSDNAVTVTLLAVNSLGYSLT  713
Xenopus_tropicalis           SE--HSLCSIQGYEVIHLNSKNVTWSKYVG-NTTKHTFTLSDNAVTVTLLAVNSLGYSLT  712
Homo_sapiens                 KN--DSLCSVQRYVINHHTSCNGTWSEDVG-NHTKFTFLWTEQAHTVTVLAINSIGASVA  725
Mus_musculus                 KN--DSLCSVRRYVVKHRTAHNGTWSEDVG-NRTNLTFLWTEPAHTVTVLAVNSLGASLV  723
Chelonia_mydas               KN--YSLCSVCGYIVKHHTSENITWTEYVT-SGTPCTFPWTEDAHTITVLAVNSIGASSM  718
Anas_platyrhynchos           KN--HSLCSVSRYIIKHQTSENTTWSEYVD-KGTTWLFPWTERTHTITVLAMNSIGVSSR  717
Tachysurus_fulvidraco        -PEVEAAICVQGLVVVHQTSGGNVWSDDIIAPSSFYTFQWREEVHSITVMSRNSLGSSAE  679
Danio_rerio                  -LAGDPNSCVEGLVIKHQASGGVMWSNETT-LARFHSFQWRKEAHTVTVMSRNALGISTW  655
Ctenopharyngodon_idella      -LAGDPYSCVEGLVIKHQASGGAVWSNETT-LTQFHSFQWRKEAHTVTVMSRNTLGISTR  664
Hypophthalmichthys_molitrix  -LAGDPYSCVEGLVIKHQASGGAVWSHETT-LAQFHSFQWRKEAHTITVMSRNTLGISTR  670
Oncorhynchus_mykiss          IV--EPTYCVEELVVQHQDSGGTVTEERIG-LVSSYSFEWRGEVHSVTVKAQNSQGSSTR  694
Salmo_salar                  IV--EPTYCVEELVVQHQDSGGTVTEERIG-LVSSYSFEWRKEVHSVTVKAQNSQGSSTR  695
Oryzias_latipes              GT--WNSYCVDGFIVQHEASNRSVVRKQIN-LGSSYSFEWNQEPQTVTVEAYNSLGNSTN  663
Takifugu_rubripes            TS--GQPYCVEGFLVKRLGSTGPVQ-EPIL-MQSSYSFEWNQMPQTVTVEAFNSLGSSSD  680
Cynoglossus_semilaevis       TS--RHSYCVDGFKIHHQTSTGPVISRSVE-LVSSYSFEWNQEVQTVTVEAYNSLGSSTN  677
Hippocampus_erectus          KM--SRSYCIDGYIVQQQTSNGIVKREKIE-LLSSYSFEWKQDLQSVTVEAYNSLGSSRK  680
Oreochromis_mossambicus      PS--GNSYCVDGFVVQRRSSSGSVLRETIE-LMSSYSFEWNQELQTVTVEAYNSLGNSRD  674
Oreochromis_niloticus        PS--GNSYCVDGFVVQRRSSSGSVLRETIE-LMSSYSFEWNQELQTVTVEAYNSLGNSRD  674
Epinephelus_coioides         LQLSARSYCIDGFIVQYQALSGSVMREQIE-LASSFSFEWNQVPQTVTVEAY[illegible]  684
Scophthalmus_maximus         VS-GHSSHCVDGFIVQHQTSGGAVTREWIE-PASSYSFEWNQELQTVTVEAY[illegible]  694
Paralichthys_olivaceus       VS-GR-SYCVDGFIVQHQTSSGIVTRNSIE-PASSYSFEWNQEPQTVSVKAYNSRGSSAN  681
➡Channa_punctata             IS--GPSYCVDGFIIQLQASSGTVMREQIE-VVSSYSFEWNQDLHTVTVEAYNNIGSSTN  677
Channa_striata               IS--GHSYCVDGFIIQHQTSSGAVMREQVE-VVSSYSFEWNQDLHTVTVEAYNSLGSSTN  685
Dicentrarchus_labrax         MS--GQSYCVDGFIVQRQTSSGSVIREKIQ-LVSSYSFEWNQVLQTVTVEAYNSLGSSAN  680
Scomber_japonicus            TS--GHSYCVDGFIVQHQSSSGTVIRERIE-LVSSYSFEWNQEVQTVTVEAYNSLGSSAN  681
                                     .:    :                     *       :::: : *  * *

Xenopus_laevis               NSKLTFSCEMSTVTSVASLRVYLMNNTCAVAVWTMLPKRDMPLEFIVEWKN---------  764
Xenopus_tropicalis           NSKLTFSCEMSTVTSVESFRVYHMNNTCAVAVWTMLPKSDMPLEFIVEWKN---------  763
Homo_sapiens                 NFNLTFSWPMSKVNIVQSLSAYPLNSSCVIVSWILSPSDYKLMYFIIEWKN---------  776
Mus_musculus                 NFNLTFSWPMSKVSAVESLSAYPLSSSCVILSWTLSPDDYSLLYLVIEWKI---------  774
Chelonia_mydas               NFNLTLSQQMSTVNIVQSLSAYPVNSSCVILTWTLSPQMYVITSFFLEWKN---------  769
Anas_platyrhynchos           NFNLTLSQQLSTVNAVQSLIAYPVNSTCVILTWTLLPHTYVITSFVIEWKS---------  768
Tachysurus_fulvidraco        NSNMTLVRQ-PKRQCVRWFHV-TANASCVFLSWSLLSEQPSLLSFVLEWQE-QSGVSSQG  736
Danio_rerio                  NRNITLLRQ-AKRRCVRSFSA-VANVSCVHLSWSLLSDQPVPQSFVIEWLD-LNKDPEKD  712
Ctenopharyngodon_idella      NSNMTLLHQ-PKRRCVRLFSG-VANASCVHLSWSLLSDQPVPQSFVIEWID-LNKDPEQD  721
Hypophthalmichthys_molitrix  NSNMTLLHQ-PKRRCVRSFSG-VANASCVHLSWSLLSDQPVPQSFVIEWLD-LNKDPEQD  727
Oncorhynchus_mykiss          NTHMTLDRH-PKRQCVRSFSASRVNSSCVVLLWSLQPNSSVPWSLVVEWSG-QNHQDRPD  752
Salmo_salar                  NTHMTLDRH-PKRQCVRLFSASRVNSSCVVLLWSLQPNSSVPWSLVVEWSG-QNHQDRPD  753
Oryzias_latipes              NKNMTLGKT-SRRKAVHSVHALVLNSTHVSLSWSLLNDGIVPLFMVVQWSE---------  713
Takifugu_rubripes            NINMTLEKS-PKRRCVHHFSVTVINSTCVSLSWTLIDKSSPPIFMVVQWSL-LWKQDSGR  738
Cynoglossus_semilaevis       NINMTLEKP-PKRHCVQNFHVLVVNSTCVSLSWSLLENSSVPLFMVVQWFP-HWQ---HH  732
Hippocampus_erectus          NMIMTLDRR-PKRRSVRSFRVLLINGTCVSLHWSLVDNSSTPLFMVVQWAP-QRQDGFDL  738
Oreochromis_mossambicus      NINMTLERQ-PKGHCVRSFHVLLINGTCVSLSWSLLENSSVPLFMVVEWLP-HKQQDS--  730
Oreochromis_niloticus        NINMTLERQ-PKGHCVRSFHVLLINGTCVSLSWSLLENSSVPLFMVVEWLP-HKQQDS--  730
Epinephelus_coioides         NFNMTLERQ-PKRRSVRSFSVLVINSTCVSLSWTLLDNNSVPLFMVVQWSP-HKQQDSDH  742
Scophthalmus_maximus         NINMTLERQ-PKRHCVRSFRVLLTNSTCVSLSWSLLDNSSAPLSMVVQWSP-HRQPESGR  752
Paralichthys_olivaceus       NINMTLEQK-PKRRCVSSFSVSVINSTSVSLSWILLDNRSVPVSMVVQWTVPQRQQGSDH  740
➡Channa_punctata             NINMTLENQ-PKRRCVQSFRVLVINSTCVSLSWSLLDNSSAPLFMVVQWLP-QTQQDSDL  735
Channa_striata               NINMTLESQ-PKRRSVQSFRVLVINSTCVSLSWSLVENTSAPLFMVVQWLP-QRQQ--DL  741
Dicentrarchus_labrax         NINMTLERQ-PKRHCVRSFSVRVINSTCVSLSWSLLDNSSVPMFLVVQWSP-QRQPDSGY  738
Scomber_japonicus            NINMTLGRQ-PKRRSVRAFRALVINSTCVSLSWSLLDNSSVPLFMVVQWLP-QRQQDPHH  739
                             *  :*:         *  .    . : .   * :        :.::*

Xenopus_laevis               LGNEKKVRWMNIPGNASRCYIEDNFFAIEKHQFSLYPIFPEGVGGSKVIKGFSTV-EITE  823
Xenopus_tropicalis           LGNEEKVQWMNIPRNMSRCYIEDNFFAIEKYVFSLYPVFPEGVGRSKVVNGFSTV-ELTE  822
Homo_sapiens                 LNEDGEIKWLRISSSVKKYYIHDHFIPIEKYQFSLYPIFMEGVGKPKIINSFTQD-DIEK  835
Mus_musculus                 LNEDDGMKWLRIPSNVKKFYIHDNFIPIEKYQFSLYPVFMEGVGKPKIINGFTKD-AIDK  833
Chelonia_mydas               LNEEEQMKWIRVAPNISKYYIYDHFILIEKYQFSLYPIFPEGVGNPKTTDGFIK--DRSE  827
Anas_platyrhynchos           LNKEEEMKWVRVPPNTSKYYIYDHFILIEKYHFSLYPVFAGGVGKSRATDRFTKVPDGYE  828
Tachysurus_fulvidraco        WASDGRVEWLRVASTARDLQLCRPFYGTEE--FKLYPVFVDGEGEAVRCTA---------  785
```

FN III (670-760)

```
Danio_rerio                       VSLTERIQWVRVESRSRDLSLCRRFYGSEE--FTLYPVFADGEGEPARYTA---------   761
Ctenopharyngodon_idella           VSLTERLQWVRVHSASRDLSLCRRFYGSEE--FTLYPVFADGEGEPVRYTA---------   770
Hypophthalmichthys_molitrix       VSLTERLQWVRVQSASRDLSLCRRFYGSEE--FTLYPVFADGEGEPVRYTA---------   776
Oncorhynchus_mykiss               QTPESRERWTRFPPTDKLLYLYGHFYDT-EYEFILYPVFADGEGEPVYTKVFRG------   805
Salmo_salar                       QTSESRERWTRFPPTDKLLYLYGHFYDTDEYEFILYPVFADGEGEPVYTKVFRG------   807
Oryzias_latipes                   SSGLSGLKWARLPYSNHVVYIKGSFSRSEDYSFHLYPVFADMEGEPMYIIA---------   764
Takifugu_rubripes                 PRGQSTDTWVRLPYTDGPTYLGGHFFGSEDYGFYLYPVFAHGEGEPAFATA---------   789
Cynoglossus_semilaevis            HKVQSRKTWARLPYTAGPVYLTGEFFGSEEYGFYLYPVFAHGEGEPVYTLAA--------   784
Hippocampus_erectus               SKAHSGRTWARLPSTDCSVHLTGDFYASEEYGFYLYPVFSEGEGEPAFTIA---------   789
Oreochromis_mossambicus           --GPRAETWTRLRYTDHPVYLRGDFFASEEYGFFLYPVFAEGEGEPIYTLA---------   779
Oreochromis_niloticus             --GPRAETWTRLRYTDHPVYLRGDFFASEEYGFFLYPVFAEGEGEPIYTLA---------   779
Epinephelus_coioides              HKGRIGETWARLPYTDHPIYLKGDFFGSEECGFYLYPVFADGEGEPVYAIA---------   793
Scophthalmus_maximus              PRAQGGETWARLPYVDHPVYLRGDFFSSEDYGFYLHPVFAEGEGEPVFTIA---------   803
Paralichthys_olivaceus            QKAYSGETWVRLPYTDCPVHLRGDFFGSEEYGFHLYPVFADGEGEPMYTIA---------   791
Channa_punctata                   HKRHNGGTWARLSYTDRPVYLRGDFFASEEYVFHLYPVFADGEGEPMYTTA---------   786
Channa_striata                    HRSPNGESWARLPYADHPFYLRGDFFASEGYVFYLYPVFADGEGEPVYTTA---------   792
Dicentrarchus_labrax              HKGQSGETWARLPYTDRPTYLRGDFFGSEECGFSLYPVFADGEGEPIYTTA---------   789
Scomber_japonicus                 HKGLSGEPWVRVPYTDRPVYLRGDFFGSEEYGFYLYPVFVEGEGEPAYTTA---------   790
                                          * ..        :   *       * *:*:*    *
                                                              Box 1 (819-830)
Xenopus_laevis                    APKDTGLYVILPVISFFALLLMGTILISHQRMKQIFWKDVPNPKHCSWAQGVNFEKPDTL   883
Xenopus_tropicalis                APKDAGLYVILPVISFSVFLLMGTILISHQRMKKLFWKDVPNPKHCSWAQGVNFEKPDTL   882
Homo_sapiens                      HQSDAGLYVIVPVIISSSILLLGTLLISHQRMKKLFWEDVPNPKNCSWAQGLNFQKPETF   895
Mus_musculus                      QQNDAGLYVIVPIIISSCVLLLGTLLISHQRMKKLFWDDVPNPKNCSWAQGLNFQKPETF   893
Chelonia_mydas                    KRNDAGIYVILPIVISSFVLLLGTLLISQQRMKKLFWEDVPNPKNCSWAQGVNFQKPETF   887
Anas_platyrhynchos                NQNNASLYMVLPIVVSTLVLLLGALLISHQRMKKLFWEDVPNPKNCSWAQGVNFQKPETF   888
Tachysurus_fulvidraco             VRSDPAAYMLLMIIAFLFVVLFVTLIISQNQLKKLMWRDVPNPNNCSWAKGIDFKKLD--   843
Danio_rerio                       TRGDPAAYILLLIIAFLSVVLFVTLMMSQNQMKKLMWKDVPNPNKCSWAKGMDFRQIDTM   821
Ctenopharyngodon_idella           TRSDPAAYILLLIIAFLSVVLFVTLVMSQNQMRKLVWKDVPNPNKCSWAKGMDFRQIDTM   830
Hypophthalmichthys_molitrix       TRSDPAAYILLLIIAFLSVVLFVTLMMSQNQMRKLMWKDVPNPNKCSWAKGMDFRQIDTM   836
Oncorhynchus_mykiss               SDAGPAAYMLLMIIAFLSIVLFVTLVISQNHMKKFMWKDVPNPNNCSWAQGIDFGKADTM   865
Salmo_salar                       GDAGPAAYMLLMIIAFLSIVLFVTLVISQNHMKKFMWKDVPNPNNCSWAQGIDFGKADTM   867
Oryzias_latipes                   AKRNPAAYMIIMSISFLCIL-LLTLVLTQNQIKRN---LVPNPKKCSWAKGIDFQKVDTF   820
Takifugu_rubripes                 TRRDPAIYMMLMMISFLSIVLLISLILSQNQMKKLMWKDVPNPNQCSWARGIDLN---AF   846
Cynoglossus_semilaevis            PRGDPAAFMMVLLVSFLSVILVVILILSKNQMKRFVWKDVPDPNKSSWAKGKDFKQA-GF   843
Hippocampus_erectus               TRGDPSAYMTLLIIALLSIVLFITLVLSQNQMKRFVWKDVPNPKKCSWAKGLNFKKADTF   849
Oreochromis_mossambicus           TRGDPKAYMMLMIISFLFIILFVTLVLSQNQMKKFVSKDVPNPRKCSWAKGIDFKKVDTF   839
Oreochromis_niloticus             TRGDPEAYMTLMIISFLFIILFVTLVLSQNQMKKFVSKDVPNPRKCSWAKGIDFKKVDTF   839
Epinephelus_coioides              SRGDPAAYMMLMIISFLSIVLFVTLILTQNQMKRFVWKDVPNPNKCSWAKGLDLKKADNF   853
Scophthalmus_maximus              TRADPAAYMMLMIISFLAIVLFVTLLLSQNQMKRFMWKDVPNPNKCSWAKGLDFKKADTF   863
Paralichthys_olivaceus            TRGDPAAYMMLMIISFLSIVLFVTLIISQNQMKKFVWKDVPNPYKCSWAKGLDFKKNDTL   851
Channa_punctata                   ARRDPAVYMMLMIISFLSVVLFVTLVISQNQMRSLVWKDVPNPNKCSWAKGLDFKKADNF   846
Channa_striata                    TRRDQALYMMLMIISFLSVVVFVTLVLSQNQMRRFVWKDVPNPNKCSWAKGLDFKKAETF   852
Dicentrarchus_labrax              TRGDPAAYMMLMIISFLSIVLFVTLVLSQNQMKKFMWKDVPNPNKCSWAKGLDFKKADTF   849
Scomber_japonicus                 TRGDPAAYMMLMIISFLSIVLFVTLVLSQNQMKKFMWKDVPNPNKCSWAKGLDFKKADTF   850
                                     .   :: :  :    .: .  :::::::::      **:* :.***:* ::
                                                 Box 2 (860-875)
Xenopus_laevis                    ENLFRKHYRHPANGFRFLYEPEAVIKDLKIDKQVPNEITDNISKAISLFTATEEPGHD-S   942
Xenopus_tropicalis                ENLFMKHHKHPANGSPFLFEPEAVFEDLSIDKQVPHEIIDNIPAVTSLFTVSEEPDHD-S   941
Homo_sapiens                      EHLFIKHTASVT-CGPLLLEPETISEDISVDTSWKNKDEMMPTTVVSLL-STTDLEKG-S   952
Mus_musculus                      EHLFTKHAESVI-FGPLLLEPEPISEEISVDTAWKNKDEMVPAAMVSLLLTTPDPESS-S   951
Chelonia_mydas                    EHLFIKHPEAIS-FGPLLLEPEIVLEDINVAKALKSEDKQDLLAVDSMFTKIQDSEHD-S   945
Anas_platyrhynchos                EHLFIKHPEAMS-SEPLLLEPEIVLEDISVTKVLKQEDTQDFLVINSMFTKIQDSEHD-S   946
Tachysurus_fulvidraco             GNLFSHHEGLT--ACPLLPTSENVCEVEIVEKLFVLEDDQEE--K-ALLHRSVDTEAK--   896
Danio_rerio                       ESLFPHSEGLT--ACPLLLVSESICEVEIIEKPHPLTIENVKDNE-EL--PSGD--KT--   872
Ctenopharyngodon_idella           ENLFPHSEGLT--ACPLLLVSESICEVEIIEKCHPLMLEHEKDNE-VLIYNSGN--KA--   883
Hypophthalmichthys_molitrix       ENLFPHSEGLT--ACPLLLVSESICEVEIIEKCHPLMHEHEKDNE---VYNSGN--KA--   887
Oncorhynchus_mykiss               EQLFRHPEGLP--AWPLLLVSETISQATIMEKTGPPTSGSD---K---------------   905
Salmo_salar                       EQLFLHPEGLP--AWPLLLVSETISQATIMEKTGPPTSVPD---K---------------   907
Oryzias_latipes                   D-LFQPAEGLQ--ICPLLPSDNIISKVIIMEKVEKR----AFMET-QLMSLNDDSVTSSS   872
Takifugu_rubripes                 DHMFHPPEGFP--AWPLLLPPEKISNLVIVDKADLSALSTP-----------PDPSVASS   893
Cynoglossus_semilaevis            QHLFQTPEGLP--AWSLLLPSENISHVVIVDKPDLCTLTRAISLP---------------   886
Hippocampus_erectus               HELFQSSDILP--AWPLLLPSENISKVVIMEKANMSVLSGALVRS-PLITPTLDAVSCPF   906
Oreochromis_mossambicus           DYLFRPREGLP--VWPLLMPSENISQVIIVDKV----LTTALIQN-PL----PDHADALA   888
Oreochromis_niloticus             DYLFRPREGLP--VWPLLMPSENISQVIIVDKV----LTTALIQN-PL----PDHADALA   888
Epinephelus_coioides              DHLFQPAESLS--AWPLLLPAENISKVVIVDEVDLSALTTALIQV-PLVSLTPDPDTALS   910
Scophthalmus_maximus              DHLFGPPEGLP--AWALLLPSEDLSKVVIVDKADLCAATTALVQT-PLVPPATDPAAASA   920
Paralichthys_olivaceus            DHLFGPSEGLP--SWSLLMPSENISKVVIMDKADLSAPTTALVQT-PLVPLTTDPATTLS   908
Channa_punctata                   DHLFQPVEGLQ--AWPLLLPSENISKVIVVEKADLSDLTTALIQT-PLVSLSSDPAVP--   901
Channa_striata                    DNLFQPVEGLQ--AWPLLLSPENISKVVLVEKADLSGLTTALIQT-PLVSLSPDPAVS--   907
Dicentrarchus_labrax              DQLFRPPEGLP--PWPMLLPSENISKVVIVDKVDLSALTTVLVQT-PLVSLTPDPATTL-   905
Scomber_japonicus                 DHLFQPPEGLP--ARPLLLPSENISKVVIMEKTDLSALT-------PLVSLNPDTATALA   901
                                    :*            :*   : : .   :

Xenopus_laevis                    AS--NHFNIDCAL-----------------------------------------END   956
Xenopus_tropicalis                ACESSNFSSGCAF-----------------------------------------ETD   957
Homo_sapiens                      VCISDQFNSVNFS-----------------------------------------EAE   968
```

```
Mus_musculus                  ICISDQCNSANFS-------------------------------------------GSQ  967
Chelonia_mydas                ACSSSHFSN-CLS-------------------------------------------ESS  960
Anas_platyrhynchos            ACSSSHFTSSSFS-------------------------------------------ESF  962
Tachysurus_fulvidraco         ----------------SN--NLSSIEGSLDPLSLD------------------------  913
Danio_rerio                   ----------------TT--DSGLQGDSSEA--LE------------------------  887
Ctenopharyngodon_idella       ----------------NT--DSACLGDSSEPLSLQ------------------------  900
Hypophthalmichthys_molitrix   ----------------NT--DSACLGDSSEPLSLQ------------------------  904
Oncorhynchus_mykiss           --------------------DLIPASSPALCVDSE--VPGLPEEEESLQ----------  932
Salmo_salar                   --------------------DLIPASSPALCVDSE--VPGLPEEEETLQ----------  934
Oryzias_latipes               ACLAPPFERSCLD-----------ASAP-----------SSQSLDEANQA----------  900
Takifugu_rubripes             VRLHGEPDSPVGQ-AWPEESHLLPGGDRSSPPNLDYPTGSAPDD----------------  936
Cynoglossus_semilaevis        ----PESDTELDHSGEFTELSGLMMGEAPPLDHLNTSTMSLPRIDDLLPDDSR-------  935
Hippocampus_erectus           I-------------------EVDQNVIPSSILDLDILTNPRPQLDELQLIDPP-------  940
Oreochromis_mossambicus       GSHSPGFDLNVD---QFMENETLPVGGPSSAVDLDTLTSSSSRADLQP-ADPS-------  937
Oreochromis_niloticus         GSPSPGFDLNVD---QFMENETLPVGGPSSAVDLDTLTTSSSRADLQP-ADPS-------  937
Epinephelus_coioides          ISLPPGYDSEADQ-AQATESEVLLSGAPSLAHDTDALTSSSPTTDQLQLIHPLAEQPGST  969
Scophthalmus_maximus          ISLPPGFDSEVDH-SELTESELLLGGAPPLAVNADALTGSYPRIDEFQPVVSQAAQP---  976
Paralichthys_olivaceus        ISLHPGIDSEVDQ-GQFRESEVLLGGAPSLDLNLDALTSSYPRIEELLPVVS---QP---  961
Channa_punctata               --LTSVFDSKDEQ-TQFMNRELLLEGAASLAVNLDALNNFHPEIDELQSDPSP-------  951
Channa_striata                --LPPGL--EDQQ-TQFMGREMLPDGAPSLALNLDALTSSHPKIEALQPDDSP-------  955
Dicentrarchus_labrax          --PPPGFDSEVDQ-TPSLESEELQGGALSFALNLDDLTSSSPEIDQLELDNLP-------  955
Scomber_japonicus             ISIPSGFNSEVDQ-TRSMDSEALLGGAPSLTLDMDALTASSLRTDELQLVDLS------R  954

Xenopus_laevis                HQEMVYSS--ICQSSIKYATILGND------QQCRKYSSERKTSLSSFDGCLLGNSSMVI  1008
Xenopus_tropicalis            HQEMVYSS--ICQSSIEYATIMNNT------QQCRKYSSERKTSLSSFDGCLLGNSSMVI  1009
Homo_sapiens                  GTEVTYEAESQRQPFVKYATLISNS------KPSETG-EEQGLINSSVTKCFSSKNSPLK  1021
Mus_musculus                  STQVTCEDECQRQPSVKYATLVSND------KLVETD-EEQGFIHSPVSNCISSNHSPLR  1020
Chelonia_mydas                HDDK-VSEGITRQSNIKYATIISNC------KSSGLC-EQQKNLSGSFNGCFLGEDSLVT  1012
Anas_platyrhynchos            HNDQ-IAGGIMRQSDIRYATIISNS------RSGGLY-EEKKNLRSCFERCFLAEDSSVA  1014
Tachysurus_fulvidraco         TSTVSASPDTSGQSSVRYSTILVFD------QPVLQRKQQESLSSSSDEGNFSANNSDIS  967
Danio_rerio                   ASTAAPTPETSGQSSVTYSTILLSD------QPSQLQKQQESLSSSSDEGNFSANNSDIS  941
Ctenopharyngodon_idella       ASTAAATPETSGQSSVTYSTVLLSD------QPTLLRKQQESLSSSSDEGNFSANNSDIS  954
Hypophthalmichthys_molitrix   ASTAAATPETSGQSSVTYSTILLSD------QPTLLRKQQESLSSSSDEGNFSANNSDIF  958
Oncorhynchus_mykiss           LPDLPRSLESSAQPSVTYATVLLSD------NPHHLYKQEGSLSSSSDEGNFSGNNSDIS  986
Salmo_salar                   LPDLPRSLESSAQPSVTYATVLLSD------DPHHLYKQEGSLSSSSDEGNFSGNNSDIS  988
Oryzias_latipes               --DPIVPVDSSTSSSVRYAKLLLPCLKQEKQP--GNPDKDGSGSNSSDEGNFSANNSEIS  956
Takifugu_rubripes             GSCPAGVTDSSAQSSVIYTAVLLCGPKQQQ-QHHHLHDKDCSCSSSSDEGNFSANNSDIS  995
Cynoglossus_semilaevis        TDQHPDSAENSAQSSVTYSTVMPPDMKEEQ----HCHYREGSGSSSSDEGNFSANNSDIS  991
Hippocampus_erectus           ----PSVLENSAQSSVAYAAVLLTNAKQEL-QSIHLSDRDGSGSSSSDEGNFSADNSDIS  995
Oreochromis_mossambicus       VNQHPGSTENSGQSSVTYTAVLVSNPSQDQQPPIHRPYKDGSGNSSSDEGNFSANNSDIS  997
Oreochromis_niloticus         VDPHPGSTENSGQSSVTYTAVLVSNPSQDQQPPIHLPYKDGSGNSSSDEGNFSANNSDIS  997
Epinephelus_coioides          DSSAQSSAQNSAQSSVTYATVLLPDMKQEQ-PPIHLHYKDGSGSSSSDEGNFSANNSDIS  1028
Scophthalmus_maximus          ----PGSADNSAQSSVTYATVLLSDPKQEQ-PPVHLHYKGSSGSSSSDEGNFSANNSDIS  1031
Paralichthys_olivaceus        ----PGSTDNSAQSSVTYTTVLLSDPKQEQ-PPIHLHYKGGSGSSSSDEGNFSANNSDIS  1016
Channa_punctata               EVQPPGSTVSSAQSSVTYATVLISHPKQDQ-QHICLRYKDGSGSSSSDEGNFSANNSDIS  1010
Channa_striata                EDQPPGSTVSSAQSSVTYATVLLSQPKQEQ-QHICLHYKDGSGSSSSDEGNFSANNSDIS  1014
Dicentrarchus_labrax          ADQSRGSTDSSAQSSVTYATVLLSDPKQEQ-QPNHLHYKDGSGSSSSDEGNFSANNSDIS  1014
Scomber_japonicus             TDQLPGTNNNSAQSSVTYATVLLINPKQEQ-QPIHLHYKDGSGSSSSDEGNFSANNSDIS  1013
                                          .  : *: ::                       .     : .. * :

Xenopus_laevis                GN-----HDVDKHTLVFLAGLHTKQPNKICCNSTVSSEGSCESLDPEESFL-------DA  1056
Xenopus_tropicalis            GN-----HDVDKQTLVFLAGLHTKQPDKMSCNSTVSSEGFSEPLDHEDSFL-------EA  1057
Homo_sapiens                  DSFSNSSWEIEAQAFFILSDQHPNIISPHL----TFSEGLDELLKLEGNFP-------EE  1070
Mus_musculus                  QSFSSSSWETEAQTFFLLSDQQPTMISPQL----SFS-GLDELLELEGSFP-------EE  1068
Chelonia_mydas                DPFS-RSWEVGNQAFLILPDQHPSQASKTISLSVVSSEGFSEPSDHDDIFS-------DG  1064
Anas_platyrhynchos            GAFSSSSWEVGNQAFLLLPDQPGSRPSKTLSLSLISSEGFSEPSDQDDTFP-------DG  1067
Tachysurus_fulvidraco         GSFPGGLCELERQSS----SD---AINPRNSCSYNSVEEFSETSEQEDEAS-------ES  1013
Danio_rerio                   GSFPGGLWDLEN-------------SNPRHSSSYNSVEEFSETSEPDYEAS-------EN  981
Ctenopharyngodon_idella       GSFPGGLWDLDNHVC----SD---STNPRHSSSYNSVEEFSETSEQDYEAS-------ES  1000
Hypophthalmichthys_molitrix   GSFPGGLWDLENHVC----SD---STNPRHSSSYNSVEEFSETSEQDYEAS-------ES  1004
Oncorhynchus_mykiss           GSFPGGLWELEISHS----GAGESDLDPRRSCSYNSIEEFSETSEQEDEALGGERDGGIE  1042
Salmo_salar                   GSFPGGLWELEISHS----GTGESDLDPRHSCSYNSVEEFSETSEQEDEALGGERDGGIE  1044
Oryzias_latipes               ESSPTGLWELDSCHS----AE---MDDQRRFCSYASEGELSEISEHEAV-M--------E  1000
Takifugu_rubripes             ASFNGGLWEL---------------DVPRRSCCYNSTEELSEKPEQGDR----------D  1030
Cynoglossus_semilaevis        ESIPCGLQELGSCQG----GE---VDDPRRSCSYNSVEELSETSEQEDE-G--------E  1035
Hippocampus_erectus           GSFPGGLWELESCHD----RE---VDDPRRSCSYNSVEELSEPSDQEDA-----------  1037
Oreochromis_mossambicus       GSFPGGLWELESCRG----LE---MDDPRRSCSYNSVEELSQNSDQEEE-H--------E  1041
Oreochromis_niloticus         GSFPGGLWELESCRG----LE---MDDPRRSCSYNSVEELSQNSDQEEE-H--------E  1041
Epinephelus_coioides          GSFPGGLWELDSCRG----GE---IDDPRRSCSYNSVEELSETSEQEDEEE--------E  1073
Scophthalmus_maximus          GSFPGGLWELDSCRG----GE---TDDPRRSCSYNSVEELYETSEQEDD-G--------E  1075
Paralichthys_olivaceus        GSFPGGLWELESCRG----GE---MDDPRRSCSYNSVEELSETSEQEDE-G--------E  1060
Channa_punctata               GSFPGGLWELDSCRG----GE---SEDQRRSCSYNSVEELSETSEQEDE-G--------G  1054
Channa_striata                GSFPGGLWELDSCRG----GE---MEDPRRSCSYNSVEELSETSEQEDE-G--------D  1058
Dicentrarchus_labrax          GSFPGGLWEL---------------DDPRRSRSYNSVEELSETSEQGDE-E--------A  1050
Scomber_japonicus             GSFPGGLWELESCRG----GE---MDDPRRSCSYNSVEELSETSEQEDE----------E  1056
                                      :                                :  .
```

```
Xenopus_laevis                   DGLERNLYYLEFGSVQQCGQQEYYSEKPLGT---------FHYK-------ENISYKEID  1100
Xenopus_tropicalis               DGLERNLYYLEFGSIQQCGQQDCYSEKPLGT---------FPFQ-------ENISYKEID  1101
Homo_sapiens                     NNDKKSIYYLGVTSIKKRESGVLLTDKSRVS---------CPFP-------APCLFTDIR  1114
Mus_musculus                     NHREKSVCYLGVTSVNRRESGVLLTGEAGIL---------CTFP-------AQCLFSDIR  1112
Chelonia_mydas                   GSPERSLYYLGLTSIKKNENEIFLTENSRVM---------CHLH-------TNGLFKDMG  1108
Anas_platyrhynchos               GSPERSLYYLGIASLEKRESDIFLTESSGVM---------CQFH-------TADLLRDVG  1111
Tachysurus_fulvidraco            IQVSKELYYIGMNEEEEEDELEEKFKEDDATKE---------------------------  1046
Danio_rerio                      TGLAKDLYYLEMTGEEKEEEEEEEEEEEEEDEPEEGQSK----------NKRVMGVNPRP  1031
Ctenopharyngodon_idella          TGVAKDLYYLEMNEEEKQEEDE------DIKEAQDGQDK----------NEIVMRVDARP  1044
Hypophthalmichthys_molitrix      PGVAKDLYYLEMNEEEKQEEDE------DIEEA---QDK----------NEIVMRVGARP  1045
Oncorhynchus_mykiss              IIEEKDLYYLGMDCQEESE--------E------EEDTRAMLLKEVMVLGRGGSSVESIP  1088
Salmo_salar                      VIEEKDLYYLGMGYQEESEGEEEEEEKE------EEDTGAMLLKEVMVLGREGSSVESIP  1098
Oryzias_latipes                  QRREQTLCYLQIGYPDEDEESAEEVQREEEEKRKE------QPAKDASLNG---------  1045
Takifugu_rubripes                VGEEKDLYYIGADYGDEDEESEEELN--------------AKLIQTVPLNSEGCSAESRR  1076
Cynoglossus_semilaevis           AGREKDLYYLKVEYPEEEEEEDEEGEREDNEWHREGAAK-LKVHKNILLNRSDCCVESQP  1094
Hippocampus_erectus              -TEEKDLYYLSMDYQAENEEGEGEVKQK------------EELLKSVTLSRDGCSVESHL  1084
Oreochromis_mossambicus          VRREKDLYYLGMDYPAEDEESEKEDGQS-----EDEEAK-VELLKSTPLNRGHCSLELHP  1095
Oreochromis_niloticus            VRREKDLYYLGMDYPAEDEESEKEDGQS-----EDEEAK-VELLKSTPLNRGHCSLELHP  1095
Epinephelus_coioides             AREEKDLYYLGMDYPAEDEESEEDKEQ---------IED-IELLKNVVLSREDCSVELHP  1123
Scophthalmus_maximus             ARDEKDLYYLGMDYPAEDEASEDEDERR------EEAAK-IDLRKNVVLTRDDCSVESRP  1128
Paralichthys_olivaceus           GRGEKDLYYLGTDYPAEDEESEDEEEQR------E------ELRKNVVLSREDCCVESRP  1108
Channa_punctata                  TKEGKNLYYLGMDYPAEDEDSEEEEEQR------EEETK-IELLKIVVPD-QSFSGESRP  1106
Channa_striata                   AKEGKDLYYLGMDYPAEDEESEEEEEQR------EEETK-IELLKNVVLDMESCSVELHP  1111
Dicentrarchus_labrax             VREEKDLYYLGMDYRAEDEESEEDEEQR------EEETK-IELLKNVVLNREDCSVESHP  1103
Scomber_japonicus                VREGKHLYYLGMDYPAEDEESDEEEEQR------EEDTK-TELLTSVVLNRDACSVESHP  1109
                                      : : *:        .
                                                               Box 3
                                                            (1128-1131)
Xenopus_laevis                   FKKDKDP---EFI-DNYDIKSSFQKTFLQYMPQFQKHSITLPGEMEAE---TLNLCT      1150
Xenopus_tropicalis               FKKDKAS---EFI-DNYDIKNSFKKAFLQYMPQFQTHSIKLPGEMESE---TLN---      1148
Homo_sapiens                     VLQDSCS---HFVENNINLGTSSKKTFASYMPQFQTCSTQTHKIMENK---MCDLTV      1165
Mus_musculus                     ILQERCS---HFVENNLSLGTSG-ENFVPYMPQFQTCSTHSHKIMENK---MCDLTV      1162
Chelonia_mydas                   FLQGTTSDLNPFIKNSLKYEN-SVKTFVPYMPQFQATTVKLQETTETK---T-----      1156
Anas_platyrhynchos               FLQNTPPSLNTFIQSSFNYK-----AIVPYMPQFQMTAAKVQDATENS---S-----      1155
Tachysurus_fulvidraco            -----------------QISDSGDSNVPLYLPQFQTAAIKLLREPAGNS--TIQL--      1082
Danio_rerio                      LLESQNS---T---A--SNSNNMSHSIPLYLPQFRSECINPT---------------      1065
Ctenopharyngodon_idella          LLEGKNS---T---V--VNSNNVSRSIPLYLPQFQTECINPP---------------      1078
Hypophthalmichthys_molitrix      LLEGKNS---T---I--VDSNNVSPSIPLYLPQFQTECINPP---------------      1079
Oncorhynchus_mykiss              LLGSQDS---MFSEYSDEGSVVGMRSVPLYLPQFRTAPSSPLKAQDS----------      1132
Salmo_salar                      LLGSQDS---MFSEYSDEGLVVGMRSVPLYLPQFRTVPSSPLKAQDSAHQL------      1146
Oryzias_latipes                  -------------KDFFV--PLICDLSSQYMPQYRTAA-YRSQLV------------      1074
Takifugu_rubripes                LLE-------------LT--ESKCDFSPLYLPQFRTAPSCTRQLSAKPQEGRCHP--      1116
Cynoglossus_semilaevis           LLRAEDP---GTPVETL--------ASLIYLPQFRTAP-CVRQLSESQTQL------      1133
Hippocampus_erectus              T-----------PTELVS--TSTCDLAPLYMPQFRTAP-YSRQLPD-----------      1116
Oreochromis_mossambicus          LLGQDNP---SEPGILPS--PSTCGFAMPYLPQFRTAT-CTAQHTQREPQL------      1140
Oreochromis_niloticus            LLGQDNP---SEPGILPS--PSTCGFAMPYLPQFRTAT-CTAQHTQREPQL------      1140
Epinephelus_coioides             LLSPEDS---S---ELLL--ASTRGFSSLYLPQFRTAP-YTRQLTAQPHDGKPQQ--      1169
Scophthalmus_maximus             LLCPGDS---SSRTGPLS--ASTRGFTPLYLPQFRTAP-CTRQLQDSEPEL------      1173
Paralichthys_olivaceus           LLDPEDS------SGPMS--DSTGGLSPLYLPQFRTAA-CTRQLQDSRTQL------      1150
Channa_punctata                  LLGSGEP------TEPLS--ASTCGFTPLYLPQFRTVP-CTNQLQDS----KAQI--      1148
Channa_striata                   LLGPGGP------TEPLS--ASTRGFSPLYLPQFRTVP-CANQPQDS----EAQL--      1153
Dicentrarchus_labrax             LLTPEDS------SELLS--ASTRGFCPLYLPQFRTAP-NTRQLTAQPQDSKPQV--      1149
Scomber_japonicus                LLGPVDS---SDNSEMDS--ASTRGFSLIYMPQFRTAP-CTRQLTD-----------      1149
                                                             *:**::
```

S4 Fig.
