# Supplementary material for: In silico analyses of leptin and leptin receptor of spotted snakehead Channa punctata
Source: PLoS One. 2022 Jul 7;17(7):e0270881. doi: 10.1371/journal.pone.0270881 (PMC9262212; doi:10.1371/journal.pone.0270881)
Supplement: S7 Fig — Multiple sequence alignment of leptin (lepa) and leptin receptor of C. punctata with respective homologs in other teleosts highlighting the sites on which selection pressure acted. Color key: yellow box indicates positively selected sites by FUBAR; blue box shows negatively selected sites by FUBAR and green box shows evidence of episodic diversifying selection detected by MEME. (PDF) [file pone.0270881.s007.pdf]

A

```
TACHYSURUS_FULVIDRACO            XXXXXXXXXXXXMAVXYPALFCSCVXVTVLTL---TNGXXXXXXRALPTDSLXXKNSVKL     57
DANIO_RERIO                      XXXXXXXXXXXXXMRXFPALRSTCIXLSMLSL---IHXXXXXXXXCIPVHQHDXRKNVKL     57
CTENOPHARYNGODON_IDELLA          XXXXXXXXXXXXXMYXSPVLLYTCFXLSILGM---IDGXXXXXXRSIPIHQDNLKNLVKL     57
HYPOPHTHALMICHTHYS_MOLITRIX      XXXXXXXXXXXXXMYXFPVLLYTCFXLSILGL---IDGXXXXXXRSIPFHPESLKSXLKQ     57
ONCORHYNCHUS_MYKISS              XXXXXXXXXXXXMDCXSMALLLSSLXLALFSVGAGXXX---XXXASLSLHVVXXRTKVKD     57
SALMO_SALAR                      XXXXXXXXXXXXMDCXSMALLLSSLXLALFSVGAGXXX---XXXASLSLHVVXXRTKVKD     57
ORYZIAS_LATIPES                  XXXXXXXXXXXXMDSXALVLFAFLXXFHCLNVATAXXX---XXXAPVNPELQEMKSNVID     57
TAKIFUGU_RUBRIPES                XXXXXXXXXXXXMDHXILALVLALL---PLSLCVALPGXXXXXXALDAMDVEKMKSKVTW     57
HIPPOCAMPUS_ERECTUS              XXXXXXXXXXXXMDCITLAILVSVXXSQVWGAVTAXXX---XXXAPMSVEVIRMKATVEG     57
SPARUS_AURATUS                   MAKGLYTIPTYSKLYXTIPTYSKRSHLDAQSAGTAAPA---PATAPVPAEVVKMKSKVKW     57
CYNOGLOSSUS_SEMILAEVIS           XXXXXXXXXXXXMYCXSLAFLLPLXXLHLLSICTAXXX---XXXASLPMEVVKVKSNVKQ     57
SCOPHTHALMUS_MAXIMUS             XXXXXXXXXXXXMDYXTLVLLFSLXXLQVLSVCTAXXX---XXXAPLPVEVVKMKSKVKW     57
PARALICHTHYS_OLIVACEUS           XXXXXXXXXXXXMDYXTLALLFSLXXLQVLSVCTAXXX---XXXAPLPAEVVMMKSKVKW     57
SCOMBER_JAPONICUS                XXXXXXXXXXXXMDYSTLTLLISLXXSQLLSVGTAXXX---XXXAPLSVEVGKMKIKVKW     57
DICENTRARCHUS_LABRAX             XXXXXXXXXXXXMDSXTLAILFSMXXLQLLIVSTAXXX---XXXAPLPVEVVKMKSKVKW     57
OREOCHROMIS_NILOTICUS            XXXXXXXXXXXXMDYXGLVLLFSLXXFQALSMGTAXXX---XXXAPLPVEVVTMKSKVKW     57
OREOCHROMIS_MOSSAMBICUS          XXXXXXXXXXXXMDYXGLVLLFSLXXFQALSMGTAXXX---XXXAPLPVEVVTMKSKVKW     57
CHANNA_ARGUS                     XXXXXXXXXXXXMDYXTLALVFSLXXLQVLSAGTAXXX---XXXAXXPVEVVKMKSKVKG     57
EPINEPHELUS_COIOIDES             XXXXXXXXXXXXMDYXTLALLFSLXXLHVFSVGTAXXX---XXXAPLPVEVVKMKSKVKW     57
CHANNA_PUNCTATA                  XXXXXXXXXXXXMDYXTLALLFSLXXLHLLTAGSAXXX---XXXAXXPVDVVKMKSKVKW     57
CHANNA_STRIATA                   XXXXXXXXXXXXMDYXTLALLFSLXXLQLLSVGTAXXX---XXXAXXPVEVVKMKSKVKW     57
                                                                                  .    :  :

TACHYSURUS_FULVIDRACO            QVENIISRIQKHKDEFPILHKMILDSPELLPELQSDKPIEGLSSMVEMLNNFQRVLHSLP     117
DANIO_RERIO                      QAKTIIVRIREHIDGQNLLPTLIIGDPGHYPEIPADKPIQGLGSIMETINTFHKVLQKLP     117
CTENOPHARYNGODON_IDELLA          QADTIIHRIKEHNEKLKLSPKILIGDSELYPEVPADKPIQGLGSIVDTLTTFQKILQTLP     117
HYPOPHTHALMICHTHYS_MOLITRIX      QADTIIHRIKEHNEKLKLSPKILIGDSELYPEVPADKPIQGLGSIIDTLTTFQKVLQTLP     117
ONCORHYNCHUS_MYKISS              LAQTMVIRIKKXXXXLDISPNLIEGMDPFLPAAAVDQHIESLPSIVETMGFYQDLLLVLD     117
SALMO_SALAR                      LAQTMVIRINKXXXXLDISPNLIEGMDPFLPAAAVDQHIESLPSIMETMGFYQDLMLFLD     117
ORYZIAS_LATIPES                  IAKELSLRLESIXXXIQTSIGPKFSXXXXXXXXPPSDELNGLSSIMAVLDECTNQISDXN     117
TAKIFUGU_RUBRIPES                KAQGLVARIDKHXXXFPXDRGLRFXXXXXXXXXXDTDKVEGSTSVVASLESYNNLISDXR     117
HIPPOCAMPUS_ERECTUS              KSKQLVARLNKXXXXIQVPPGMTLTXXXXXXXXPPADRLDGLSSVVTLLDGYDKLISDXS     117
SPARUS_AURATUS                   IAEQLLVRLNRDXXXFQAPLGFQVSXXXXXXXXPRADDLDGTSSIVMVLEGYNSLISDXD     117
CYNOGLOSSUS_SEMILAEVIS           LSEQLLVRLKNXXXXFQFPAGRTLSXXXXXXXXPAAGDLDRLSATVMILDGYNSLISDXT     117
SCOPHTHALMUS_MAXIMUS             MAEQLIIRMEDXXXXFQFPSSLTXXXXXXXXXXXPTDDLDGASSIVTVLEGYNSLISDXT     117
PARALICHTHYS_OLIVACEUS           MAEQLVVRLDKDXXXFNFSVSLTXXXXXXXXXXXPTDDLAGASSIVTVLEGYNSLISDXT     117
SCOMBER_JAPONICUS                MAEQLVAKLNNXXXXFQVPAGWTLSXXXXXXXXQPADDVDGLSSIVVILDGYNSLISDXS     117
DICENTRARCHUS_LABRAX             MAEQLVVRLNRDXXXFQVPIGLTLSXXXXXXXXPPADDLDGLSSIVTILEGYNSLISNXS     117
OREOCHROMIS_NILOTICUS            MAEQLVVRLDKDXXXVQVPVNWTLNXXXXXXXXPPADDLDGTSSIETVLNGYNSLIPDXT     117
OREOCHROMIS_MOSSAMBICUS          MAEQLVVRLDKDXXXVQVPVNWTLNXXXXXXXXPPADDLDGTSSIVTVLNGYNSLIPDXT     117
CHANNA_ARGUS                     LAEQLVVRLNKDXXXIQVAPSFTLSXXXXXXXXPPTDVLDGSSSVVRVLEGYNSLISDXG     117
EPINEPHELUS_COIOIDES             MAEQLVVRLNKDXXXFQVPPGLTLSXXXXXXXXPPADILDGPSSIVTVLDGYNSLISDXT     117
CHANNA_PUNCTATA                  MAEQLVVRLNKDXXXFQAPAGLTISXXXXXXXXPPADDLDGSSSIVMVLEGYNSLISDXT     117
CHANNA_STRIATA                   MAEQLVVRLNKDXXXFQVPAGLTLSXXXXXXXXPPADNLDGSSSIVMVLEGYNSLISDXT     117
                                   . :  ::                             :    :    :      :

TACHYSURUS_FULVIDRACO            KGHMSQLHSDVSTLQHYLEDRMSSLQCXXXTHRITGTXXEKNLEDFPKNHSMYYITVRHV     177
DANIO_RERIO                      NKHVDQIRRDLSTLLGYLEXXXXGMDCXXXTLKESTNXXGKALDAFLEDSASYPFTLEYM     177
CTENOPHARYNGODON_IDELLA          KGHVSQLHNDMSTLLEYFKDRMTFMRCTLKEPANXXXXXGKSLDTFIEKNATHHITFGYM     177
HYPOPHTHALMICHTHYS_MOLITRIX      KGHVSQLHSDVSTLLDYFKVWMTFMRCTPKEPANXXXXXGKSLDTFIQKNATHHVTFGYM     177
ONCORHYNCHUS_MYKISS              WADLKQLVEDTSTMRGLLENWMTSXRCPARQQKQTGEXXGGLSEALKDTTRKYGLSVGPV     177
SALMO_SALAR                      WADLKQLVEDTSTMRGLLENWMISXRCPGRQQKQTGEXXGRLSEALKDTTRKYGLSVGPV     177
ORYZIAS_LATIPES                  FDEAKKIKVDISSLMDSMSEWSDKXHCGEQPSTQAENXXXXXXXXXXQTSRRFSITESMQ     177
TAKIFUGU_RUBRIPES                FGGVSQIKTEISSLAGYLNHWREGXNCQEQQPKVXXXXXXXXXXXXWPRRNIFNHTVSLE     177
HIPPOCAMPUS_ERECTUS              LNXVSQVKAEISWLKSYLGQWKKGXRCGEAKANRTSATGGALQRXXLQSQRSFVLTVGIE     177
SPARUS_AURATUS                   LDNVSQIKHDISSLSGFLDQWRQEXHCTGQRPKPSVPXXGPLQEXXLQSKKEFIHTVSIE     177
CYNOGLOSSUS_SEMILAEVIS           LNGVSQIKADISCLTRHLDQWRQGXHCNKQRPKPSVPXXEPLQKXXLQSHKDLIDTVSFE     177
SCOPHTHALMUS_MAXIMUS             FDGVSQVKSEISSLTGYVDQWRRGXHCSEQRPKPSVPXXGPLQNXXLLSRKEFVHTVTIE     177
PARALICHTHYS_OLIVACEUS           FHRVSQVKSEISSLTGYVDQWRKGXHCSEQRPKPSMPXXGPLQKXXLQSLKDFIHIVSME     177
SCOMBER_JAPONICUS                LNGVSQVKSEISSLGGYLQQWKKGXHCNEQRPKPLVSXXGPLQEXXLQSRKEYIHTVGIE     177
DICENTRARCHUS_LABRAX             LDGVSQVKVDISSLTGFLSQWRQEXHCSEQRPKLSVPXXGVLQEXXLQRRKTFIHTVSIE     177
OREOCHROMIS_NILOTICUS            FKGVSQIKYDISSLTGYIHLWRQGXHCSEQRPKPEVPXXGPLQEXXLQSHKEFIQTVGIE     177
OREOCHROMIS_MOSSAMBICUS          FKGVSQIKYDISSLTGYIHLWRQGXHCSEQRPKPEVPXXGPLQEXXLQSHKEFIQTVGIE     177
CHANNA_ARGUS                     FNDVSQVKFDISSLTGYLVHWRQVXHCTEQRPKPLVSXXGRLQEXXLQSQKQFIHTVSME     177
EPINEPHELUS_COIOIDES             FNGVSQVKFDISSLTGYIGQWRQGXHCTEQRPKPSVPXXGPLQEXXLQSRKEFIHTVSIE     177
CHANNA_PUNCTATA                  FNGVSQVKVDISSLTGYLDQWRQVXHCTEQRPKPLVSXXGPLQEXXLQSRKEFIHTVSIE     177
CHANNA_STRIATA                   LNGVSQVKFDISSLTGYLNQWRQVXHCTEHRPKPLVSXXGPLQEXXLQSRKEFIHTVSIE     177
                                     .::  : * :   .        *
TACHYSURUS_FULVIDRACO            ALDRLQKYIQRLNHNLEQLRTCXXXXXXXXXXXXXXXXXXXXXXXXXXXXX   228
DANIO_RERIO                      TLNRLKQFMQKLIDNLDQLKICXXXXXXXXXXXXXXXXXXXXXXXXXXXXX   228
CTENOPHARYNGODON_IDELLA          ALDRLKQFMQKLIANLDQLKSCXXXXXXXXXXXXXXXXXXXXXXXXXXXXX   228
HYPOPHTHALMICHTHYS_MOLITRIX      ALDRLKQFMQKLIANLDQVKSCXXXXXXXXXXXXXXXXXXXXXXXXXXXXX   228
ONCORHYNCHUS_MYKISS              ALNRLKGYLGRLLLNLDQLNYCYXXXXXXXXXXXXXXXXXXXXXXXXXXXX   228
SALMO_SALAR                      ALNRLKGYLGRLLLNLDQLNYCYXXXXXXXXXXXXXXXXXXXXXXXXXXXX   228
ORYZIAS_LATIPES                  AVTRLKHFLLLLQNNSDQLEICXXXXXXXXXXXXXXXXXXXXXXXXXXXXX   228
TAKIFUGU_RUBRIPES                ALMRVREFLKLLQKNVDLLERCXXXXXXXXXXXXXXXXXXXXXXXXXXXXX   228
HIPPOCAMPUS_ERECTUS              ALVRVKDILTRMLQNMEHLDKCXXXXXXXXXXXXXXXXXXXXXXXXXXXXX   228
SPARUS_AURATUS                   ALMRVKEFLNLLLKNLDNLKTCXXXXXXXXXXXXXXXXXXXXXXXXXXXXX   228
CYNOGLOSSUS_SEMILAEVIS           ALLRVKEILVVLLKNLDNLEICXXXXXXXXXXXXXXXXXXXXXXXXXXXXX   228
SCOPHTHALMUS_MAXIMUS             ALMRVKELLNLLLKNLDHLESCXXXXXXXXXXXXXXXXXXXXXXXXXXXXX   228
PARALICHTHYS_OLIVACEUS           ALMRVKEFLNLLLKNLDHLETCXXXXXXXXXXXXXXXXXXXXXXXXXXXXX   228
SCOMBER_JAPONICUS                AVMRVKEFLTLLLKNLDQLKTCXXXXXXXXXXXXXXXXXXXXXXXXXXXXX   228
DICENTRARCHUS_LABRAX             ALMRVKEFLNLLLKNLNHLKTCXXXXXXXXXXXXXXXXXXXXXXXXXXXXX   228
OREOCHROMIS_NILOTICUS            ALMRVKEFLNLLLKNLDQLETCXXXXXXXXXXXXXXXXXXXXXXXXXXXXX   228
OREOCHROMIS_MOSSAMBICUS          ALMRVKEFLNLLLKNLDQLETCXXXXXXXXXXXXXXXXXXXXXXXXXXXXX   228
CHANNA_ARGUS                     ALMRVKLFLNLLLKNLDHLEICXXXXXXXXXXXXXXXXXXXXXXXXXXXXX   228
EPINEPHELUS_COIOIDES             ALMRVKEFLNLLLKNLDHLETCXXXXXXXXXXXXXXXXXXXXXXXXXXXXX   228
CHANNA_PUNCTATA                  ALMRVKEFLHLLLKNLDNLETCXXXXXXXXXXXXXXXXXXXXXXXXXXXXX   228
CHANNA_STRIATA                   ALMRVKEFLYLLLKNLDHLETCXXXXXXXXXXXXXXXXXXXXXXXXXXXXX   228
                                 :: *::  :  :  * : :  * ****************************
```

S7A Fig

B

```
PELTEOBAGRUS_FULVIDRACO          XXXXXXXXXXXXMRLF----VIVNXVFTASRXAAVDVRPLNGXXWPGVYHDLQWRVQLCC     56
DANIO_RERIO                      ----XXXXXXXMMSVFIMLALLVIXFIAVSQXGLADLNPSDGVSXDGVYEDLKWKALLCC     56
CTENOPHARYNGODON_IDELLA          ----XXXXXXXMMYLFIMLTLLVNXFIAVSQXGLAALSPSDGXXRDGVYEDLKWKSLLCC     56
HYPOPHTHALMICHTHYS_MOLITRIX      ----XXXXXXXMMYLFIMLKLLVNXFIAVSQXGLAALSPSDGXXRDGVYEDLKWKSLLCC     56
ONCORHYNCHUS_MYKISS              ----XXXXXMKTMILSAMLTFLVHXILIISHXSAVLVEPVEXVSPHGDLLDLPWQTELCC     56
SALMO_SALAR                      ----XXXXXMKTMIMSAMLTFLVHXILIVSHXGAVSVEPMXGVSPHGDLLDLPWQTELCC     56
ORYZIAS_LATIPES                  ----XXXXXXXXMVRAAMLVVLIQXILLIPHXGAQYLKPADGASNHLGPLGLPWQDELCC     56
HIPPOCAMPUS_ERECTUS              ----XXXXXXXXMAQSAMLSVLILXVLLILTXGTLCLKPEERAGLLSGVLDLPWQDQVCY     56
CYNOGLOSSUS_SEMILAEVIS           ----XXXXXXXXMVSFPMLMILGHXIFLATNXGVWCLELRDGVSLQAGVVNLPWKDEVCC     56
TAKIFUGU_RUBRIPES                ----XXXXMSSTMFGRVTLSVMVLXGFLLSRXGVLSLENSDAGGRHSGVLDLPWKDELCC     56
OREOCHROMIS_NILOTICUS            ----XXXXMTATMVQSVMLAGLVYXVFLVSYXGAQSLKPEDGASLRSGAVELPWQDELCC     56
OREOCHROMIS_MOSSAMBICUS          ----XXXXMTATMVQSVMLAGLVYXVFLVSYXGAQSLKPEDGASLRSGAVDLPWQDELCC     56
CHANNA_PUNCTATA                  ----XXXXXXXXXXXXXMLTVLMQXILLVTHAGAQGLESGGGAALHYGVLDLPWQDELCC     56
CHANNA_ARGUS                     ----MSNLNAVSRVRSTMLTVLMQXIFLVSHXGARGLEPGREASLQASVLDLPWQDRLCC     56
CHANNA_STRIATA                   ----XXXXMTTTMVPSTMLTVLMQXIFLVSHXGARAFEPEGGASLQAGVLDLPWQDELCC     56
DICENTRARCHUS_LABRAX             ----XXXXMTTTMVRSVMLTVLMHXIFLLPHXGALGLEPDEGAFLHSGPLELPWQDXXXX     56
SCOMBER_JAPONICUS                ----XXXXMNTTMVWSVMLSVLMHXIFLVSHXGALCLEPEEVASLQAGALNLPWQDELCC     56
EPINEPHELUS_COIOIDES             ----XXXXMTTTMVRSVMLTVLMHXFFLVPHXGAQCLEPENGASDHSGALDLPWQDELCC     56
SPARUS_AURATA                    ----XXXXMTTTMARSVMLTVLLHXIFLVSQXGARGLEPDDGASLYSGAVDLPWQDELCC     56
SCOPHTHALMUS_MAXIMUS             ----XXXXMTTTMSRSVMLTVLMHNILLVSHXGVWCSEPEDGASLQAGALVLPWQDELCC     56
PARALICHTHYS_OLIVACEUS           ----XXXXMTSTMFQSVMLTFLMHXNILVSPAGVRCLKPEDGASLQAXXLDLPWHDELCC     56
                                                      :    :     .                  * *:

PELTEOBAGRUS_FULVIDRACO          ALTSNGKANQSXXXXXXXXXXXXXXLSHINSPHQYQCHIQNSTNTXXXTDDSSVSQXVVSE     116
DANIO_RERIO                      DHPVQTLNSGXXXXXXXXXXXXXXXXLSEHPPEQHCQLLNATKXXXXXXXXXXXXXQQSF     116
CTENOPHARYNGODON_IDELLA          ELPFAQTVDSGXXXXXXXXXXXXXXXLSEHQPVEQCQLLNATKXXXXXXXXXXXXXLESS     116
HYPOPHTHALMICHTHYS_MOLITRIX      ELPFAQTVDSGXXXXXXXXXXXXXXXLSEHQPAEQCQLLNATKLXXXXXESSSKSXLALS     116
ONCORHYNCHUS_MYKISS              SSTPAQLHHGGEEEDTGXXXXXSAPSDTGHPHTLQCLFRNFTSTXXXXXLGPHPESELSR     116
SALMO_SALAR                      SSRPAQLHNRGKEEDRGSVGSRATPSDPGHPHTLQCLFRNFTSIXXXXXLGPHPQTEPSR     116
ORYZIAS_LATIPES                  DSPSAYLGEDRGVTNRSKXXXXTNGTISSLRHLPRCKYRRLTPEXXXXXXXSLPQXKPSE     116
HIPPOCAMPUS_ERECTUS              DSTSAQXEGEGSSTPTPEXXXXRIRSGSMLSQHPYCYLRSISTGXXXXXXXSSLNXKASG     116
CYNOGLOSSUS_SEMILAEVIS           DSXXXXLTGGGKEXXXXXXXXXDLKTNSTESNLLHCSIRSFTNTSLPVKPSGHGSXSFNT     116
TAKIFUGU_RUBRIPES                ESRPASTVEGASAPAERXXXXXPNGSNRSLPHDSQCSFKNLTSKXXXXXXXLHPXXLEFS     116
OREOCHROMIS_NILOTICUS            ESPSAHLGVEGGSANSPEXXXXANLSQSNLPHSPGCTFKSSRIDXXXXXXXSHHPXRSSG     116
OREOCHROMIS_MOSSAMBICUS          ESPSAHLGVEGGSANSPEXXXXANLSQSNLPHSPGCTFKSSRIDXXXXXXXSHHPXRSSG     116
CHANNA_PUNCTATA                  DSPSGRLTVEGGSTHAPEXXXXKSRFQSNLPHRPRCSFRSSKTKXXXXXXXSHPPXQPSG     116
CHANNA_ARGUS                     DSPSSHLTVVGGSAHPPEXXXXTNRSESNLPHHLSCNFRSSTTEXXXXXXXSHPLXQPSG     116
CHANNA_STRIATA                   DSPSGHLAVEGGSVHAPEXXXXTNGSESNLPHHLRCNFRSSTTQXXXXXXXSHPLXQPSG     116
DICENTRARCHUS_LABRAX             DSPSTHLTVDGGDMHAQEXXXXTNRPDSKLPHYSHCNFRSSTNNXXXXXXXSRPHXELSS     116
SCOMBER_JAPONICUS                DSPSAHLTVKVDGVHALEXXXXTNHSESSLPHYAHCNFRSLTATXXXXXXXSHPHXKPSG     116
EPINEPHELUS_COIOIDES             DSPSAHLTAEGDDMHAPEXXXXTNHSESNLPQHPLCSFRGSTSEXXXXXXXSHPHXEPSG     116
SPARUS_AURATA                    DSPSAHLTAEGSNAHATEXXXXTNRPDSSLPHYPCCSFXSSTVEXXXXXXXSQRHXELSA     116
SCOPHTHALMUS_MAXIMUS             DSRTAPFNEAGGDTDAPEXXXXTNRSESKLPHSPRCRFRSLTTGTRPRELSGHKSXSFNN     116
PARALICHTHYS_OLIVACEUS           DSHTTHFNIXXXXXXXPEXXXXTNRSESKLPHDPHCSFSSLTTESRPREASDIKSXSFNT     116
                                                                    *

PELTEOBAGRUS_FULVIDRACO          NIRLDIFCRLEDEQANVICLLKHQRTSATXXDASHLIVSLWRVTLESDPLTNETXXXNIT     176
DANIO_RERIO                      GSCLDILCWLEGERENLICNAKTRRAAAAXXXSTLVSVSPHQLVVQMDVHSDXXXXXEAN     176
CTENOPHARYNGODON_IDELLA          GNCLDILCWLEGERTNLICNAKSHRAAATXXXASLFTVSPQQIVLQMDILSXXXXXXDKN     176
HYPOPHTHALMICHTHYS_MOLITRIX      GNCLDILCWLEGERTNMICNAKSHRAAATXXXASLFTVSPQQIVLQMDILSXXXXXXDKN     176
ONCORHYNCHUS_MYKISS              ATCWDILCRVDETWDNVICDLKHPATSSDTSIPGSVALSLQHLTTLPDIEVXXXXXXNST     176
SALMO_SALAR                      ATCWDILCRVDETWDNVICDLKHPATSSDTSTPGSVALSLQHLATLPDSEVXXXXXXNTT     176
ORYZIAS_LATIPES                  GNCLEILCQINEKWENLTCYLQPSRXXXXKLDTGGMTFSFQQLXPDKDGTEVNSXXXXXX     176
HIPPOCAMPUS_ERECTUS              GTCLNILCRIDENWETLTCDLGVPRLPSARLNSAVVAVSLQRLMFHKNYPSVDDTSMESY     176
CYNOGLOSSUS_SEMILAEVIS           DICLDILCRLDESWEKLTCELTSNGPTSTSREAGRMAISLQRVXSQNGGSQAESXXXVSH     176
TAKIFUGU_RUBRIPES                GTCLDILCGIDEKWENVTCHLEPHALPLSLPDAGHMAVSLQRRXFQKSQSRVDSEEAASD     176
OREOCHROMIS_NILOTICUS            GTCLDILCRIDGNWRNLICDLRSRGQPSDXXXXSLMAVSLRRQLFQEDGDYPXXXXXASE     176
OREOCHROMIS_MOSSAMBICUS          GTCLDILCRIDGNWQNLICDLRSRGQPSDXXXXSLMAVSLQRQLFQEDGDYPXXXXXASE     176
CHANNA_PUNCTATA                  GICLDILCRVDENWGNITCDLQSHSQPFGNLETGRIAVSLQRLLSHEGDLWVEDESASSD     176
CHANNA_ARGUS                     GTCLDILCRIDENWENLTCDLQTQXXPSGKLDTDRMAVSLQRLLSLKDDSQVDDESTASE     176
CHANNA_STRIATA                   GTCVDILCRIDENWESVTCDLQSHDQPSGKVDTGLMAVSLQRLQSQKDDSQVDVERSASD     176
DICENTRARCHUS_LABRAX             GTCLNILCRIDENWENLTCDLQSHGPPSTTLDAGLMKVSLQRLMSQKEDTKVNDGNTASD     176
SCOMBER_JAPONICUS                GTCLDMLCRIDEKWESLTCDLQSHDPPSDTMNTALMAISLQHLLSKKKGSEVNYGNDASY     176
EPINEPHELUS_COIOIDES             GTCLDILCRIDENWENLTCDLQSHSQPSTTLDAGLMAVSLQRLLSQKDAEXVDDGNAATD     176
SPARUS_AURATA                    GTRMDILCRIDENWENFVCDLQPHGSPSTTLAADLVAVSLQRLLSQNRXXXXXXXXXETN     176
SCOPHTHALMUS_MAXIMUS             GTCLDILCRIDENWEKLTCDLTSGGRTSTALDAGLMAVSLRRVLSQKEDSLVNSGNTSPD     176
PARALICHTHYS_OLIVACEUS           GTCLDILCRIDENWEKLTCDLKSQTSTAEXXDAGLVGISFQRLLSQKDDSRVNDGKTMSD     176
                                     :::* ::    .. *                . .*  :

PELTEOBAGRUS_FULVIDRACO          TQVQCPGEDEITCFFVLQPNDVSVSLSVSGFLGGRPLQTPEMRISTDXXXLLRKPEAPFY     236
DANIO_RERIO                      STAQCVGEETAICSVSLHGGDATVSLVIIISENGTTAQSQKMQVSTYXXXELQRPGAPVN     236
CTENOPHARYNGODON_IDELLA          HTAHCAGEDTAMCSISLHGNDATVLLTISISANGTTALLPKMQISSYXXXHLRRPDPPVN     236
HYPOPHTHALMICHTHYS_MOLITRIX      HTAQCAGEDTATCSISLHGNDATVLLTISISANGTTTLSSKMQISSYXXXHLRRPDPPVN     236
ONCORHYNCHUS_MYKISS              HGTDCVGEDSITCSVALHVVSSIVVVTANVSNTTAGPXLVMLSVPPRTSQRLWKPSPPLN     236
SALMO_SALAR                      HGTDCVGEDSITCSIALHVVSSIVVVTANVSNTTAGPXLVMLSVPPXXXXRLWKPSPPLN     236
ORYZIAS_LATIPES                  NPVVCEAEESFTCSLPLHPAASFVTTVTVNLSSVVAPXPVLLIIPAXXXXRPVKPSPPVN     236
HIPPOCAMPUS_ERECTUS              ESVVCETKDSSRCSISIXGTTSFVSIVSVNISGCEARXPILLSVPAXXXXRPVKPRTPLN     236
CYNOGLOSSUS_SEMILAEVIS           NPVVCEDYDSIICSVVLDSTPTFISVEVVSISDNISSQPVYLRIPAXXXXQPGKPSPPVN     236
TAKIFUGU_RUBRIPES                PPVFCEAEDSFTCSVALDAESSFHAVVTVTIADARAPXSVLLRVPAXXXXRPVKPAPPVN     236
OREOCHROMIS_NILOTICUS            NPVVCEAQDSFMCSLTLDPTTSFVAMVTVSISDAVAPXPVLLRVPAXXXXRPEKPSPPGN     236
OREOCHROMIS_MOSSAMBICUS          NPVVCEAQDSFMCSLTLDPTTSFVAMVTVSISDAVAPXPVLLRVPAXXXXRPEKPSPPGN     236
CHANNA_PUNCTATA                  NPVICEALDSFMCSVAFDSTTSFVTMVTISISDAVAPXQVLLRIPAXXXXRPEKPGPPVN     236
CHANNA_ARGUS                     NPVVCEALDSFMCSVVLDTARSFVATVTVSISNAVAPXPVLLRIPAXXXXRPEKPGPPVN     236
CHANNA_STRIATA                   NPVICEALDSFMCSVSLDATTSCVTVVTVSISDAVAPXPVLLRIPAXXXXRPEKPGPPLN     236
DICENTRARCHUS_LABRAX             NPVVCEVKDSFMCSVALNATTSFVIVVTVSISDAEAPXPVLLRVPAXXXXRPVKPNPPVN     236
SCOMBER_JAPONICUS                NPVVCEAEDSFMCSLPLDTTTSFVTMVTVSIAEAVAPXPILLRVPAXXXXRPVKPSPPVN     236
EPINEPHELUS_COIOIDES             NPVVCEAKDSFMCSVALDTKTSFVTRVTVSISDAVAPXPVLLRIPAXXXXRPVKPSPPVN     236
SPARUS_AURATA                    NPVVCEAEDSFTCSIALDTTTSFVGMVTVSISDAVAPXPVLLRIPAXXXXRPVKPSPPVN     236
SCOPHTHALMUS_MAXIMUS             KPVACEAKDSFVCSIALDTTTSFIAVVTVSVSDAEALXPVQLRIPAXXXXRPVKPSPPVN     236
PARALICHTHYS_OLIVACEUS           NPVVCEAEESFSCSVALDTTTSFISVVTVSISNTKAPXLVQLRIPAXXXXRPVKPSPPVN     236
                                   . *   :   * . :                        : :         :*  *
```

```
PELTEOBAGRUS_FULVIDRACO        LRYNVTTEGEVMIAWNDXXSQNNKLPLYYELRYXXSPNTSLTHWEVLNVQXHPWVSLSEL    296
DANIO_RERIO                    LHYNVTTEGEVIFRWTGXXTQPDNNTINYEIQYXXSSNSLLQQWKVLKVKGRSWVALNEL    296
CTENOPHARYNGODON_IDELLA        LHYNVTTEGEVIFRWSSXXTQPDSNAINYEIRYXXSSNSSLQQWEVVKVRGRSWVPLNEL    296
HYPOPHTHALMICHTHYS_MOLITRIX    LHYNVTTEGEVIFRWSSXXTQPDSNAMNYEIRYXXSSNSSLQQWEVVKVRGRSWVPLNEL    296
ONCORHYNCHUS_MYKISS            LTHTQTTEGELILSWSDPQPHASPVQLSYEVRYNTSQSTSHPNWLHVNVSGAQWVSLTGL    296
SALMO_SALAR                    LTHTQTTEGELILSWSDPQPHASPVQLSYEVRYNTSQSTSHLNWLHVEVSGCQWVSLTGL    296
ORYZIAS_LATIPES                VTHYQTIEAELFVQWESPXPHFDAAQLRYEVRYXXNTKSDLAXWQVVSVTGEPRLSLXDL    296
HIPPOCAMPUS_ERECTUS            LSHFQTIKPELILQWDEPXKDYGSGPLRYEVRYXXSXNDTHSSWQVVSTGLERRLSLXDL    296
CYNOGLOSSUS_SEMILAEVIS         LSHVQTIQPELILMWNDAEYDPATDLMRYEVRYXXSFNRTHPDWQVVSVRGQRRVSLXDL    296
TAKIFUGU_RUBRIPES              LSHVQTIEAELILHWGDPXKDIKTDLLQYEVRYXXSPDTIHPAWQVMSVSGDTKTSLXDL    296
OREOCHROMIS_NILOTICUS          LSHIQTIEAELIVLWDDPXADFDASPLRYEVRYXXSSGTTHPAWQVVSAPGEPRVSLXDL    296
OREOCHROMIS_MOSSAMBICUS        LSHIQTIEAELIVLWDDPXADFDAGPLRYEVRYXXSSGTTHPAWQVVSAPGEPKVSLXDL    296
CHANNA_PUNCTATA                LSHVQTIEADLIVMWDDPXSDFDAGTLRYQIRYXXSSNSTHPAWHMVSTPRDPRLSLXDL    296
CHANNA_ARGUS                   LSHVQTIEAELILQWDNPXSDFDAGPLRYQVRYXXASNSTHPAWQMASASVDPRLSLXDL    296
CHANNA_STRIATA                 LSHVQTIEAELILQWDDPXSGFDAGSLRYQVRYXXSSNITHPAWQMASALTDPRLSLXDL    296
DICENTRARCHUS_LABRAX           LSHIQTIEAELILHWDDPXSNVDTGPLRYEVRYXXCSNITHPAWQVVSALAEPRLSLXDL    296
SCOMBER_JAPONICUS              LSHMQTIEAELILHWDDPXEDIDSGLLRYEVRYXXSSNTNHPAWQVVSVPAEPRMLLXDL    296
EPINEPHELUS_COIOIDES           LLHNQTIEADLILQWDDPXSDSDTGPLRYEVRYXXSSNTTHPQWQVVSAPEEPRLPLXEL    296
SPARUS_AURATA                  LSHIQTIEAELILLWDDPXPDYDTGPLRYEVRYXXSSNTTHPSWQVMPAPGAPRSSLXDL    296
SCOPHTHALMUS_MAXIMUS           LSHIQTTEAELILLWDGPXTDFDTGPLRYEVRYXXSFNTTRPAWQVMSAPAGPRLPLXDL    296
PARALICHTHYS_OLIVACEUS         LSHIQTIEAELILLWDDPXSDSSTGPLRYEVRYXXSFTTTRPVWQVVSXSAEPRLSLXDL    296
                               : :  * : :::. *             : *:::*          *           *  *

PELTEOBAGRUS_FULVIDRACO        TSGVRYTVQVRCKSLHHLHNWSEWSNWSQPFFLTLDXVSYIPAEVFTRPGAEVTVYGVVH    356
DANIO_RERIO                    SSDIRYTVQVRCXXXQNXXHLGYWSEWSQPFYFKLDXVSYIPAEVFTTQESEVTVYSVFH    356
CTENOPHARYNGODON_IDELLA        SSGIRYTVQVRCXXXQNXXNFNYWSEWSQPFYFTLDXVSYIPAEVFTTPGSEVTVYAVFH    356
HYPOPHTHALMICHTHYS_MOLITRIX    SSGIRNTVQVRCXXXQNXXNFNYWSEWSQPFYFTLDXVSYIPAEVFTTPGSEVTVYAVFH    356
ONCORHYNCHUS_MYKISS            GPGLQYTLQIRSXXXHHPALPHLWSDWSQQHRIRLENVTYLPERVVASFGDSVTVYCVCN    356
SALMO_SALAR                    RPGLHYTVQIRSXXXHHPARPHLWSDWSQQHRIRLENVTYLPERVVASFGDSVTVYCVFN    356
ORYZIAS_LATIPES                QPEQEYTFQVRCXXXSRLDEPPLWSEWSAPYKFYQYIVTYIPEKMVARAGESVTVYCLFN    356
HIPPOCAMPUS_ERECTUS            EPEVNYTMQVHCXXXSDLRDPPLWSNWSEPYHIYLGRVSYIPEKVLAHPGENISLYCVFN    356
CYNOGLOSSUS_SEMILAEVIS         QHSINYTTQVRRXXXSRLGESSLWSDWSESYYIYIKTVSYIPEIGLTRPGENITVYCVFN    356
TAKIFUGU_RUBRIPES              KACVNYTVQVRRXXXSSRSDPPLWSGWSESHHIFLDEVSYIPEKVVVKAGENVTVYCVFN    356
OREOCHROMIS_NILOTICUS          KPELKYSVQVRCXXXSGPEEPPLWSEWSEPHHIRLDTVSYIPKIVVAKPGENVTVYCVFN    356
OREOCHROMIS_MOSSAMBICUS        KPELKYSVQVRCXXXSGPEEPPLWSEWSEPHHIRLDTVSYIPKIVVARPGENVTVYCVFN    356
CHANNA_PUNCTATA                KPRLNYTIQVRCXXXSGLDDPPLWSNWSEPYHIYLDTVSYIPEKVIARPGEYVDVYCVFN    356
CHANNA_ARGUS                   KPSLNYTIQVRCXXXSGLDDPPLWSDWSKPYHIYLNTISYIPQNVVARVGENVTVYCVFN    356
CHANNA_STRIATA                 KPRLNYTIQVRGXXXSRLDNPPLWSDWSEPHHIYLDTVSYIPGKVVARPGENVTVYCVFN    356
DICENTRARCHUS_LABRAX           KPRLNYTIQVRCXXXSSLDKPPLWSDWSEPHHINLDMVSYIPEKVVVRPGENVTVYCVFN    356
SCOMBER_JAPONICUS              KPRLNYTIQVRCXXXSGLDEPPLWSDWSEPYHIYLDTVSYIPEKVVARPGENVTVYCVFN    356
EPINEPHELUS_COIOIDES           KPRLNYTIQVRCXXXSGLENPPVWSDWSEPYHIYLDTVSYIPEKVVARPGDNVTVYCVFN    356
SPARUS_AURATA                  KAKLNYTVQVRCXXXSSLDRPPLWSDWSEPHHIYLDTASYIPEKVVVRPGENVTVYCVFN    356
SCOPHTHALMUS_MAXIMUS           KPRLNYTIQVRCXXXSGPGEPPLWSDWSESHHIYLDTVSYIPEKVVARPGENVTVYCVFN    356
PARALICHTHYS_OLIVACEUS         RPTLNYTIQVRCXXXSGLDEPPLWSDWSESHHIYLHTVSYIPEKVVTHAGEEVTVYCVFN    356
                                    . : *::             ** **  . :      :*:*   ..      : :* : :

PELTEOBAGRUS_FULVIDRACO        NHSRTASKAVWMLNGLVXLPESQYQTINERVSAVTIRSKEPGXFDTLLCXXCYPFEQSYK    416
DANIO_RERIO                    NRSWSASKAVWFLNGKMKIPESQYRVINEQVSTVTLKMDKAGXFDTLMCXXCLTLGEKSM    416
CTENOPHARYNGODON_IDELLA        NRSWSASKAVWMLNGQVKIPESQYSVINDQVSAVTMKVDEPGXFDTLMCXXCHQWGERFK    416
HYPOPHTHALMICHTHYS_MOLITRIX    NRSWSASKAVWFLNGQVNIPESQYSVINDQVSAVTVKLDEPGXFDTLMCXXCLGXGEKFK    416
ONCORHYNCHUS_MYKISS            DLRVNASTMVWILNSRDRLPKSQYAAVNDRVSKITVRPSEQRLSGTLHCXXCQPFGEMYS    416
SALMO_SALAR                    DLRVNASTTVWILNSRDRLPKSQYTAVNDRVSKITVRPSEQRLSDTLHCXXCQPLGETYS    416
ORYZIAS_LATIPES                NRSMNASEAVWKLNFHQLLHSSQXXSVSGRVSKITMRASESRMYDLLTCXXTQKAXXXXX    416
HIPPOCAMPUS_ERECTUS            DHSINANSAVWMRNLEQPLSSSQYHSVNQRVSHITIQPSDSGMYELLQCRATKEWXXXXX    416
CYNOGLOSSUS_SEMILAEVIS         DPSIDASMAVWTLNMNQQLPQSQYHAVNRWVSQITLQPSQSRMYDLLQCXXTQEWXXXXX    416
TAKIFUGU_RUBRIPES              DHNFNASTALWTLNFDQELDYSLYHPINQWVSQVTMRPSETGMYDLLQCXXTKKRXXXXX    416
OREOCHROMIS_NILOTICUS          DHRMNASMAVWKLNFKPPLQPTLYHPVNQWVSKITVRPSENQMYDLLSCXXTEGWXXXXX    416
OREOCHROMIS_MOSSAMBICUS        DHRMNASMAVWKLNFKPPLQPTLYHPVNQWVSKITVRPSENQMYDLLSCXXTEGWXXXXX    416
CHANNA_PUNCTATA                DHSINASSAVWMLNFQEPLHRNQYHPVNQWVSQVTVRPSESRMYDLLQCXXTQEWXXXXX    416
CHANNA_ARGUS                   DHSVNASTATWMLNLHEQLPRSQYHPVNQWVSKITLRPSERQMYALLQCXXTQKWXXXXX    416
CHANNA_STRIATA                 DHSVNASTAVWMRNLQEPLHRSQYHPVNQWVSQITVRPSETRMYDILQCXXTQQWXXXXX    416
DICENTRARCHUS_LABRAX           DHNINASTAMWMLNFQEQLDHSQYHPVSQWVSQITVRPSETRLYDLLQCXXXTKWXXXXX    416
SCOMBER_JAPONICUS              DHSINASTAMWMLNFQQPLHRSQYHPVNQWVSQITVRPSETRMYDLLQCXXTQEWXXXXX    416
EPINEPHELUS_COIOIDES           DHSINASTAMWMRNFQQRLHSSQXXPVNQWVSQITVRPSESQMYDLLQCXXTQEWXXXXX    416
SPARUS_AURATA                  DHNINASTAMWMLNFQQPLNRSQYHPVNQWVSQITVRPSETQMYDLLQCXXTQEWXXXXX    416
SCOPHTHALMUS_MAXIMUS           DRGINASAAVWVLNFQQPLPRSQYHPVNQRVSQITVRPSETRMYDLLQCXXIQEWXXXXX    416
PARALICHTHYS_OLIVACEUS         DHSVNASAAIWVLNFQQTLHRSQYHPVNQWVSQITVRPSETRMYDLLQCXXTKEWXXXXX    416
                               :    *.   *  *     :  .     :.  ** :*:: ..        * *

PELTEOBAGRUS_FULVIDRACO        CSIAYTKVYYEGFFDANITCTTEQHSSVDTMTCKWNKSAWAVIRFLYRRYRRTCDEIQYE    476
DANIO_RERIO                    CSIAYAKIYTEGRFNANITCESEXYSYVDTMICKWNQSNWAQARLLYRQYRSKCXXXXXX    476
CTENOPHARYNGODON_IDELLA        CIIAYAKIYTEGMFNADITCQSKXNSEVDTMSCEWNKSAWAQVRLLYRQYTSMCETIXXS    476
HYPOPHTHALMICHTHYS_MOLITRIX    CNIAYTKIYTEGMFNADITCQSKXNSEVDTMNCVWNKSAWAQVRLLYRQYTSMCETIXXS    476
ONCORHYNCHUS_MYKISS            CNYHYSTIYIKDPXVIDISCVTXXSGDLDSMTCRWNNLPIGGINFMSRVADLSCXXXXXD    476
SALMO_SALAR                    CNYRYSTIYIKDPXVIDISCVTXXNGDLDSMTCRWNNLPIGGINFMSRVADLSCXXXXXD    476
ORYZIAS_LATIPES                XALPYSQISIEGAXSLDIRCETXXNGNMDTMECSWNSTQWLSFNLQHKWTHMSCXXXXXE    476
HIPPOCAMPUS_ERECTUS            XPIPYSIIYVEGAXSIDIKCETXXSGDIDVMDCSWNNTWWIKPKFQYAWTDLPCXXXXXD    476
CYNOGLOSSUS_SEMILAEVIS         XSLPCSKIYIQGSXSIDINCVTXXSGGIDAMECRWNCKQWRKTEFQYRWAYLSCXXXXXK    476
TAKIFUGU_RUBRIPES              XMIAYSQVYYEGAXSISISCETXXNGEIDAMDCRWNSTQWLNPNFRTRWADLSCXXXXXD    476
OREOCHROMIS_NILOTICUS          XSIPYSQIYVEGAXDIDIKCVTXXNGDIDAMDCSWTHKQLTKLRFRSKWADLSCXXXXXD    476
OREOCHROMIS_MOSSAMBICUS        XSIPYSQIYVEGAXDIDIKCVTXXNGDIDAMDCSWTHKQLTKLRFRSKWADLSCXXXXXD    476
CHANNA_PUNCTATA                XTIPYSQIYVEGAXSIDIKCETXXNGDIDAMDCSWKNTQWTKPTFQSRWADLPCXXXXXD    476
CHANNA_ARGUS                   XTIPYSEIYVEGAXSIDIKCETXXNGDIDAMDCSWKNTKWPKPLFQSKWADLPCXXXXXD    476
CHANNA_STRIATA                 XTIPYSQIYVEGAXSIEIKCETXXNGDIDAMQCSWETKQWTKLEFQSRWADLPCXXXXXD    476
DICENTRARCHUS_LABRAX           XTISYSQIYVEGAXSININCETXXNGDIDAMDCRWKNSVWTQLKLKSWWANLPCXXXXXD    476
SCOMBER_JAPONICUS              XTIPYSQIYVEGAXSIDIKCETXXNGDIDAMDCSWKNTQWTKPKFLSRWADLPCXXXXXD    476
EPINEPHELUS_COIOIDES           XTIPYSQIYVQGAXSIDITCETXXNGDIDAMTCSWKSTQWTRLKFRSRWADLQCXXXXXD    476
SPARUS_AURATA                  XTIPYSQIYVEGAXSIDINCETXXNGDIDAMDCSWKNTQWTKLQFRSRWADLPCXXXXXD    476
SCOPHTHALMUS_MAXIMUS           XTLPYSQIYVEGAXSIDINCEAXXNGDIDAMDCSWKNTQWTKPTFRSRWADLPCXXXXXD    476
PARALICHTHYS_OLIVACEUS         XTNPYSQIYVEGAXSIDINCVTXXNGDIDSMDCSWKNTQWTEPELRSRWADLPCXXXXXD    476
                                    : :  :.     .* * :    . :* * * *            :         *
```

```
PELTEOBAGRUS_FULVIDRACO        EGTLTQAQGDPMVDVEEXXCTAGAGDYXYQCTLQDLSLISCYKLWLVVEDGYNKVRSLPV    536
DANIO_RERIO                    XXXXTEEAEEDTSLVKEXXCPSKAGDHXRQCTLSQISMIFCYKFWLEVEGGRGXXQSFPV    536
CTENOPHARYNGODON_IDELLA        EMEGTEEAEENMSLVKEXXCPSGAGDHXRECTLSNLSLFSCYKIWLEVEGGRGKVRSFPV    536
HYPOPHTHALMICHTHYS_MOLITRIX    KMEGTEEAEENMSLVKEXXCPSGAGDHXRECTLSNLSLFSCYKIWLEVEGGHGKVRSFPV    536
ONCORHYNCHUS_MYKISS            VMEEAERVGVPVGVVRQAKCESSGYRGVKSCNLQPIRVTSCYKLWMEAKTDXNSTRSHPV    536
SALMO_SALAR                    VMEEAERVGVPVGVVRQAKCESSGYRGVKSCNLQPIRVTSCYKLWMEAKTDXNSMRSHPV    536
ORYZIAS_LATIPES                RMKEKEEAGDNVGKIVDAXCYSIKPXXXRTCTFKPLRFGXCYKLWLELRTDSGSVRSKPI    536
HIPPOCAMPUS_ERECTUS            VMEEREKKGEEVGELGPDXCLPVRSKQXQACTIQDIRRNXCYKLWLEMLSHQGPFRSKHI    536
CYNOGLOSSUS_SEMILAEVIS         EMEEMDRAGENVGETGPPXCLQVTSQEXNMCTIQTLRMSXCYKMWLEMPSESGPISSKPI    536
TAKIFUGU_RUBRIPES              VMEERERAGDNVGHEGPSXCLQVDSRKXRLCTIQPLRTNXCYKLWLEVSSHLGLIRSKPV    536
OREOCHROMIS_NILOTICUS          VMEERERAGENLGEMGPAXCMEGGQXXXETCTIHPLRMNXCYKLWLELPSQLGPIRSKPV    536
OREOCHROMIS_MOSSAMBICUS        VMEESERAGENLGEMGPAXCMEGGQXXXETCTIHPLRMNXCYKLWLELPSQLGPIRSKPV    536
CHANNA_PUNCTATA                VMEERERAGETVGEMAPAXCLPVGSRQXKSCTIQPLRMNXCYKLWLEVPSRLGPIRSRPI    536
CHANNA_ARGUS                   VMEERERAGEKVGEMGPAXCXXXRSTQXESCTIQPLRMNXCYKLWLELPLPLGHVRSRPI    536
CHANNA_STRIATA                 VMEERERAGEEVGEMGPAXCLAIGSRHXESCIIHPLRMNXCYKLWLELPSRLGPVRSRPI    536
DICENTRARCHUS_LABRAX           VMEERERAGENVGEMGPDXCLQVRSQQXKTCHIQPLRMNXCYKLWLEMPSRLGPIRSKPI    536
SCOMBER_JAPONICUS              VMEERERAGESVGEMGPVXCLSQXXXXXKSCTIQPLRMNXCYKLWLEVPSRLGPIWSKPV    536
EPINEPHELUS_COIOIDES           VMEERERAGEKVGEMGPSXCLQVRSKQXKTCTIQPLRMNXCYKLWLEVPSRLGPIRSKPI    536
SPARUS_AURATA                  VMEERERAGEKVGEMGPAXCPQVLSRQXKSCTIQPLRMNXCYKLWLEVPSRLGPIRSKPV    536
SCOPHTHALMUS_MAXIMUS           VMEERDRAGESVGQMGPVXCLQVRSRQXKSCTIQPLRMNXCYKLWLEVPSRLGPIRSKPI    536
PARALICHTHYS_OLIVACEUS         VMEERDRAGEEVGEMGPAXCLQVRSKQXKSCIIQPLRMNXCYKLWLEVASRLGPIRSKPV    536
                                    :             *          * :  :    ***:*:      .   *  :

PELTEOBAGRUS_FULVIDRACO        FVSPIDCVKPSPPSELKAVTLPNKTLSATWKRPYLPAYDLQYELRYVSMHGXMVDLKWKV    596
DANIO_RERIO                    YVTPIDYVKPSPPFDLEAITLPSKTLSVRWKRPSLPVYGMQYELQFKALAGXMANTQWKV    596
CTENOPHARYNGODON_IDELLA        YVAPIDYVKPSPPSVLEAITLPNKTLSVKWGRPHLPVYDMQYELRFVTLRGXMANTQWKV    596
HYPOPHTHALMICHTHYS_MOLITRIX    YVAPIDYVKPSPPSVLEAITLPNKTLSVKWRRPHLPVYDMQYELRFVALRGXMANTQWKV    596
ONCORHYNCHUS_MYKISS            YITPMDHVKPHPPSGLEAVSMPSGVLKLAWVPPELPIYDMQYQVRYALSTGXKAHPFWQV    596
SALMO_SALAR                    YITPMDHVKPHPPSGLEAVSMPSGVLKLAWVPPELPIYDMQYQVRYALSTGXRAHPFWQV    596
ORYZIAS_LATIPES                YLSSKGQVKPYTPTNVKAVTLRSGVLSVTWEPPSLPIDGLQYELQYHPLSTXXVKEEWKV    596
HIPPOCAMPUS_ERECTUS            YISPLDHVKPHKPSNVKAVNLQSGILRITWEPLPLHVQGLQCQFRYHSPSAIKAQPEWKL    596
CYNOGLOSSUS_SEMILAEVIS         YLSPLDHVKPQPPTNVKAVSLARGVLSVTWECPLLPVEGLQCQFQYHSPSAVRAQPEWKV    596
TAKIFUGU_RUBRIPES              YLTPNDHVKPHTPTDVKAVSRSGGVLNVTWKRPYLPVEXVQCQFRYHSPSADHPKPDWKV    596
OREOCHROMIS_NILOTICUS          YLSPVDHVKPHAPANVKAVSHSSGVLEVTWQAPPLPADGLQCQFQYHSPSTVSPRPRWKL    596
OREOCHROMIS_MOSSAMBICUS        YLSPVDHVKPHAPANVKAVSHSSGVLEVTWQAPPLPADGLQCQFQYHSPSTVSPRPKWKL    596
CHANNA_PUNCTATA                YLSPIDYVKPHTPTNVKAESLSSGVLVVSWEPPPLPVEGLQCQFRYHSPSTARAQQEWKV    596
CHANNA_ARGUS                   YLSPIDYVKPHSPTNVKAESLRSGVLMVSWEPPSLPVEGLQCQFRYHSPSTARAQPEWKV    596
CHANNA_STRIATA                 YLSPIDNVKPHMPTDLKAESLSTGVLLVSWEPPSLPVEGVQCQFQYHSPSTARAQPEWKV    596
DICENTRARCHUS_LABRAX           YLSPIDHVKPHTPTNVKAGSQSSGVLTVTWEPPSLPVDGLQCQFRYHSPSAVRAQPEWKV    596
SCOMBER_JAPONICUS              YLSPIDHVKPHTPTNVRAVSRSSGVLTITWEPPSLPVEGLQCQFRLHSLSTVRAQPEWKV    596
EPINEPHELUS_COIOIDES           YLSPIDHVKPHSPTNVKAVSRSSGVLLISWEPPSLPVEGLQCQFRYHSPSAVRAQPEWKI    596
SPARUS_AURATA                  YLSPIDHVKPHTATNVKAVSRSSGVLAVTWEPPSLPVEGLQCQFRYHSPSTVRAQPEWKV    596
SCOPHTHALMUS_MAXIMUS           YLSPIDHVKPHTPTNVKAVSRSSGVLAVTWEPPSLPVDGLQCQFQYHSPSMVRAQPEWKV    596
PARALICHTHYS_OLIVACEUS         YLSPIDHVKPHTPTNVEAVSRSSGALLVTWKPPFLPVEGLQCQLRYHSLSMVRAQPQWKV    596
                               :::  . ***     :.* .     *   *    *    :* :.:            *::

PELTEOBAGRUS_FULVIDRACO        FGSLLESRATFTVLDPCIQYQVQVRCRRLNGPGYWSDWSYTHASSVYNVKAPEMGPDFWR    656
DANIO_RERIO                    IGPLLEPQAEIQLEESCVHFKVEVRCKDVNDTGYWSDWSNSHISTVFNLKAPEMGPDFWR    656
CTENOPHARYNGODON_IDELLA        IGSLLEPQAEVPLEDSCVQFKVEVRCRRLNGSGYWSDWSRSHTSIVYNRKAPEMGPDFWR    656
HYPOPHTHALMICHTHYS_MOLITRIX    IGSLLEPQAEIPLEDSCVQFKVEVCCRRLNGPGYWSDWSRSHTSIVYNRKAPEMGPDFWR    656
ONCORHYNCHUS_MYKISS            LALQTESWAEVLEPDVCGVYNVQVRCMHINGSGTWSDWSHLLYTTTHNSRAPDQGPDFWR    656
SALMO_SALAR                    LALQTESWAEVLEPDVCGVYNVQVRCRHINGSGTWSDWSHLLYTTTHNSRAPERGPDFWR    656
ORYZIAS_LATIPES                QRSKQPPPMTVQVPEMCRSYVVQVRCMHIAGKGYWSEWSDLIYSTPNNSKAPERGPDFWR    656
HIPPOCAMPUS_ERECTUS            SNPVWVPSAEIPVPDMCQVYAVQVSCKPANRTGYWSDWSDSVYSVPQNSQAPEHGPDFWR    656
CYNOGLOSSUS_SEMILAEVIS         QSPVRDPWSEVVVPHMCRVYVVQVRCMPANGSGYWSEWSETVYSTPQNSRAPERGPDFWR    656
TAKIFUGU_RUBRIPES              QAIVREPWAEVNVSDVCRVFVVQVRCMHISGAGYWSEWSPSVYSSPQNSRAPERGPNFWR    656
OREOCHROMIS_NILOTICUS          QDPVRVPWAEVAVPDMCRVYVVQVRCKHTNGTGYWSDWSESVYSTPQNSRAPERGPDFWR    656
OREOCHROMIS_MOSSAMBICUS        QDPVRVPWAEVAVPDMCRVYVVQVRCKHTNGTGYWSDWSESVYSTPQNSRAPERGPDFWR    656
CHANNA_PUNCTATA                QSPVRAPQAEISVPDMCRVYVVQVRCMHVNGTGYWSDWTDSVYSTPQNSRAPERGPDFWR    656
CHANNA_ARGUS                   QSPVRVPWAKVVVPDMCRVYVVQTRCMHVNGTGYWSDWSDSVYATPQNSRAPERGPDFWR    656
CHANNA_STRIATA                 QSPVRVSWAEVVVPDMCRVYVVQARCMHINGTGYWSDWSKSVYATPQNSRAPERGPDFWR    656
DICENTRARCHUS_LABRAX           QSPVRVPWAEVAVADMCREYAVQVRCMPTNGTGYWSDWSNSVYSTPQNSRAPERGPDFWR    656
SCOMBER_JAPONICUS              HNPVRVQWAEVAVPDMCRVYVVQVRCMHTNGTGYWSDWSDSIYSTPQNSRAPDRGPDFWR    656
EPINEPHELUS_COIOIDES           QSPVRVPWAEVLVPDMCRVYVVQVRCMHTSGTGHWSEWSDSVYSTPQNSRAPERGPDFWR    656
SPARUS_AURATA                  QSPVRVPLGEVTVSDMCRVYVVQVRCMHTNGTGYWSEWSDSVYSTPQNSRAPERGPDFWR    656
SCOPHTHALMUS_MAXIMUS           QSPVRVPRAEVVVPDMCRVYVVRVRCMHTNGTGYWSEWSDSVYSAPQNSRAPERGPDFWR    656
PARALICHTHYS_OLIVACEUS         QSPVRVALSEVAVPDMCQVYVVQVRCMHTNGTGYWSEWSDSVYSAPQNSRAPERGPNFWR    656
                                         .   . *  : *.. *      * **:*:    :   * :**: **:***

PELTEOBAGRUS_FULVIDRACO        IIQETPEPYXTNVTLLFKXXPLPEVEAAIXCVQGLVVVHQTSGGNVWSDDIIAPSSFYTF    716
DANIO_RERIO                    ILQEDPTRNVTNVTLIFKXXQPILAGDPNSCVEGLVIKHQASGGVMWSNEXTTLARFHSF    716
CTENOPHARYNGODON_IDELLA        IIQEDPVRSVTNVTLIFKXXQPVLAGDPYSCVEGLVIKHQASGGAVWSNEXTTLTQFHSF    716
HYPOPHTHALMICHTHYS_MOLITRIX    IIQEDPVRSVTNVTLIFKXXQPVLAGDPYSCVEGLVIKHQASGGAVWSHEXTTLAQFHSF    716
ONCORHYNCHUS_MYKISS            VFQEDPASMQTNVTLLFEXXHSPIVEPTYXCVEELVVQHQDSGGTVTEERXIGLVSSYSF    716
SALMO_SALAR                    VFQEDPASTQTNVTLLFEXXHSPIVEPTYXCVEELVVQHQDSGGTVTEERXIGLVSSYSF    716
ORYZIAS_LATIPES                IRQDNQHINKSNITLLFEXXHFPGTWNSYXCVDGFIVQHEASNRSVVRKQXINLGSSYSF    716
HIPPOCAMPUS_ERECTUS            LLENDQLTNSTNVTLLITXXPLPKMSRSYXCIDGYIVQQQTSNGIVKREKXIELLSSYSF    716
CYNOGLOSSUS_SEMILAEVIS         VLQDDPKTNQTNVTLLFEXXDLPTSRHSYXCVDGFKIHHQTSTGPVISRSXVELVSSYSF    716
TAKIFUGU_RUBRIPES              FLQDDPHRKQTNVTLLFKXXDLQTSGQPYXCVEGFLVKRLGSTGPVXQEPXILMQSSYSF    716
OREOCHROMIS_NILOTICUS          IRQDDPHGNQSNITLLFEXXNFPPSGNSYXCVDGFVVQRRSSSGSVLRETXIELMSSYSF    716
OREOCHROMIS_MOSSAMBICUS        IRQDDPHGNQSNITLLFEXXNFPPSGNSYXCVDGFVVQRRSSSGSVLRETXIELMSSYSF    716
CHANNA_PUNCTATA                ILQDEPYRNQTNVTLLFEXXQMPISGPSYXCVDGFIIQLQASSGTVMREQXIEVVSSYSF    716
CHANNA_ARGUS                   IVQDDPSRNQTNVTLLFEXXQIPISGHSYXCVDGFVVQHQASNGAVMRELXIEVVSSYSF    716
CHANNA_STRIATA                 ILQDDPYRNQSNVTLLFEXXQIPISGHSYXCVDGFIIQHQTSSGAVMREQXVEVVSSYSF    716
DICENTRARCHUS_LABRAX           ILQDDPYRSQTNVTLLFKXXNLQMSGQSYXCVDGFIVQRQTSSGSVIREKXIQLVSSYSF    716
SCOMBER_JAPONICUS              VLQDDPYRNQTNVTLLFQXXPLLTSGHSYXCVDGFIVQHQSSSGTVIRERXIELVSSYSF    716
EPINEPHELUS_COIOIDES           VLQDDPYRNQTNVTLLFEQHHLQLSARSYXCIDGFIVQYQALSGSVMREQXIELASSFSF    716
SPARUS_AURATA                  TLEDDPYRNQTNVSLLFEXXRLQISGHSYXCVDGFIVQRQTSSGSVTREQXIDLASSYSF    716
SCOPHTHALMUS_MAXIMUS           VLQGDPDRNQTNVTLLFEXXDLPVSGHSSHCVDGFIVQHQTSGGAVTREWXIEPASSYSF    716
PARALICHTHYS_OLIVACEUS         ILQDDPYRNQTNVTLLFKXXDLPVSGRSYXCVDGFIVQHQTSSGIVTRNSXIEPASSYSF    716
                                 :       :*::*::             *::   :        :           .:*
```

```
PELTEOBAGRUS_FULVIDRACO         QWREEVHSITVMSRNSLGSSAENSNMTLVRQPKRQCVRWFHVTXANASCVFLSWSLLSEQ      776
DANIO_RERIO                     QWRKEAHTVTVMSRNALGISTWNRNITLLRQAKRRCVRSFSAVXANVSCVHLSWSLLSDQ      776
CTENOPHARYNGODON_IDELLA         QWRKEAHTVTVMSRNTLGISTRNSNMTLLHQPKRRCVRLFSGVXANASCVHLSWSLLSDQ      776
HYPOPHTHALMICHTHYS_MOLITRIX     QWRKEAHTITVMSRNTLGISTRNSNMTLLHQPKRRCVRSFSGVXANASCVHLSWSLLSDQ      776
ONCORHYNCHUS_MYKISS             EWRGEVHSVTVKAQNSQGSSTRNTHMTLDRHPKRQCVRSFSASRVNSSCVVLLWSLQPNS      776
SALMO_SALAR                     EWRKEVHSVTVKAQNSQGSSTRNTHMTLDRHPKRQCVRLFSASRVNSSCVVLLWSLQPNS      776
ORYZIAS_LATIPES                 EWNQEPQTVTVEAYNSLGNSTNNKNMTLGKTSRRKAVHSVHALVLNSTHVSLSWSLLNDG      776
HIPPOCAMPUS_ERECTUS             EWKQDLQSVTVEAYNSLGSSRKNMIMTLDRRPKRRSVRSFRVLLINGTCVSLHWSLVDNS      776
CYNOGLOSSUS_SEMILAEVIS          EWNQEVQTVTVEAYNSLGSSTNNINMTLEKPPKRHCVQNFHVLVVNSTCVSLSWSLLENS      776
TAKIFUGU_RUBRIPES               EWNQMPQTVTVEAFNSLGSSSDNINMTLEKSPKRRCVHHFSVTVINSTCVSLSWTLIDKS      776
OREOCHROMIS_NILOTICUS           EWNQELQTVTVEAYNSLGNSRDNINMTLERQPKGHCVRSFHVLLINGTCVSLSWSLLENS      776
OREOCHROMIS_MOSSAMBICUS         EWNQELQTVTVEAYNSLGNSRDNINMTLERQPKGHCVRSFHVLLINGTCVSLSWSLLENS      776
CHANNA_PUNCTATA                 EWNQDLHTVTVEAYNNIGSSTNNINMTLENQPKRRCVQSFRVLVINSTCVSLSWSLLDNS      776
CHANNA_ARGUS                    DWNQELQTVTVEAYNSLGSSTNNINMTLESHPKRRCVQSFRVLVINSTCVSLSWSLLDNS      776
CHANNA_STRIATA                  EWNQDLHTVTVEAYNSLGSSTNNINMTLESQPKRRSVQSFRVLVINSTCVSLSWSLVENT      776
DICENTRARCHUS_LABRAX            EWNQVLQTVTVEAYNSLGSSANNINMTLERQPKRHCVRSFSVRVINSTCVSLSWSLLDNS      776
SCOMBER_JAPONICUS               EWNQEVQTVTVEAYNSLGSSANNINMTLGRQPKRRSVRAFRALVINSTCVSLSWSLLDNS      776
EPINEPHELUS_COIOIDES            EWNQVPQTVTVEAYNNLGSSANNFNMTLERQPKRRSVRSFSVLVINSTCVSLSWTLLDNN      776
SPARUS_AURATA                   EWNQMLQTVTVEAYNSLGSSAHNINMTLERQPKRRCVSSFSVLVINSTCVSLSWTLLDNN      776
SCOPHTHALMUS_MAXIMUS            EWNQELQTVTVEAYNSLGSSANNINMTLERQPKRHCVRSFRVLLTNSTCVSLSWSLLDNS      776
PARALICHTHYS_OLIVACEUS          EWNQEPQTVSVKAYNSRGSSANNINMTLEQKPKRRCVSSFSVSVINSTSVSLSWILLDNR      776
                                :*.   ::::* : *  * *  *  :**    : :.*  .     * : * * * *  .

PELTEOBAGRUS_FULVIDRACO         PSLLSFVLEWQXEQSGVSSQGWASDGRVEWLRVASTARDLQLCRPFYGTEXXEFKLYPVF      836
DANIO_RERIO                     PVPQSFVIEWLXDLNKDPEKDVSLTERIQWVRVESRSRDLSLCRRFYGSEXXEFTLYPVF      836
CTENOPHARYNGODON_IDELLA         PVPQSFVIEWIXDLNKDPEQDVSLTERLQWVRVHSASRDLSLCRRFYGSEXXEFTLYPVF      836
HYPOPHTHALMICHTHYS_MOLITRIX     PVPQSFVIEWLXDLNKDPEQDVSLTERLQWVRVQSASRDLSLCRRFYGSEXXEFTLYPVF      836
ONCORHYNCHUS_MYKISS             SVPWSLVVEWSXGQNHQDRPDQTPESRERWTRFPPTDKLLYLYGHFYDTEXYEFILYPVF      836
SALMO_SALAR                     SVPWSLVVEWSXGQNHQDRPDQTSESRERWTRFPPTDKLLYLYGHFYDTDEYEFILYPVF      836
ORYZIAS_LATIPES                 IVPLFMVVQWSXXXXXXXXXESSGLSGLKWARLPYSNHVVYIKGSFSRSEDYSFHLYPVF      836
HIPPOCAMPUS_ERECTUS             STPLFMVVQWAXPQRQDGFDLSKAHSGRTWARLPSTDCSVHLTGDFYASEEYGFYLYPVF      836
CYNOGLOSSUS_SEMILAEVIS          SVPLFMVVQWFXPHWQHXXXHHKVQSRKTWARLPYTAGPVYLTGEFFGSEEYGFYLYPVF      836
TAKIFUGU_RUBRIPES               SPPIFMVVQWSXLLWKQDSGRPRGQSTDTWVRLPYTDGPTYLGGHFFGSEDYGFYLYPVF      836
OREOCHROMIS_NILOTICUS           SVPLFMVVEWLXPHKQQDXXXXSGPRAETWTRLRYTDHPVYLRGDFFASEEYGFFLYPVF      836
OREOCHROMIS_MOSSAMBICUS         SVPLFMVVEWLXPHKQQDXXXXSGPRAETWTRLRYTDHPVYLRGDFFASEEYGFFLYPVF      836
CHANNA_PUNCTATA                 SAPLFMVVQWLXPQTQQDSDLHKRHNGGTWARLSYTDRPVYLRGDFFASEEYVFHLYPVF      836
CHANNA_ARGUS                    SAPLFMVVQWLXAQRQRDSDLHKGQNGETWTRLSYTDHPVYLRGDFFASEEYGFYLYPVF      836
CHANNA_STRIATA                  SAPLFMVVQWLXPQRQQDXXLHRSPNGESWARLPYADHPFYLRGDFFASEGYVFYLYPVF      836
DICENTRARCHUS_LABRAX            SVPMFLVVQWSXPQRQPDSGYHKGQSGETWARLPYTDRPTYLRGDFFGSEECGFSLYPVF      836
SCOMBER_JAPONICUS               SVPLFMVVQWLXPQRQQDPHHHKGLSGEPWVRVPYTDRPVYLRGDFFGSEEYGFYLYPVF      836
EPINEPHELUS_COIOIDES            SVPLFMVVQWSXPHKQQDSDHHKGRIGETWARLPYTDHPIYLKGDFFGSEECGFYLYPVF      836
SPARUS_AURATA                   SVPLSMVVQWSXXXXXXXXXHHKGQSGHMWARLPYTDRPIYLRGDFFGSEESGFYLYPVF      836
SCOPHTHALMUS_MAXIMUS            SAPLSMVVQWSXPHRQPESGRPRAQGGETWARLPYVDHPVYLRGDFFSSEDYGFYLHPVF      836
PARALICHTHYS_OLIVACEUS          SVPVSMVVQWTVPQRQQGSDHQKAYSGETWVRLPYTDCPVHLRGDFFGSEEYGFHLYPVF      836
                                     :*::*                   * *.        :   *  ::   * *:***

PELTEOBAGRUS_FULVIDRACO         VDGEGEAVRCTAVXRSDPXXXXXXXXXXXXXXXXXXXXXXXXXXXXXXXXXXXXXXXXXX      896
DANIO_RERIO                     ADGEGEPARYTATXRGDPXXXXXXXXXXXXXXXXXXXXXXXXXXXXXXXXXXXXXXXXXX      896
CTENOPHARYNGODON_IDELLA         ADGEGEPVRYTATXRSDPXXXXXXXXXXXXXXXXXXXXXXXXXXXXXXXXXXXXXXXXXX      896
HYPOPHTHALMICHTHYS_MOLITRIX     ADGEGEPVRYTATXRSDPXXXXXXXXXXXXXXXXXXXXXXXXXXXXXXXXXXXXXXXXXX      896
ONCORHYNCHUS_MYKISS             ADGEGEPVYTKVFXRGSDAGPAXXXXXXXXXXXXXXXXXXXXXXXXXXXXXXXXXXXXXX      896
SALMO_SALAR                     ADGEGEPVYTKVFXRGGDAGPXXXXXXXXXXXXXXXXXXXXXXXXXXXXXXXXXXXXXXX      896
ORYZIAS_LATIPES                 ADMEGEPMYIIAAXKRNXXXXXXXXXXXXXXXXXXXXXXXXXXXXXXXXXXXXXXXXXXX      896
HIPPOCAMPUS_ERECTUS             SEGEGEPAFTIATXRGDPXXXXXXXXXXXXXXXXXXXXXXXXXXXXXXXXXXXXXXXXXX      896
CYNOGLOSSUS_SEMILAEVIS          AHGEGEPVYTLAAPRGDPXXXXXXXXXXXXXXXXXXXXXXXXXXXXXXXXXXXXXXXXXX      896
TAKIFUGU_RUBRIPES               AHGEGEPAFATATXRRDPXXXXXXXXXXXXXXXXXXXXXXXXXXXXXXXXXXXXXXXXXX      896
OREOCHROMIS_NILOTICUS           AEGEGEPIYTLATXRGDPXXXXXXXXXXXXXXXXXXXXXXXXXXXXXXXXXXXXXXXXXX      896
OREOCHROMIS_MOSSAMBICUS         AEGEGEPIYTLATXRGDPXXXXXXXXXXXXXXXXXXXXXXXXXXXXXXXXXXXXXXXXXX      896
CHANNA_PUNCTATA                 ADGEGEPMYTTAAXRRDPXXXXXXXXXXXXXXXXXXXXXXXXXXXXXXXXXXXXXXXXXX      896
CHANNA_ARGUS                    ADGEGEPMYTTATXRQEPXXXXXXXXXXXXXXXXXXXXXXXXXXXXXXXXXXXXXXXXXX      896
CHANNA_STRIATA                  ADGEGEPVYTTATXRRDQXXXXXXXXXXXXXXXXXXXXXXXXXXXXXXXXXXXXXXXXXX      896
DICENTRARCHUS_LABRAX            ADGEGEPIYTTATXRGDPXXXXXXXXXXXXXXXXXXXXXXXXXXXXXXXXXXXXXXXXXX      896
SCOMBER_JAPONICUS               VEGEGEPAYTTATXRGDPXXXXXXXXXXXXXXXXXXXXXXXXXXXXXXXXXXXXXXXXXX      896
EPINEPHELUS_COIOIDES            ADGEGEPVYAIASXRGDPXXXXXXXXXXXXXXXXXXXXXXXXXXXXXXXXXXXXXXXXXX      896
SPARUS_AURATA                   ADGEGEPVYATATXRGDPXXXXXXXXXXXXXXXXXXXXXXXXXXXXXXXXXXXXXXXXXX      896
SCOPHTHALMUS_MAXIMUS            AEGEGEPVFTIATXRADPXXXXXXXXXXXXXXXXXXXXXXXXXXXXXXXXXXXXXXXXXX      896
PARALICHTHYS_OLIVACEUS          ADGEGEPMYTIATXRGDPXXXXXXXXXXXXXXXXXXXXXXXXXXXXXXXXXXXXXXXXXX      896
                                 . ***     .  :       **************************************

PELTEOBAGRUS_FULVIDRACO         XXXX        900
DANIO_RERIO                     XXXX        900
CTENOPHARYNGODON_IDELLA         XXXX        900
HYPOPHTHALMICHTHYS_MOLITRIX     XXXX        900
ONCORHYNCHUS_MYKISS             XXXX        900
SALMO_SALAR                     XXXX        900
ORYZIAS_LATIPES                 XXXX        900
HIPPOCAMPUS_ERECTUS             XXXX        900
CYNOGLOSSUS_SEMILAEVIS          XXXX        900
TAKIFUGU_RUBRIPES               XXXX        900
OREOCHROMIS_NILOTICUS           XXXX        900
OREOCHROMIS_MOSSAMBICUS         XXXX        900
CHANNA_PUNCTATA                 XXXX        900
CHANNA_ARGUS                    XXXX        900
CHANNA_STRIATA                  XXXX        900
DICENTRARCHUS_LABRAX            XXXX        900
SCOMBER_JAPONICUS               XXXX        900
EPINEPHELUS_COIOIDES            XXXX        900
SPARUS_AURATA                   XXXX        900
SCOPHTHALMUS_MAXIMUS            XXXX        900
PARALICHTHYS_OLIVACEUS          XXXX        900
                                ****
```

S7B Fig

C

```
CYNOGLOSSUS_SEMILAEVIS          QKPNEKICVERCTRPQQXXXXXXXXXXXXXXXXXEFLGQRKGLQTGWFSASLSNSRRPTCL   60
PELTEOBAGRUS_FULVIDRACO         QNQLKKLMWRDVPNPNNCSWAKGIDFKKLDGXXNLFSHHEGLTAXCPLLPTSENVCEVEI   60
DANIO_RERIO                     QNQMKKLMWKDVPNPNKCSWAKGMDFRQIDTMESLFPHSEGLTAXCPLLLVSESICEVEI   60
CTENOPHARYNGODON_IDELLA         QNQMRKLVWKDVPNPNKCSWAKGMDFRQIDTMENLFPHSEGLTAXCPLLLVSESICEVEI   60
HYPOPHTHALMICHTHYS_MOLITRIX     QNQMRKLMWKDVPNPNKCSWAKGMDFRQIDTMENLFPHSEGLTAXCPLLLVSESICEVEI   60
ORYZIAS_LATIPES                 QNQIKRNLXXXVPNPKKCSWAKGIDFQKVDTFDXLFQPAEGLQIXCPLLPSDNIISKVII   60
ONCORHYNCHUS_MYKISS             QNHMKKFMWKDVPNPNNCSWAQGIDFGKADTMEQLFRHPEGLPAXWPLLLVSETISQATI   60
SALMO_SALAR                     QNHMKKFMWKDVPNPNNCSWAQGIDFGKADTMEQLFLHPEGLPAXWPLLLVSETISQATI   60
TAKIFUGU_RUBRIPES               QNQMKKLMWKDVPNPNQCSWARGIDLXXXNAFDHMFHPPEGFPAXWPLLLPPEKISNLVI   60
HIPPOCAMPUS_ERECTUS             QNQMKRFVWKDVPNPKKCSWAKGLNFKKADTFHELFQSSDILPAXWPLLLPSENISKVVI   60
OREOCHROMIS_NILOTICUS           QNQMKKFVSKDVPNPRKCSWAKGIDFKKVDTFDYLFRPREGLPVXWPLLMPSENISQVII   60
OREOCHROMIS_MOSSAMBICUS         QNQMKKFVSKDVPNPRKCSWAKGIDFKKVDTFDYLFRPREGLPVXWPLLMPSENISQVII   60
CHANNA_ARGUS                    QKQIRRLLWNDVPNPNNCSWAKGLDFKKADTFDHLFQSAEGLQAXWPLLPPSENISKVYV   60
CHANNA_PUNCTATA                 QNQMRSLVWKDVPNPNKCSWAKGLDFKKADNFDHLFQPVEGLQAXWPLLLPSENISKVIV   60
CHANNA_STRIATA                  QNQMRRFVWKDVPNPNKCSWAKGLDFKKAETFDNLFQPVEGLQAXWPLLLSPENISKVVL   60
SCOPHTHALMUS_MAXIMUS            QNQMKRFMWKDVPNPNKCSWAKGLDFKKADTFDHLFGPPEGLPAXWALLLPSEDLSKVVI   60
PARALICHTHYS_OLIVACEUS          QNQMKKFVWKDVPNPYKCSWAKGLDFKKNDTLDHLFGPSEGLPSXWSLLMPSENISKVVI   60
SCOMBER_JAPONICUS               QNQMKKFMWKDVPNPNKCSWAKGLDFKKADTFDHLFQPPEGLPAXRPLLLPSENISKVVI   60
EPINEPHELUS_COIOIDES            QNQMKRFVWKDVPNPNKCSWAKGLDLKKADNFDHLFQPAESLSAXWPLLLPAENISKVVI   60
DICENTRARCHUS_LABRAX            QNQMKKFMWKDVPNPNKCSWAKGLDFKKADTFDQLFRPPEGLPPXWPMLLPSENISKVVI   60
SPARUS_AURATA                   QNQMKKIVWKDVPNPNKCSWAKGLDFKKADNFDHLFRPPEGLSAXWPLLLPSENISKVVI   60
                                *:  .        .* :                 ::   . :          :      :

CYNOGLOSSUS_SEMILAEVIS          VXXXXXXXXXXXXXXXXX-XXXXXXAAAALREHIPRGHRXXXXXXXXXXXXXXXGQTPLH   119
PELTEOBAGRUS_FULVIDRACO         VEKXXXXXXXXXXXXXXXLFVLEDDQEEKALLXXXXXXXXXX---XXXXXXXXXXXHRSV   117
DANIO_RERIO                     IEKPHXXXXXXXXXXXXXXXX-XXXXXXXXXXXXPLTIENVKDNEEXXXXXXXXXXXXXX   119
CTENOPHARYNGODON_IDELLA         IEKCHXXXXXXXXXXXXXXXX-XXXXXXXXXXXXPLMLEHEKDXXXXXX--XXXXXNEVL   117
HYPOPHTHALMICHTHYS_MOLITRIX     IEKCHXXXXXXXXXXXXXXXX-XXXXXXXXXXXXPLMHEHEKDNXXXXX--XXXXXXXXE   117
ORYZIAS_LATIPES                 MEKVEXXXXKRAFMETQLMSL-NDDSVTSSSACLAPPFERSXXXXXXXXXXXXXCXXXXX   119
ONCORHYNCHUS_MYKISS             MEKTGXXXXXXXXXXXXXXXX-XXXXXXXXXXXXPPTSGSDKDLIPXXXXXXXXASSPAL   119
SALMO_SALAR                     MEKTGXXXXXXXXXXXXXXXX-XXXXXXXXXXXXPPTSVPDKDLIPXXXXXXXXASSPAL   119
TAKIFUGU_RUBRIPES               VDKADLSALSTPXXXXXXXXX-XPDPSVASSVRLHGEPDSPVGQAWPEESHLLPGGDRSS   119
HIPPOCAMPUS_ERECTUS             MEKANMSVLSGALVRSPLITP-TLDAVSCPFIXXXXXXXXEVDQXXXXXXXXXXNVIPSS   119
OREOCHROMIS_NILOTICUS           VDKVXXXXLTTALIQNPLXXX-XPDHADALAGSPSPGFDLNVDQXXFMENETLPVGGPSS   119
OREOCHROMIS_MOSSAMBICUS         VDKVXXXXLTTALIQNPLXXX-XPDHADALAGSHSPGFDLNVDQXXFMENETLPVGGPSS   119
CHANNA_ARGUS                    VEKAELSGLTSDLIQTPLVSL-SPDVADXXXXSHPLGFDSKGEQTQFMDREMLQGEVPSL   119
CHANNA_PUNCTATA                 VEKADLSDLTTALIQTPLVSL-SSDPAXXXXVPLTSVFDSKDEQTQFMNRELLLEGAASL   119
CHANNA_STRIATA                  VEKADLSGLTTALIQTPLVSL-SPDPAXXXXVSLPPGLEDQXXQTQFMGREMLPDGAPSL   119
SCOPHTHALMUS_MAXIMUS            VDKADLCAATTALVQTPLVPP-ATDPAAASAISLPPGFDSEVDHSELTESELLLGGAPPL   119
PARALICHTHYS_OLIVACEUS          MDKADLSAPTTALVQTPLVPL-TTDPATTLSISLHPGIDSEVDQGQFRESEVLLGGAPSL   119
SCOMBER_JAPONICUS               MEKTDLSALTXXXXXXPLVSL-NPDTATALAISIPSGFNSEVDQTRSMDSEALLGGAPSL   119
EPINEPHELUS_COIOIDES            VDEVDLSALTTALIQVPLVSL-TPDPDTALSISLPPGYDSEADQAQATESEVLLSGAPSL   119
DICENTRARCHUS_LABRAX            VDKVDLSALTTVLVQTPLVSL-TPDPATTLPXXXPPGFDSEVDQTPSLESEELQGGALSF   119
SPARUS_AURATA                   VDKVELSALTTALVQAPRVFL-TPDPASALSISIPPGFDSEVDQTQPMEGXXXXCGVPSF   119
                                :

CYNOGLOSSUS_SEMILAEVIS          SHQSH--FPSSVHRAGPQWGVXHAQRVDDGSSSVRSXXXSEHLNNVITEDRFIARFTNRP   177
PELTEOBAGRUS_FULVIDRACO         DTEAKSNNLSSIEGSLDPLSLXDTSTVSASPXXXDTXXXXXXXXSGQSSVRYSTILVFDX   177
DANIO_RERIO                     XLPSGDKTTTDSGLQGDSSXE--ALEASTAAPTPETXXXXXXXXSGQSSVTYSTILLSDX   177
CTENOPHARYNGODON_IDELLA         IYNSGNKANTDSACLGDSSEPXLSLQASTAAATPETXXXXXXXXSGQSSVTYSTVLLSDX   177
HYPOPHTHALMICHTHYS_MOLITRIX     VYNSGNKANTDSACLGDSSEPXLSLQASTAAATPETXXXXXXXXSGQSSVTYSTILLSDQ   177
ORYZIAS_LATIPES                 XXXLD--ASAPSSQSLDEANQXXXXXXADPIVPVDSXXXXXXXXSTSSSVRYAKLLLPCL   177
ONCORHYNCHUS_MYKISS             CVDSE--VXXXXXPGLPEEEXXESLQLPDLPRSLESXXXXXXXXSAQPSVTYATVLLSDX   177
SALMO_SALAR                     CVDSE--VXXXXXPGLPEEEXXETLQLPDLPRSLESXXXXXXXXSAQPSVTYATVLLSDD   177
TAKIFUGU_RUBRIPES               PPNLD--YPTGSAPXXXXXXXXDDGSCXXPAGVTDSXXXXXXXXSAQSSVIYTAVLLCGP   177
HIPPOCAMPUS_ERECTUS             ILDLD--ILTNPRPQLDELQLXXXXXIDPPPSVLENXXXXXXXXSAQSSVAYAAVLLTNA   177
OREOCHROMIS_NILOTICUS           AVDLD--TLTTSSSRADXLQPXADPSVDPHPGSTENXXXXXXXXSGQSSVTYIAVLVSNP   177
OREOCHROMIS_MOSSAMBICUS         AVDLD--TLTSSSSRADXLQPXADPSVNQHPGSTENXXXXXXXXSGQSSVTYIAVLVSNP   177
CHANNA_ARGUS                    ALNLD--ALXXXXXMMDELQSXNDSPEDQPQGDTFTXXXXXXXXSAQSSVTYATVLHSHP   177
CHANNA_PUNCTATA                 AVNLD--ALNNFHPEIDELQSXDPSPEVQPPGSTVSXXXXXXXXSAQSSVTYATVLISHP   177
CHANNA_STRIATA                  ALNLD--ALTSSHPKIEALQPXDDSPEDQPPGSTVSXXXXXXXXSAQSSVTYATVLLSQP   177
SCOPHTHALMUS_MAXIMUS            AVNAD--ALTGSYPRIDEFQPXVVSQAAQPPGSADNXXXXXXXXSAQSSVTYATVLLSDP   177
PARALICHTHYS_OLIVACEUS          DLNLD--ALTSSYPRIEELXXXXLPVVSQPPGSTDNXXXXXXXXSAQSSVTYITVLLSDP   177
SCOMBER_JAPONICUS               TLDMD--ALTASSLRTDELQLVDLSRTDQLPGTNNNXXXXXXXXSAQSSVTYATVLLINP   177
EPINEPHELUS_COIOIDES            AHDTD--ALTSSSPTTDQLQLXIHPLAEQXPGSTDSSAQSSAQNSAQSSVTYATVLLPDM   177
DICENTRARCHUS_LABRAX            ALNLD--DLTSSSPEIDQLELXDNLPADQSRGSTDSXXXXXXXXSAQSSVTYATVLLSDP   177
SPARUS_AURATA                   ALNLD--ALTSSSPVLDELQLXDAPPAERPPGSTDSXXXXXXXXSAQSSVTYATVLLSRP   177
                                                                   .        .   .  ::  :
```

```
CYNOGLOSSUS_SEMILAEVIS            APRQCRELCTVFSHIFHCDASHEGRATLXXXXXXXPLQGGEWQQXXXXXXXXXXXLQRR   237
PELTEOBAGRUS_FULVIDRACO           XXXXQXPVLQRKQQESLSSSSDEGNFSANNSDISGSFPGGLCELERQSSSDAIXXXNPRN   237
DANIO_RERIO                       XXXXQXPSQLQKQQESLSSSSDEGNFSANNSDISGSFPGGLWDLENSXXXXXXXXXNPRH   237
CTENOPHARYNGODON_IDELLA           XXXXQXPTLLRKQQESLSSSSDEGNFSANNSDISGSFPGGLWDLDNHVCSDSTXXXNPRH   237
HYPOPHTHALMICHTHYS_MOLITRIX       XXXXXXPTLLRKQQESLSSSSDEGNFSANNSDIFGSFPGGLWDLENHVCSDSTXXXNPRH   237
ORYZIAS_LATIPES                   KQEKQXPGNXPDKDGSGSNSSDEGNFSANNSEISESSPTGLWELDSCHSAEMDXXXDQRR   237
ONCORHYNCHUS_MYKISS               XXXXNXPHHLYKQEGSLSSSSDEGNFSGNNSDISGSFPGGLWELEISHSGAGESDLDPRR   237
SALMO_SALAR                       XXXXXXPHHLYKQEGSLSSSSDEGNFSGNNSDISGSFPGGLWELEISHSGTGESDLDPRH   237
TAKIFUGU_RUBRIPES                 KQQQQXHHHLHDKDCSCSSSSDEGNFSANNSDISASFNGGLWELDXXXXXXXXXXXVPRR   237
HIPPOCAMPUS_ERECTUS               KQELQXSIHLSDRDGSGSSSSDEGNFSADNSDISGSFPGGLWELESCHDREVDXXXDPRR   237
OREOCHROMIS_NILOTICUS             SQDQQPPIHLPYKDGSGNSSSDEGNFSANNSDISGSFPGGLWELESCRGLEMDXXXDPRR   237
OREOCHROMIS_MOSSAMBICUS           SQDQQPPIHRPYKDGSGNSSSDEGNFSANNSDISGSFPGGLWELESCRGLEMDXXXDPRR   237
CHANNA_ARGUS                      KQEQQXHLRLHYKDGCGSSSIDEGNFSANNSEISGSFPGGLWELDSCRGGEMEXXXDPRR   237
CHANNA_PUNCTATA                   KQDQQXHICLRYKDGSGSSSSDEGNFSANNSDISGSFPGGLWELDSCRGGESEXXXDQRR   237
CHANNA_STRIATA                    KQEQQXHICLHYKDGSGSSSSDEGNFSANNSDISGSFPGGLWELDSCRGGEMEXXXDPRR   237
SCOPHTHALMUS_MAXIMUS              KQEQPXPVHLHYKGSSGSSSSDEGNFSANNSDISGSFPGGLWELDSCRGGETDXXXDPRR   237
PARALICHTHYS_OLIVACEUS            KQEQPXPIHLHYKGGSGSSSSDEGNFSANNSDISGSFPGGLWELESCRGGEMDXXXDPRR   237
SCOMBER_JAPONICUS                 KQEQQXPIHLHYKDGSGSSSSDEGNFSANNSDISGSFPGGLWELESCRGGEMDXXXDPRR   237
EPINEPHELUS_COIOIDES              KQEQPXPIHLHYKDGSGSSSSDEGNFSANNSDISGSFPGGLWELDSCRGGEIDXXXDPRR   237
DICENTRARCHUS_LABRAX              KQEQQXPNHLHYKDGSGSSSSDEGNFSANNSDISGSFPGGLWELDXXXXXXXXXXXDPRR   237
SPARUS_AURATA                     KQEQQXPIHLHYKDGSGNSSSDEGNFSANNSDISGSFHGGLWELDXXXXXXXXXXXDPRR   237
                                                    .: .**. :            *  :               *.

CYNOGLOSSUS_SEMILAEVIS            QFLCQLRHLXRIHPMWPAGAGQLPGRRXXGGRPSTFLLLQLCGGTFNIGAGRRSXXXXXX   297
PELTEOBAGRUS_FULVIDRACO           SXXCSYNSVEEFSETSEQEDEAXXXXXXXSESIQVSK------ELYYIGMNXXEEEEEDE   291
DANIO_RERIO                       SXXSSYNSVEEFSETSEPDYEAXXXXXXXSENTGLAK------DLYYLEMTGEEKEEEEE   291
CTENOPHARYNGODON_IDELLA           SXXSSYNSVEEFSETSEQDYEAXXXXXXXSESTGVAK------DLYYLEMNEEEKQEEDE   291
HYPOPHTHALMICHTHYS_MOLITRIX       SXXSSYNSVEEFSETSEQDYEAXXXXXXXSESPGVAK------DLYYLEMNEEEKQEEDE   291
ORYZIAS_LATIPES                   FXXCSYASEGELSEISEHEAXXXXXXXXXVMEQRREQ------TLCYLQIGYPDEDEESA   291
ONCORHYNCHUS_MYKISS               SXXCSYNSIEEFSETSEQEDEALGGERDGGIEIIEEK------DLYYLGMDCQEESEEEK   291
SALMO_SALAR                       SXXCSYNSVEEFSETSEQEDEALGGERDGGIEVIEEK------DLYYLGMGYQEESEGEE   291
TAKIFUGU_RUBRIPES                 SXXCCYNSTEELSEKPEQGDXXXXXXXXXRDVGEEK------DLYYIGADYGDEDEESE   291
HIPPOCAMPUS_ERECTUS               SXXCSYNSVEELSEPSDQEDXXXXXXXXXXXATEEK------DLYYLSMDYQAENEEGE   291
OREOCHROMIS_NILOTICUS             SXXCSYNSVEELSQNSDQEEXXXXXXXXXEHEVRREK------DLYYLGMDYPAEDEESE   291
OREOCHROMIS_MOSSAMBICUS           SXXCSYNSVEELSQNSDQEEXXXXXXXXXEHEVRREK------DLYYLGMDYPAEDEESE   291
CHANNA_ARGUS                      SXXCSYNSVEELSKTSEQGDXXXXXXXXXEGDMKEGK------DXXFLEMDHPAKEEENK   291
CHANNA_PUNCTATA                   SXXCSYNSVEELSETSEQEDXXXXXXXXXEGGTKEGK------NLYYLGMDYPAEDEDSE   291
CHANNA_STRIATA                    SXXCSYNSVEELSETSEQEDXXXXXXXXXEGDAKEGK------DLYYLGMDYPAEDEESE   291
SCOPHTHALMUS_MAXIMUS              SXXCSYNSVEELYETSEQEDXXXXXXXXXDGEARDEK------DLYYLGMDYPAEDEASE   291
PARALICHTHYS_OLIVACEUS            SXXCSYNSVEELSETSEQEDXXXXXXXXXEGEGRGEK------DLYYLGTDYPAEDEESE   291
SCOMBER_JAPONICUS                 SXXCSYNSVEELSETSEQEDXXXXXXXXXXEEVREGK------HLYYLGMDYPAEDEESD   291
EPINEPHELUS_COIOIDES              SXXCSYNSVEELSETSEQEDEXXXXXXXXEEEAREEK------DLYYLGMDYPAEDEESE   291
DICENTRARCHUS_LABRAX              SXXRSYNSVEELSETSEQGDXXXXXXXXXEEAVREEK------DLYYLGMDYRAEDEESE   291
SPARUS_AURATA                     SXXCSYNSVEFLSETSEQGDXXXXXXXXXGEEAREEK------DLYYLGMDYRAEDEDSE   291
                                             .:                                    :

CYNOGLOSSUS_SEMILAEVIS            XXR---------KREGLVL----SQSGXVSRGGRGGGRGGEGGQVAQRRCCXEIKSTQXXXX   346
PELTEOBAGRUS_FULVIDRACO           LEEKFKEDDATKEQIXXXXXXXX-----XXX--------------------------------   317
DANIO_RERIO                       EEEEEEEEDEPHEGQSKNKXXXX-----XXXXX-------XXXXXRVMGVNPR-------   332
CTENOPHARYNGODON_IDELLA           DIKEAQ-----DXGQDKNEIXXXXXXVMRXXXX-------XXXXXXXXXVDAR-------   332
HYPOPHTHALMICHTHYS_MOLITRIX       DIEEAQ-----DXXXXXXXXXKNEIXVMRXXXX-------XXXXXXXXXVGAR-------   332
ORYZIAS_LATIPES                   EEVQREEEEKRKEQPAK-----DASXLNGXXXX-------XXXXXKDFFVXXX-------   332
ONCORHYNCHUS_MYKISS               XXXXXXXKEDTRAMLLK-----EVMVLGRXXXX-------XXXXXGGSSVESI-------   332
SALMO_SALAR                       EEEEKEEKEDTGAMLLK-----EVMVLGRXXXX-------XXXXXEGSSVESI-------   332
TAKIFUGU_RUBRIPES                 XXXXXXXKEELNAKLIQ-----TVPXLNSXXXX-------XXXXXEGCSAESR-------   332
HIPPOCAMPUS_ERECTUS               GEVKQKEXXXXXXELLK-----SVTXLSRXXXX-------XXXXXDGCSVESH-------   332
OREOCHROMIS_NILOTICUS             KEDGQSEDEEAKVELLK-----STPXLNRXXXX-------XXXXXGHCSLELH-------   332
OREOCHROMIS_MOSSAMBICUS           KEDGQSEDEEAKVELLK-----STPXLNRXXXX-------XXXXXGHCSLELH-------   332
CHANNA_ARGUS                      EEQDKEQKSKEETKMFK-----NVVXLNIXXXX-------XXXXXESCSVELQ-------   332
CHANNA_PUNCTATA                   EEEEQREKEETKIELLK-----IVVXPDQXXXX-------XXXXXXSFSGESR-------   332
CHANNA_STRIATA                    EEEEQREKEETKIELLK-----NVVXLDMXXXX-------XXXXXESCSVELH-------   332
SCOPHTHALMUS_MAXIMUS              DEDERREKEAAKIDLRK-----NVVXLTRXXXX-------XXXXXDDCSVESR-------   332
PARALICHTHYS_OLIVACEUS            DEXXXXXKEEQREELRK-----NVVXLSRXXXX-------XXXXXEDCCVESR-------   332
SCOMBER_JAPONICUS                 EEEEQREKEDTKTELLT-----SVVXLNRXXXX-------XXXXXDACSVESH-------   332
EPINEPHELUS_COIOIDES              EDKEQIEKDXXXIELLK-----NVVXLSRXXXX-------XXXXXEDCSVELH-------   332
DICENTRARCHUS_LABRAX              EDEEQREKEETKIELLK-----NVVXLNRXXXX-------XXXXXEDCSVESH-------   332
SPARUS_AURATA                     EGEEEREKEETRAELLK-----SVVXLSRXXXX-------XXXXXEDCPVESH-------   332
```

```
CYNOGLOSSUS_SEMILAEVIS           XXXXKYTFEQXXXKLLXRGVTASAEGRGPRHTGGDPRLTAVPPSVQNCSMCEA-------   399
PELTEOBAGRUS_FULVIDRACO          ---------------------------------------XXXXXXXSDSGDSNVPLYXLP   338
DANIO_RERIO                      ----------------------------PLLESQNSTAXXXXXXSNSNNMSHSIPLYLXP   364
CTENOPHARYNGODON_IDELLA          ----------------------------PLLEGKNSTVXXXXXXVNSNNVSRSIPLYLXP   364
HYPOPHTHALMICHTHYS_MOLITRIX      ----------------------------PLLEGKNSTIXXXXXXVDSNNVSPSIPLYLXP   364
ORYZIAS_LATIPES                  ----------------------------PLIXXXXXXXXXXXXXXXXCDLSSXXXQYMXP   364
ONCORHYNCHUS_MYKISS              ----------------------------PLLGSQDSMFSEYSDEGSVVGMRSXVPLYLXP   364
SALMO_SALAR                      ----------------------------PLLGSQDSMFSEYSDEGLVVGMRSXVPLYLXP   364
TAKIFUGU_RUBRIPES                ----------------------------RLLELTESXXXXXXXXXXKCDFSPXXXLYLXP   364
HIPPOCAMPUS_ERECTUS              ----------------------------LTPTELVSTSXXXXXXXXTCDLAPXXXLYMXP   364
OREOCHROMIS_NILOTICUS            ----------------------------PLLGQDNPSEPGILPSPSTCGFAMXXXPYLXP   364
OREOCHROMIS_MOSSAMBICUS          ----------------------------PLLGQDNPSEPGILPSPSTCGFAMXXXPYLXP   364
CHANNA_ARGUS                     ----------------------------PLLSPKEPTKXXXPLSASTCGFSEXXXLYLXP   364
CHANNA_PUNCTATA                  ----------------------------PLLGSGEPTEXXXPLSASTCGFTPXXXLYLXP   364
CHANNA_STRIATA                   ----------------------------PLLGPGGPTEXXXPLSASTRGFSPXXXLYLXP   364
SCOPHTHALMUS_MAXIMUS             ----------------------------PLLCPGDSSSRTGPLSASTRGFTPXXXLYLXP   364
PARALICHTHYS_OLIVACEUS           ----------------------------PLLDPEDSSGXXXPMSDSTGGLSPXXXLYLXP   364
SCOMBER_JAPONICUS                ----------------------------PLLGPVDSSDNSEMDSASTRGFSLXXXLYMXP   364
EPINEPHELUS_COIOIDES             ----------------------------PLLSPEDSSEXXXLLLASTRGFSSXXXLYLXP   364
DICENTRARCHUS_LABRAX             ----------------------------PLLTPEDSSEXXXLLSASTRGFCPXXXLYLXP   364
SPARUS_AURATA                    ----------------------------PLLGAEDSSEXXXXXXXXPRGFSLXXXLYMXP   364
                                                                                 .

CYNOGLOSSUS_SEMILAEVIS           ---------------TVRE---------PNSAVXXXXXXXXXXXXXXXXXXXXXXXXXXX   435
PELTEOBAGRUS_FULVIDRACO          QFQTAAIKLLREPAGNSTXXXIQLXXXXXXXXXXXXXXXXXXXXXXXXXXXXXXXXXXXX   398
DANIO_RERIO                      QFRSECXXXXXINPXXXT----XXXXXXXXXXXXXXXXXXXXXXXXXXXXXXXXXXXXXX   420
CTENOPHARYNGODON_IDELLA          QFQTECXXXXXINPXXXP----XXXXXXXXXXXXXXXXXXXXXXXXXXXXXXXXXXXXXX   420
HYPOPHTHALMICHTHYS_MOLITRIX      QFQTECXXXXXINPXXXP----XXXXXXXXXXXXXXXXXXXXXXXXXXXXXXXXXXXXXX   420
ORYZIAS_LATIPES                  QYRTAAYRSQLVXXXXXX----XXXXXXXXXXXXXXXXXXXXXXXXXXXXXXXXXXXXXX   420
ONCORHYNCHUS_MYKISS              QFRTAPXXXXXXXXXXXX----XXXSSPLKAQDSXXXXXXXXXXXXXXXXXXXXXXXXXX   420
SALMO_SALAR                      QFRTVPXXXXXXXXXXXX----XXXSSPLKAQDSAHQLXXXXXXXXXXXXXXXXXXXXXX   420
TAKIFUGU_RUBRIPES                QFRTAPXXXSXXCTXXXR----QLSAKPQEGRCHPXXXXXXXXXXXXXXXXXXXXXXXXX   420
HIPPOCAMPUS_ERECTUS              QFRTAPXXXXXXYSXXXR----QLPDXXXXXXXXXXXXXXXXXXXXXXXXXXXXXXXXXX   420
OREOCHROMIS_NILOTICUS            QFRTATXXXXXXCTXXXA----QHTQREPQLXXXXXXXXXXXXXXXXXXXXXXXXXXXXX   420
OREOCHROMIS_MOSSAMBICUS          QFRTATXXXXXXCTXXXA----QHTQREPQLXXXXXXXXXXXXXXXXXXXXXXXXXXXXX   420
CHANNA_ARGUS                     QFRTLPXXXXXXCTXXXX----XXXSQLQDSQVXXXXXXXXXXXXXXXXXXXXXXXXXXX   420
CHANNA_PUNCTATA                  QFRTVPXXXXXXCTXXXX----XXXNQLQDSKAQIXXXXXXXXXXXXXXXXXXXXXXXXX   420
CHANNA_STRIATA                   QFRTVPXXXXXXCAXXXX----XXXNQPQDSEAQLXXXXXXXXXXXXXXXXXXXXXXXXX   420
SCOPHTHALMUS_MAXIMUS             QFRTAPXXXXXXCTXXXR----QLQDSEPELXXXXXXXXXXXXXXXXXXXXXXXXXXXXX   420
PARALICHTHYS_OLIVACEUS           QFRTAAXXXXXXCTXXXR----QLQDSRTQLXXXXXXXXXXXXXXXXXXXXXXXXXXXXX   420
SCOMBER_JAPONICUS                QFRTAPXXXXXXCTXXXR----QLTDXXXXXXXXXXXXXXXXXXXXXXXXXXXXXXXXXX   420
EPINEPHELUS_COIOIDES             QFRTAPXXXXXXYTXXXR----QLTAQPHDGKPQQXXXXXXXXXXXXXXXXXXXXXXXXX   420
DICENTRARCHUS_LABRAX             QFRTAPXXXXXXNTXXXR----QLTAQPQDSKPQVXXXXXXXXXXXXXXXXXXXXXXXXX   420
SPARUS_AURATA                    QFRTAPXXXXXXCTXXXR----QVAAQPQDAPAASLAQTAFKXXXXXXXXXXXXXXXXXX   420
                                                                           ******************

CYNOGLOSSUS_SEMILAEVIS           XXXXXXXXXXXXXXXXXXXXXXXXXXXXXXXXXXXXXXXXXXXXXXXXXXXXXXXXXXXX   495
PELTEOBAGRUS_FULVIDRACO          XXXXXXXXXXXXXXXXXXXXXXXXXXXXXXXXXXXXXXXXXXXXXXXXXXXXXXXXXXXX   458
DANIO_RERIO                      XXXXXXXXXXXXXXXXXXXXXXXXXXXXXXXXXXXXXXXXXXXXXXXXXXXXXXXXXXXX   480
CTENOPHARYNGODON_IDELLA          XXXXXXXXXXXXXXXXXXXXXXXXXXXXXXXXXXXXXXXXXXXXXXXXXXXXXXXXXXXX   480
HYPOPHTHALMICHTHYS_MOLITRIX      XXXXXXXXXXXXXXXXXXXXXXXXXXXXXXXXXXXXXXXXXXXXXXXXXXXXXXXXXXXX   480
ORYZIAS_LATIPES                  XXXXXXXXXXXXXXXXXXXXXXXXXXXXXXXXXXXXXXXXXXXXXXXXXXXXXXXXXXXX   480
ONCORHYNCHUS_MYKISS              XXXXXXXXXXXXXXXXXXXXXXXXXXXXXXXXXXXXXXXXXXXXXXXXXXXXXXXXXXXX   480
SALMO_SALAR                      XXXXXXXXXXXXXXXXXXXXXXXXXXXXXXXXXXXXXXXXXXXXXXXXXXXXXXXXXXXX   480
TAKIFUGU_RUBRIPES                XXXXXXXXXXXXXXXXXXXXXXXXXXXXXXXXXXXXXXXXXXXXXXXXXXXXXXXXXXXX   480
HIPPOCAMPUS_ERECTUS              XXXXXXXXXXXXXXXXXXXXXXXXXXXXXXXXXXXXXXXXXXXXXXXXXXXXXXXXXXXX   480
OREOCHROMIS_NILOTICUS            XXXXXXXXXXXXXXXXXXXXXXXXXXXXXXXXXXXXXXXXXXXXXXXXXXXXXXXXXXXX   480
OREOCHROMIS_MOSSAMBICUS          XXXXXXXXXXXXXXXXXXXXXXXXXXXXXXXXXXXXXXXXXXXXXXXXXXXXXXXXXXXX   480
CHANNA_ARGUS                     XXXXXXXXXXXXXXXXXXXXXXXXXXXXXXXXXXXXXXXXXXXXXXXXXXXXXXXXXXXX   480
CHANNA_PUNCTATA                  XXXXXXXXXXXXXXXXXXXXXXXXXXXXXXXXXXXXXXXXXXXXXXXXXXXXXXXXXXXX   480
CHANNA_STRIATA                   XXXXXXXXXXXXXXXXXXXXXXXXXXXXXXXXXXXXXXXXXXXXXXXXXXXXXXXXXXXX   480
SCOPHTHALMUS_MAXIMUS             XXXXXXXXXXXXXXXXXXXXXXXXXXXXXXXXXXXXXXXXXXXXXXXXXXXXXXXXXXXX   480
PARALICHTHYS_OLIVACEUS           XXXXXXXXXXXXXXXXXXXXXXXXXXXXXXXXXXXXXXXXXXXXXXXXXXXXXXXXXXXX   480
SCOMBER_JAPONICUS                XXXXXXXXXXXXXXXXXXXXXXXXXXXXXXXXXXXXXXXXXXXXXXXXXXXXXXXXXXXX   480
EPINEPHELUS_COIOIDES             XXXXXXXXXXXXXXXXXXXXXXXXXXXXXXXXXXXXXXXXXXXXXXXXXXXXXXXXXXXX   480
DICENTRARCHUS_LABRAX             XXXXXXXXXXXXXXXXXXXXXXXXXXXXXXXXXXXXXXXXXXXXXXXXXXXXXXXXXXXX   480
SPARUS_AURATA                    XXXXXXXXXXXXXXXXXXXXXXXXXXXXXXXXXXXXXXXXXXXXXXXXXXXXXXXXXXXX   480
                                 ************************************************************

CYNOGLOSSUS_SEMILAEVIS           XX-XXXX-XX-X----------XXXXXX----XXXX-XXXXX-XXX-X-X----------   524
PELTEOBAGRUS_FULVIDRACO          XXXXXXXXXXXXXXXXXXXXXXXXXXXXXXXXXXXXXXXXXXXXXXXXXXXXXXXXXXXX   518
DANIO_RERIO                      XXXXXXXXXXXXXXXXXXXXX-XXXXXXXXX-XXXX-XX-XX-X-X-X-X---X------   523
CTENOPHARYNGODON_IDELLA          XXXXXXXXXXXXXXXXXXXXX-XXXXXXXXX-XXXX-XX-XX-X-X-X-X---X------   523
HYPOPHTHALMICHTHYS_MOLITRIX      XXXXXXXXXXXXXXXXXXXXX-XXXXXXXXX-XXXX-XX-XX-X-X-X-X---X------   523
ORYZIAS_LATIPES                  XXXXXXXXXXXXXXXXXXXXX-XXXXXXXXX-XXXX-XX-XX-X-X-X-X---X------   523
ONCORHYNCHUS_MYKISS              XXXXXXXXXXXXXXXXXXXXX-XXXXXXXXX-XXXX-XX-XX-X-X-X-X---X------   523
SALMO_SALAR                      XXXXXXXXXXXXXXXXXXXXX-XXXXXXXXX-XXXX-XX-XX-X-X-X-X---X------   523
TAKIFUGU_RUBRIPES                XXXXXXXXXXXXXXXXXXXXX-XXXXXXXXX-XXXX-XX-XX-X-X-X-X---X------   523
HIPPOCAMPUS_ERECTUS              XXXXXXXXXXXXXXXXXXXXX-XXXXXXXXX-XXXX-XX-XX-X-X-X-X---X------   523
OREOCHROMIS_NILOTICUS            XXXXXXXXXXXXXXXXXXXXX-XXXXXXXXX-XXXX-XX-XX-X-X-X-X---X------   523
OREOCHROMIS_MOSSAMBICUS          XXXXXXXXXXXXXXXXXXXXX-XXXXXXXXX-XXXX-XX-XX-X-X-X-X---X------   523
CHANNA_ARGUS                     XXXXXXXXXXXXXXXXXXXXX-XXXXXXXXX-XXXX-XX-XX-X-X-X-X---X------   523
CHANNA_PUNCTATA                  XXXXXXXXXXXXXXXXXXXXX-XXXXXXXXX-XXXX-XX-XX-X-X-X-X---X------   523
CHANNA_STRIATA                   XXXXXXXXXXXXXXXXXXXXX-XXXXXXXXX-XXXX-XX-XX-X-X-X-X---X------   523
SCOPHTHALMUS_MAXIMUS             XXXXXXXXXXXXXXXXXXXXX-XXXXXXXXX-XXXX-XX-XX-X-X-X-X---X------   523
PARALICHTHYS_OLIVACEUS           XXXXXXXXXXXXXXXXXXXXX-XXXXXXXXX-XXXX-XX-XX-X-X-X-X---X------   523
```

```
SCOMBER_JAPONICUS               XXXXXXXXXXXXXXXXXXXXX-XXXXXXXXX-XXXX-XX-XX-X-X-X-X---X------  523
EPINEPHELUS_COIOIDES            XXXXXXXXXXXXXXXXXXXXX-XXXXXXXXX-XXXX-XX-XX-X-X-X-X---X------  523
DICENTRARCHUS_LABRAX            XXXXXXXXXXXXXXXXXXXXX-XXXXXXXXX-XXXX-XX-XX-X-X-X-X---X------  523
SPARUS_AURATA                   XXXXXXXXXXXXXXXXXXXXX-XXXXXXXXX-XXXX-XX-XX-X-X-X-X---X------  523
                                ** **** ** *          ******    **** ** ** * * * *

CYNOGLOSSUS_SEMILAEVIS          ------         524
PELTEOBAGRUS_FULVIDRACO         XXXXXX         524
DANIO_RERIO                     X-----         524
CTENOPHARYNGODON_IDELLA         X-----         524
HYPOPHTHALMICHTHYS_MOLITRIX     X-----         524
ORYZIAS_LATIPES                 X-----         524
ONCORHYNCHUS_MYKISS             X-----         524
SALMO_SALAR                     X-----         524
TAKIFUGU_RUBRIPES               X-----         524
HIPPOCAMPUS_ERECTUS             X-----         524
OREOCHROMIS_NILOTICUS           X-----         524
OREOCHROMIS_MOSSAMBICUS         X-----         524
CHANNA_ARGUS                    X-----         524
CHANNA_PUNCTATA                 X-----         524
CHANNA_STRIATA                  X-----         524
SCOPHTHALMUS_MAXIMUS            X-----         524
PARALICHTHYS_OLIVACEUS          X-----         524
SCOMBER_JAPONICUS               X-----         524
EPINEPHELUS_COIOIDES            X-----         524
DICENTRARCHUS_LABRAX            X-----         524
SPARUS_AURATA                   X-----         524
```

S7C Fig
