# Supplementary material for: In silico analyses of leptin and leptin receptor of spotted snakehead Channa punctata
Source: PLoS One. 2022 Jul 7;17(7):e0270881. doi: 10.1371/journal.pone.0270881 (PMC9262212; doi:10.1371/journal.pone.0270881)
Supplement: S1 Table — (DOCX) [file pone.0270881.s008.docx]

**S1 Table** Quality assessment of tertiary structure of leptin and leptin receptor of *C. punctata*

| Position of residues | Lepa | ECD Lepr | ICD Lepr |
| --- | --- | --- | --- |
| Residues in most favoured region | 93.4% | 58.3% | 45.3% |
| Residues in additional allowed region | 5% | 26.4% | 36.3% |
| Residues in generously allowed region | 1.7% | 6.8% | 9.7% |
| Residues in disallowed region | 0% | 8.4% | 8.7% |

Proportion of amino acids falling in most favoured/ additionally allowed/ generously allowed/ disallowed region in the Ramachandran plot for leptin paralog a (Lep), and extra- (ECD) and intra- (ICD) cellular domains of leptin receptor (Lepr).
