# Supplementary material for: In silico analyses of leptin and leptin receptor of spotted snakehead Channa punctata
Source: PLoS One. 2022 Jul 7;17(7):e0270881. doi: 10.1371/journal.pone.0270881 (PMC9262212; doi:10.1371/journal.pone.0270881)
Supplement: S2 Table — (DOCX) [file pone.0270881.s009.docx]

**S2 Table** NCBI Accession number of protein and nucleotide sequences of leptin and its receptor of vertebrates

| Vertebrate Class | Organism | Leptin a | | Leptin b | Leptin receptor | |
| --- | --- | --- | --- | --- | --- | --- |
|  |  | Protein | Nucleotide | Protein | Protein | Nucleotide |
| Pisces | *Channa punctata* | QCO95272.1 | MK039679.1 | - | QCO95273.1 | MK039680.1 |
|  | *Channa striata* | SSC14288.1 | LS974310.1 | - | SSC14289.1 | LS974311.1 |
|  | *Ctenopharyngodon idella* | ACI32423.1 | EU719623.1 | AFU35432.1 | AFU35431.1 | JQ080547.1 |
|  | *Cynoglossus semilaevis* | ARX70258.1 | KY432435.1 | ATX63221.1 | ARX70259.1 | KY432436.1 |
|  | *Danio rerio* | NP_001122048.1 | NM_001128576.1 | - | NP_001296332.1 | NM_001309403.1 |
|  | *Dicentrarchus labrax* | AIY99976.1 | KJ934255.1 | - | AIB06819.1 | KF918755.1 |
|  | *Epinephelus coioides* | AFU55260.1 | JX406147.1 | AFU55261.1 | AFU55262.1 | JX406149.1 |
|  | *Hippocampus erectus* | AMB73110.1 | KP888952.1 | - | AMB73111.1 | KP888953.1 |
|  | *Hypophthalmichthys molitrix* | ACI32424.1 | EU719624.1 | - | AJT58572.1 | KM068181.1 |
|  | *Oncorhynchus mykiss* | BAG09232.1 | AB354909.1 | XP_021419703.1 | AGC55253.1 | XP_021460283.1 |
|  | *Oreochromis mossambicus* | AGT28752.1 | KC354702.1 | - | AGT28753.1 | KC354703.1 |
|  | *Oreochromis niloticus* | AHL37667.1 | KF955988.1 | AHL37668.1 | AHL37669.1 | KF955990.1 |
|  | *Oryzias latipes* | BAD94448.2 | AB193548.2 | - | NP_001153915.1 | AB457590.2 |
|  | *Paralichthys olivaceus* | AVC04905.1 | KY798115.1 | - | XP_019953845.1 | XM_020098286.1 |
|  | *Salmo salar* | ACZ02412.1 | FJ830677.1 | - | NP_001158237.1 | NM_001164765.1 |
|  | *Scomber japonicus* | AKM16801.1 | KP635449.1 | AKM16802.1 | AKM16803.1 | KP635451.1 |
|  | *Scophthalmus maximus* | AKA88531.1 | KP197049.1 | AWO98111.1 | AKA88532.1 | KP197050.1 |
|  | *Tachysurus fulvidraco* | AFO67938.1 | JQ288727.1 | - | AFO67946.1 | JX118825.1 |
|  | *Takifugu rubripes* | BAD94444.1 | AB193547.1 | - | BAG67079.1 | AB385663.1 |
|  | *Megalobrama amblycephala* | - | - | AIY34726.1 | - | - |
|  | *Siniperca chuatsi* | AHH86062.1 | - | ALB25877.1 | - | - |
|  | *Labeo rohita* | - | - | RXN17801.1 | - | - |
|  | *Channa argus* | ASW18438.1 | MF504015.1 | QCX08924.1 | - | KAF3690676.1 |
|  | *Morone saxitilis* | KAG7522506.1 | - | XP_035534060.1 | - | - |
|  | *Clupea harengus* | XP_042563328.1 | - | XP_012687378.2 |  | XP_031430874.1 |
|  | *Astyanax mexicanus* | KAG9273033.1 | - | - |  | KAG9277325.1 |
|  | *Chanos chanos* | QNG41929.1 | - | - |  | XP_030647005.1 |
|  | *Labeo rohita* | RXN24711.1 | - | RXN17801.1 |  | - |
|  | *Pimephales promelas* | KAG1929457.1 | - | KAG1953293.1 |  | KAG1941036.1 |
|  | *Carassius auratus* | ACL68083.1 | - | - |  | - |
|  | *Schizothorax prenanti* | AIE45855.1 | - | - |  | - |
|  | *Cyprinus carpio* | XP_018981870.1 | - | XP_042578069.1 |  | - |
|  | *Megalobrama amblycephala* | - | - | - |  | - |
|  | *Solea senegalensis* | KAG7522506.1 | - | XP_043876192.1 |  | KAG7515062.1 |
|  | *Anabas testudineus* | - | - | XP_026219629.1 |  | XP_026201003.1 |
|  | *Epinephelus lanceolatus* | - | - | XP_033471257.1 |  | - |
|  | *Salvelinus alpinus* | BAH83535.1 | - | - |  | - |
|  | *Tetraodon nigroviridis* | BAD94451.1 | - | - |  | - |
|  | *Larimichthys crocea* | TMS05864.1 | - | - |  | - |
|  | *Thunnus thynnus* | ADT91717.1 | - | - |  | - |
|  | *Sparus aurata* | AWX49627.1 | - | - | AWX49626.1 | - |
|  | *Lateolabrax maculatus* | QFQ51510.1 | - | - | - | - |
|  | *Lateolabrax japonicus* | AHI85768.1 | - | - | - | - |
|  | *Betta splendens* | - | - | - | XP_040926394.1 | - |
|  | *Seriola dumerili* | - | - | - | XP_022601505.1 | - |
|  | *Thunnus albacares* | - | - | - | XP_044217474.1 | - |
|  | *Sander lucioperca* | - | - | - | XP_031139800.2 | - |
|  | *Perca flavescens* | - | - | - | XP_028442652.1 | - |
|  | *Fundulus heteroclitus* | - | - | - | XP_035996800.1 | - |
|  | *Oryzias melastigma* | - | - | - | KAF6734407.1 | - |
|  | *Salmo trutta* | - | - | - | XP_029563811.1 | - |
| Elasmobranchs | *Scyliorhinus canicula* | XP_038667989.1 | - | - | - | - |
|  | *Carcharodon carcharias* | XP_041059064.1 | - | - | - | - |
| Amphibian | *Xenopus tropicalis* | XP_002931881.2 | - | - | ABD63000.2 | - |
|  | *Xenopus laevis* | AAX77665.1 | - | - | XP_018116241.1 | - |
| Reptile | *Chelonia mydas* | EMP42339.1 | - | - | XP_007063480.1 | - |
|  | *Pogona vitticeps* | XP_020659433.1 | - | - | XP_020661756.1 | - |
|  | *Python bivittatus* | XP_025032399.1 | - | - | - | - |
|  | *Thamnophis elegans* | - | - | - | XP_032073703.1 | - |
| Aves | *Anas platyrhynchos* | NP_001337009.1 | - | - | ACF17729.1 | - |
|  | *Gallus gallus* | APC23099.1 | - | - | - | - |
|  | *Columba livia* | CDL67225.1 | - | - | - | - |
|  | *Passer montanus* | XP_039560475.1 | - | - | - | - |
|  | *Parus major* | - | - | - | XP_033372393.1 | - |
|  | *Amazona aestiva* | - | - | - | KQL60834.1 | - |
|  | *Coturnix coturnix* | - | - | - | AID21692.1 | - |
| Mammal | *Homo sapiens* | AAH69452.1 | - | - | AAB09673.1 | - |
|  | *Mus musculus* | ADM72802.1 | - | - | AAC52705.1 | - |
|  | *Canis lupus familiaris* | BAA35129.1 | - | - | NP_001019805.1 | - |
|  | *Sus scrofa* | ADK62398.1 | - | - | ACT52816.1 | - |
|  | *Ovis aries* | CCE35540.1 | - | - | NP_001009763.1 | - |
|  | *Bubalus bubalis* | AAS86311.1 | - | - | AOT28253.2 | - |
|  | *Interleukin-1 family member 10 [Homo sapiens]* | NP_115945.4 | - | - | - | - |
|  | *Ciliary neurotrophic factor receptor subunit alpha preproprotein [Mus musculus]* | - | - | - | NP_001129528.1 | - |
