# Supplementary material for: In silico analyses of leptin and leptin receptor of spotted snakehead Channa punctata
Source: PLoS One. 2022 Jul 7;17(7):e0270881. doi: 10.1371/journal.pone.0270881 (PMC9262212; doi:10.1371/journal.pone.0270881)
Supplement: S5 Fig — Ramachandran plot for A leptin a, B extracellular domain of leptin receptor and C intracellular domain of leptin receptor. Plot areas A, B and L represent the residues in the most favoured region; a, b, l, p represent the residues in additional allowed region; ~a, ~b, ~l, ~p represent the residues in generously allowed region. Glycine is represented by triangles. (PDF) [file pone.0270881.s005.pdf]

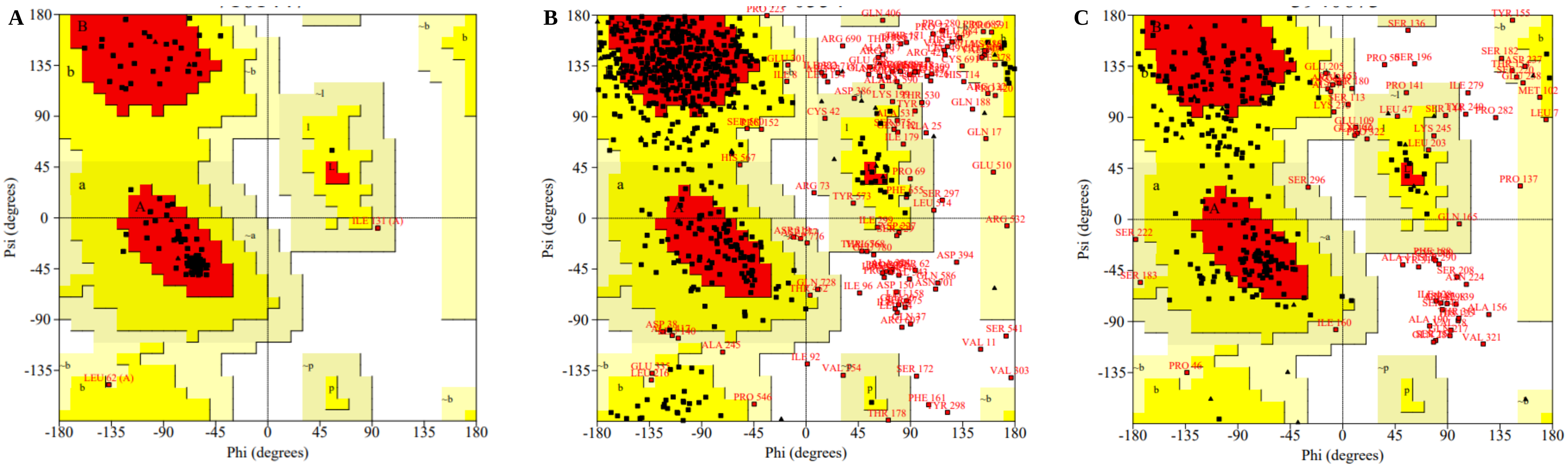

S5 Fig
